# Integrating scanned literature and online sources to build a data text collection for plant biodiversity

**DOI:** 10.3897/BDJ.14.e191990

**Published:** 2026-09-24

**Authors:** Nicolas Turenne, Eric Chenin, Youcef Sklab, Jean-Daniel Zucker

**Affiliations:** 1 IRD, Sorbonne Université, UMMISCO, Paris, France IRD, Sorbonne Université, UMMISCO Paris France; 2 INRAE, MathNum, Paris, France INRAE, MathNum Paris France; 3 ISYEB, MNHN, Paris, France ISYEB, MNHN Paris France

**Keywords:** Plant biodiversity, Botanical Text mining, Web and PDF text dataset, Optical Character Recognition (OCR), Species name recognition, Morphological trait extraction

## Abstract

**Background:**

Plant taxonomic descriptions are essential for botany, but remain scattered in unstructured texts. Automatic trait extraction is difficult due to linguistic variation, specialised terminology and the need for large, structured datasets.

**New information:**

**Methods**: We developed WordGen, an algorithm for species name recognition tolerant to OCR and typographical errors, using curated botanical dictionaries to reliably identify species sections in noisy and web-scraped texts.

**Results**: We created a 345,000 species file dataset with natural language descriptions of global plant species from monographs, Wikipedia and databases. It achieved over 99% coverage and high recall on New Caledonia and Cameroon Floras datasets, enabling advanced AI-based plant taxonomy analysis.

## Introduction

The taxonomic description of plants is a fundamental source of knowledge for botany, ecology, conservation and environmental sciences. Numerous international web resources provide information on plant distribution and taxonomy (e.g. Page (2013), Miller and Ulate (2017)).

In this paper, we present a new and original dataset comprising approximately 390,000 pages of natural language descriptions of plant species from around the world. The corpus contains 113 million words and includes 345,260 species-level files (corresponding to 197,000 accepted species). The sources are distributed between 35% PDF-derived content and 65% web-derived content. Each file contains an average of 290 words. The dataset occupies 1.41 GiB of disc space on Windows (1 GiB = 2³° bytes). This dataset is accompanied by a metadata file compliant with the RO-Crate specifications (Sefton et al. 2023), as well as a quality assessment report provided for each individual file and for the entire text collection. These expert-written texts include a wealth of information on morphology and life cycle — what botanists and ecologists refer to as plant traits — along with environmental context, such as habitat and geographic distribution (Kattge et al. 2020). However, this information is largely scattered across printed Floras, monographs and scientific articles and is rarely structured in a way that is directly usable by automated information systems.

Several approaches have been proposed to extract taxonomic descriptions from such texts. One method involves using large language models (LLMs) to process descriptions and extract structured information (Marcos et al. 2025). LLMs have shown promising results in extracting morphological traits and their observable characteristics from unstructured text, achieving F1-scores over 75% when automatically replicating manually-curated species-trait matrices (Marcos et al. 2025). Similarly, Nainia et al. 2024 fine-tuned CamemBERT using monographs from the Floras of New Caledonia and Cameroon.

Another approach relies on semi-automatic extraction methods, based on computational linguistics techniques. For example, morphological descriptions of plants in Spanish were processed from the books Trees of Costa Rica, Volumes III & IV from the Manual of Plants of Costa Rica (Mora-Cross and Araya 2018). These systems ingest morphological descriptions in tabular format and generate structured XML files documenting plant structures, characters and their relationships.

Additional tools also support the structuring of taxonomic content. GoldenGATE, for instance, is an XML editor that facilitates the markup of plain-text taxonomic descriptions (Cui 2008). It works on full documents, allowing users to paginate text and tag taxonomic and species names.

Furthermore, efforts have been made to integrate and visualise both legacy and prospective taxonomic literature (Miller et al. 2015). These efforts often employ biodiversity informatics standards — such as Darwin Core Archive (G.B.I.F. 2021) — to enable the aggregation and reuse of content for diverse needs. Ontologies and thesauri are frequently used for semantic annotation and interpretation (Cooper et al. 2018). Several ontologies have been developed for botany, including: the Plant Ontology (PO) (Jaiswal et al. 2006, Cooper et al. 2012), AusTraits Plant Dictionary (APD) Ontology (Wenk et al. 2024), the Phenotype And Trait Ontology (PATO) (Gkoutos et al. 2009), the Trait Ontology (TO) (Arnaud et al. 2012) and the Flora Phenotype Ontology (FLOPO) (Hoehndorf et al. 2016), which contains over 24,000 trait-value combinations.

These ontologies are widely used for document annotation (Van Auken et al. 2012, Hoehndorf et al. 2016). The extraction of taxonomic content remains an active area of research, with an increasing number of tools and approaches being developed (Cui 2008, Mora-Cross and Araya 2018, Marcos et al. 2025).

Analysis of distributions in texts has a long history (Turenne 2016) and has also been applied to plant-related datasets (Turenne and Tien 2015). The emergence of advanced AI systems, such as GPT (Brown et al. 2020), LLaMA (Touvron et al. 2023) and OpenCLIP (Ilharco et al. 2025), has significantly accelerated progress in natural language understanding, multimodal representation learning and scalable foundation model development. Recent advances in plant morphological trait identification and multimodal learning have demonstrated the potential of deep learning for herbarium image segmentation (Ariouat et al. 2025). With the advent of artificial intelligence and natural language processing (NLP), the development of clean, rich and structured textual datasets has become a critical step towards training models capable of extracting, analysing and interpreting botanical information at scale.

Main sources of botanical textual descriptions include (Agosti and Egloff 2009, Hoehndorf et al. 2016, Weigelt et al. 2019, Yang et al. 2024): scientific articles, social media and grey literature (regional and national Floras, herbarium labels).

Outputs from large language models can serve as supplementary textual sources; however, their factual accuracy often falls short compared to curated scientific articles or taxonomic descriptions found in monographs. To build our text data collection, we selected Wikipedia, botanical repositories and national Floras due to their relevance and comprehensive coverage of plant taxonomic descriptions. Challenges in textual extraction include:

a) Linguistic heterogeneity

Descriptions vary in style, vocabulary and structure depending on the author, historical period and linguistic context (e.g. Latin, French, English, Spanish, German, Russian, Chinese);

b) Specialised botanical lexicon

Taxonomic language uses a highly specific morphological vocabulary that is often polysemous or subject to synonymy. Automatic trait recognition requires specialised lexicons and normalisation tools, such as glossaries and ontologies;

c) Implicit information structure

Descriptions follow implicit conventions, such as standard morphological order (root > stem > leaf > flower > fruit), syntactic ellipses, abbreviations and unstated units (e.g. mm, cm);

d) Telegraphic language

Botanical texts are often highly compressed, omitting verbs, which complicates syntactic parsing with standard NLP tools.

While LLMs have shown promise, they still face issues related to factual accuracy and hallucination (Bang et al. 2023). Moreover, many studies on ontology acquisition and annotation are based on small, manually annotated datasets.

This highlights the need for large-scale, precise and structured datasets. We call the dataset, presented in this paper, a data text collection because it is a centralised repository of data files stored in free text natural/raw format from a heterogeneity of data sources (>550).

This data text collection addresses this gap and contributes significantly to the advancement of botanical information extraction.

## Project description

### Title

Creation of a text dataset of taxonomic descriptions

### Design description

Overview of the Dataset and Workflow

**Figure 1.**
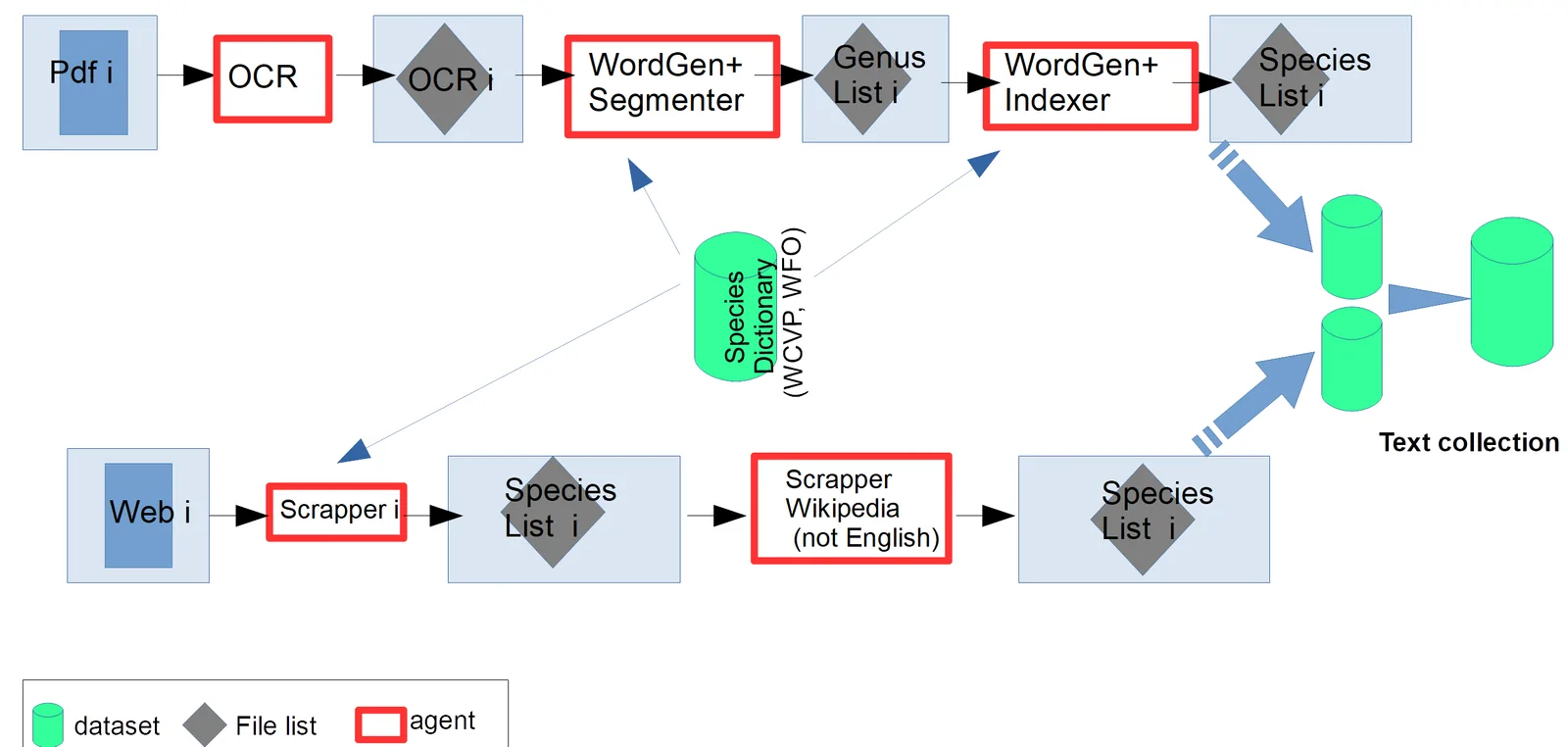
General workflow about data acquisition.

As shown in Fig. 1, the document processing pipeline consists of two main workflows: one for PDF documents (top and bottom) and one for web data (right side).

In the PDF workflow (agents are described in Turenne et al. (2026)), each PDF is processed page by page using OCR (Optical Character Recognition) to extract the text content. The individual page texts are then concatenated into a single OCR file per PDF. The resulting OCR text is segmented into family-, genus- and species-level files. botanical descriptions are hierarchical, species descriptions are often incomplete in isolation, with characters shared by all species within a genus described at the genus level and characters common to all genera within a family described at the family level.

In the web scraping workflow, we first detect web pages associated with individual species and extract the textual content. If the content is not in English, we retrieve the corresponding Wikipedia entry in the original language for the species. The result is a unified text file per species, combining both web and Wikipedia data (if it exists).

Finally, in the integration step, all processed text files — whether derived from PDFs or web sources — are consolidated into a single structured repository, organised by species.

**Data Acquisition**

**OCR-based extraction**

Over 550 monographs spanning 36 regions were analysed (Table 1). Most PDFs, published between 1845 and 2000, scanned images from botanical volumes requiring OCR. We used Tesseract OCR v.5.5.0, an open-source engine combining classical algorithms and long short-term memory neural networks, capable of handling historical and low-quality scans (Smith 2007, Tesseract-OCR-Project 2026). The model incorporates image features, optional dictionaries and frequency tables. OCR converted scanned pages into machine-readable text, enabling downstream natural language processing tasks, such as tokenisation, text extraction and analysis of botanical content.

A detailed list of sources and references is provided in the Suppl. material 1. A complete list of the PDF sources used for text analysis is provided in the Supplementary Material.

**Web scraping**

Certain websites provide high-quality information about plant species and Wikipedia is amongst the most comprehensive. As a globally recognised knowledge repository, Wikipedia allowed us to retrieve 122,000 pages, each corresponding to a distinct plant species (i.e. one page per species). This constitutes a substantial body of natural language text that can be exploited for large-scale botanical data mining. Unlike scanned PDF documents, Wikipedia pages do not require OCR processing, significantly simplifying the extraction pipeline.

Table 2 shows the copyright requirements for web sites. While Wikipedia provides a valuable, broad overview of plant species from all over the world, we deliberately differentiate our data sources by prioritiing web scraping from specialised websites that offer deeper expertise and localised precision in specific botanical areas.

Species-related information was collected only for pages with sufficient textual content. Since each website has its own HTML structure and CSS styling, a dedicated scraper was developed for each source. Due to the ethical and respectful use of web resources (to avoid overload of infrastructure), data collection was rate-limited, making the overall process time-consuming. Scraping time for all websites is 1,370 hours in total. A token is supposed to be a string with alphabetical letters (not a number or a punctuation). We tokenise using regular expressions. Tokens were created by splitting the text using a predefined list of character delimiters.

**Wikipedia**

**Figure 2.**
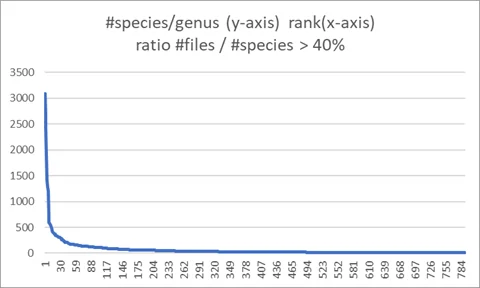
Distribution of genera in Wikipedia.

Data obtained from Wikipedia enables investigation about the popularity of plant genera by analying species-level data extracted from Wikipedia, assessing how frequently and prominently different genera are represented across the corpus (Fig. 2). It reflects contributor engagement and a potential audience, which may be further characterised using Google Trends data. We can also extract a set of genera that are over-represented in Wikipedia (Table 3). Table 3 shows the number of genera found in Wikipedia: 14,822, representing 31.9% of the total 46,427 genera.

**Species Name Recognition and Normalisation**

**Dictionaries**

To structure the dataset, each text segment was reduced to a file-species unit, with files named for specific plant species. Accurate extraction of scientific names is crucial, linking botanical knowledge sources (Lakshmi et al. 2012). Unlike Nainia et al. 2024, who used fine-tuned language models, we applied a computational linguistics approach using lexical transformations and curated dictionaries. Our algorithm, **WordGen**, merges three resources — World Checklist of Vascular Plants (WCVP-KEW 2024), World Flora Online (Miller and Ulate 2017 and TRY (Kattge et al. 2020) — providing standardised names and metadata on accepted taxa and synonyms. We adopted a reference dictionary approach, using the World Flora Online (WFO) as the primary taxonomic backbone. Entries from the World Checklist of Vascular Plants (WCVP) were compared against this reference and all names were retained in the merged dictionary together with their associated status (accepted name or synonym). This strategy allows us to preserve the richness of both sources, while maintaining traceability of taxonomic assignments. We note that discrepancies may occur between databases, including cases where a name is treated as accepted in one source and as a synonym in another. In such situations, WFO was systematically used as the primary reference, complemented by TRY and WCVP, although the biodiversity community has increasingly transitioned to utiliing the CoL/GBIF ChecklistBank, which aggregates various other resources (Berendsohn et al. 2026). We also observed that some name combinations may occur multiple times across sources and that disambiguation may require additional contextual information, such as family-level assignment. However, resolving these ambiguities was beyond the scope of the present data paper and is left for future work.

A genus groups multiple species and the standard format for scientific names follows the binomial nomenclature: Genus epithet (e.g. *Quercus pubescens*). When identifying subspecies (e.g. Quercus pubescens subsp. anatolica), they are treated as synonyms of the parent species to maintain consistency, effectively making subsp. anatolica synonymous with *Quercus pubescens*. In many botanical resources, these infraspecific names act as de facto synonyms of the accepted parent species. This means the specific distinguishing traits originally used to describe the subspecies are now incorporated into the broader concept of the accepted parent taxon. *Quercus pubescens* serves as the accepted scientific name for the downy or pubescent oak, with *Quercus humilis* recognied as a taxonomic synonym for this species.

Our dictionary contains over 1.6 million entries — including species, subspecies and official synonyms — serving as a dedicated reference for verifying taxa names compiled exclusively from trusted resources (TRY, WCVP, WFO), excluding all websites and PDFs. After normalising subspecies and synonyms to their corresponding accepted species names, we retain approximately 1.2 million unique species-level names (see Table 4). The number of recorded names is much larger than the accepted nomenclature due to the vast diversity of historical naming conventions found in historical documents, necessitating the retention of these records.

The total number of accepted plant species names remains debated across taxonomic databases, with 388,342 listed in World Flora Online (WFO) and 407,961 in the World Checklist of Vascular Plants (WCVP) (Schellenberger Costa et al. 2023).

**Figure 3.**
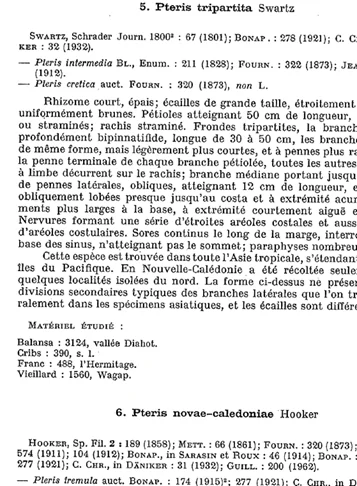
Example of text description in PDF.

Fig. 3 illustrates the distribution of botanical descriptions across textual datasets. Identifying the beginning of each species section is critical for extracting meaningful, species-specific information. However, this task is challenging due to textual inconsistencies and OCR-induced errors in digitied documents. Therefore, a robust species name recognition tool is essential.

**Construction of the Plant Description Text**

To construct the plant description text collection, a diverse set of printed and web-based resources was compiled and processed within the defined timeframe. The types and numbers of data sources included in the dataset are summarisd in Table 5.

The resulting text collection comprises multiple complementary dataset representations, including file-species and unique file-accepted species formats, providing both species-level and taxonomically consolidated records. A summary of the dataset composition is presented in Table 6.

Sections related to species in a taxonomic volume (e.g. shown here for *Pteris tripartita* and *Pteris novae-caledoniae*) correspond to distinct blocks of descriptive content.

**WordGen tool**

Accurate recognition of species names is crucial for both the OCR and indexing steps. To address this, we developed WordGen (for more details, seeTurenne et al. (2026)), a name-matching algorithm, based on transformation rules including insertion, deletion and substitution. The algorithm is designed to tolerate minor typographical or OCR errors, especially in species names. WordGen takes either a genus or a genus + epithet as input and performs approximate string matching, based on a curated botanical dictionary.

As our reference sources are dictionaries, such as World Flora Online (WFO), it is important to note that mapping authorship between the names recorded in PDFs and those listed in WFO is not straightforward and can be a significant source of error. Two illustrative examples are *Achyranthes talbotii* and *Berberis empetrifolia*:

Data from WFO:

wfo-0000516490 | urn:lsid:ipni.org:names:58932-1 | 750143 | "*Achyranthes talbotii*" | species | wfo-4000000251 | "Hutch. & Dalziel"

Data in a PDF document (Flora of Cameroon, p. 33):

2. *Achyranthes talbotii* Hutchinson & Dalziel

Mapping “Hutch. & Dalziel” to “Hutchinson & Dalziel” or “Lam.” to “Lam.-Gay” is not trivial. While the use of full binomial with author citation denominations (including author citation through normalisation using IPNI) would improve taxonomic precision, it would also significantly reduce recall, as such complete information is not consistently available across the corpus. Therefore, our approach prioritises species name matching while acknowledging this limitation.

This highlights the need not only to perform automated extraction of names, but also to evaluate the quality of these extractions. Failure to correctly map authorship may result in the omission of numerous instances.

Our corpus is geographic area-orientated, which greatly facilitates the disambiguation of species names.

**Figure 4.**
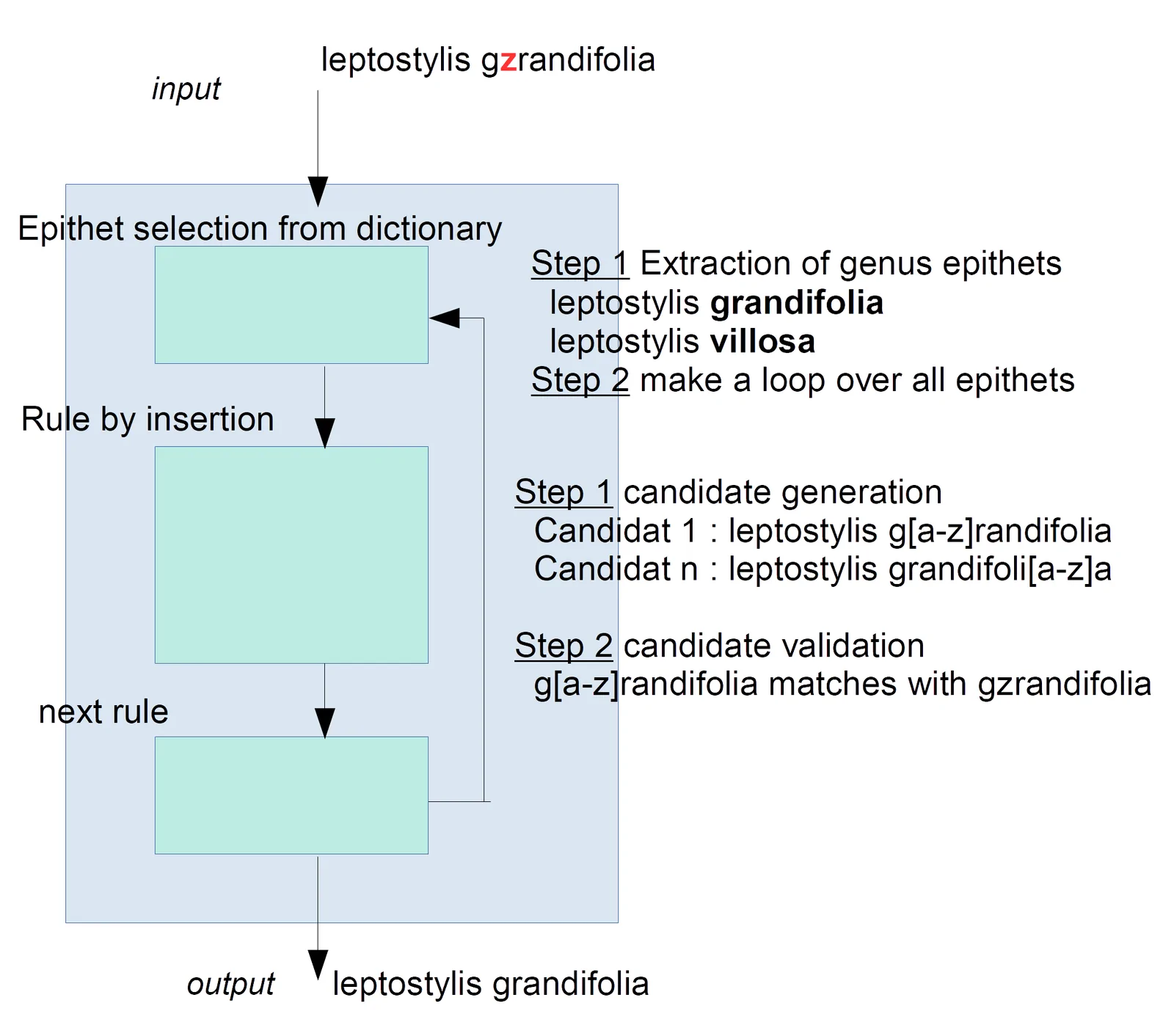
Algorithm of Wordgen.

As Fig. 4 shows, the algorithm performs fuzzy matching of species names by comparing a given input ("leptostylis gzrandidolia") against a known dictionary of epithets. This figure shows only one rule amongst several tested and applied on the input. It extracts valid species epithets associated with the genus *Leptostylis* and generates candidate corrections using insertion rules (e.g. inserting one letter at each position). The algorithm then validates candidates by checking if any match the input through a defined pattern, ultimately returning the partially corrected name "Leptostylis grandifolia".

1. Genus Input

If only a genus is provided, **WordGen** assumes that the entire input string corresponds to a genus name. For example, assuming the correct genus is Leptostylis:

- **Insertion Error**: WordGen("*leptostylisx*") → *leptostylis*
- **Deletion Error**: WordGen("*leptostyli*") → *leptostylis*
- **Substitution Error**: WordGen("*leptostylix*") → *leptostylis*

2. Genus + Epithet Input

In cases where both genus and epithet are provided (e.g. *leptostylis grandifolia*), WordGen assumes the genus is correctly spelled and performs fuzzy matching on the epithet only:

- **Insertion Error**: WordGen("*leptostylis grandifolias*") → *grandifolia*
- **Substitution Errors** (1–2 letters): WordGen("*leptostylis grandiyzlia*") → *grandifolia*
- **Deletion Erro**r: WordGen("*leptostylis grandifoli*") → *grandifolia*
- **Combined Suffix Error**: WordGen("*leptostylis grandioxbiaab*") → *grandifolia*

(Tolerance: fixed 5-character prefix and up to 7-character mismatch in suffix).

By anchoring on genus names and correcting epithet variations, WordGen plays a key role in parsing large-scale, semi-structured botanical documents.

To evaluate the quality of our technique, based on WordGen and dictionary lookup, we built an evaluation dataset using indexed species names from the “Flora of New Caledonia series”. This collection has 25 volumes. We extracted all cited species names, including those in section headers. Evaluation is explained in section "Quality control: Technical Validation".

**Normalisation**

Normalisation occurs at two levels.

First, a taxonomic normalisation links scientific names found in the text to a standard reference, such as a taxonomic dictionary. A name may refer either to an accepted taxon or to a synonym of that taxon. For example, *Leptostylis grandifolia* is a synonym of the accepted name *Pycandra grandifolia*. Thus, two documents referring to these two names actually describe the same biological entity, even though they use different terms.

Second, a string-level normalisation addresses character-level variations and typographical inconsistencies found in original texts. These errors do not involve taxonomic synonymy, but they prevent correct linking to dictionary entries. For instance, in the New Caledonia subdataset used for evaluation, the taxon *Hymenophyllum leratii* appears as *Hymenophyllum le ratii>*, where an unintended space splits the epithet. Although visually and semantically identical, the dictionary lookup would fail without correction. Another example is a pseudo-Latin declension error, where *Xylosma caledonica* is written by the author as *Xylosma caledonicum*. This variant clearly refers to the same species.

Such character-level discrepancies were manually corrected during evaluation (in the New Caledonia subdataset only) to ensure accurate normalisation. These modifications involved very small changes and were applied conservatively to preserve the integrity of the evaluation. Without such normalisation, overly strict string matching could penalise high-quality recognitions due to insignificant orthographic variations.

## Sampling methods

### Quality control

**Quality control: Technical Validation**

**Dictionary quality assessment**

Tables 7, 8, 9 present the evaluation results, based on the New Caledonia collection, which comprises 25 volumes. In this dataset, 1,236 genera and 4,818 species names were cited (Table 7 ). When these are normalised to accepted names, we identify 2,749 unique scientific names, of which 99% are present in our dictionaries (Table 7). Focusing specifically on scientific names extracted from passages describing taxonomic descriptions, we find 1,718 accepted names, with 99.82% included in the dictionaries (Table 9) (see Table 8 with synonyms).

**Automatic indexing assessment**

To evaluate the indexing performance, we compared the species names identified as cited passages in the taxonomic descriptions against the groundtruth from the New Caledonia dataset, which contains 1,718 species names. Recall was used as the evaluation metric. Our method achieved a recall of 98.89% for species names and 99.52% for genera.

Nineteen taxa (scientific names) were not identified, mainly due to name variations caused by transcription errors or transformations. Examples include:

- "11. Parsonsia catalprcarpa Baillon", which should be "Parsonsia catalpaecarpa";
- "5. Ekeocarpus yaccinioides F. Mueller ex Brongniart & Gris", which should be "*Elaeocarpus vaccinioides*".

The breakdown of missed detections (out of 19 cases) is as follows:

- 14 cases (74%) due to OCR errors;
- 2 cases (11%) due to confusion between sections and references;
- 1 case (5%) due to author writing variations;
- 1 case (5%) due to dictionary synonymy issues;
- 1 case (5%) due to entries "not in dictionary".

Table 10 presents the results of the training and test evaluation conducted on the New Caledonia dataset.

Table 11 presents the application of the algorithm for taxa and passage detection on unseen data, specifically the Cameroon dataset. This dataset includes an annotated sample of 132 genus sections and 457 taxa sections (groundtruth). The recall achieved is 93.22%, representing a realistic and reliable measure for automatic taxon section identification.

**Quality of data assessment**

We aim to assign a confidence label to each species file using linguistic and document features to assess the quality of the extracted information. Let T be a text segment extracted from an OCR and information extraction pipeline. Let W = {w_1_, w_2_, …, w_n_} be the set of alphabetic tokens extracted from T, where n = |W|. We define three linguistic features:

1. Lexical length ratio: R_SL(T) = ||w| ≤ 3| / (1 + ||w| ≥ 5|);

2. Adverb frequency: Let A(T) be the number of adverbs detected in T using a part-of-speech heuristic;

3. Punctuation density: The semi-colons ratio is defined as: R_C(T) = C(T) / n.

Each feature contributes to a partial score defined as in Table 12.

Then the global confidence score φ(T) is defined as :

φ(T) = f1(R_SL(T)) + f2(A(T)) + f3(R_C(T)) with φ(T) in the interval [0,3].

We assign the confidence label as stipulated in Table 13.

This function φ(T) defines a rule-based classifier that maps a text segment T to a confidence label {Low, Medium, High}. Applying this method to our dataset produced 19216 files with Low value (5.6% of the data text collection) (Suppl. material 2).

## Geographic coverage

### Description

**Figure 5.**
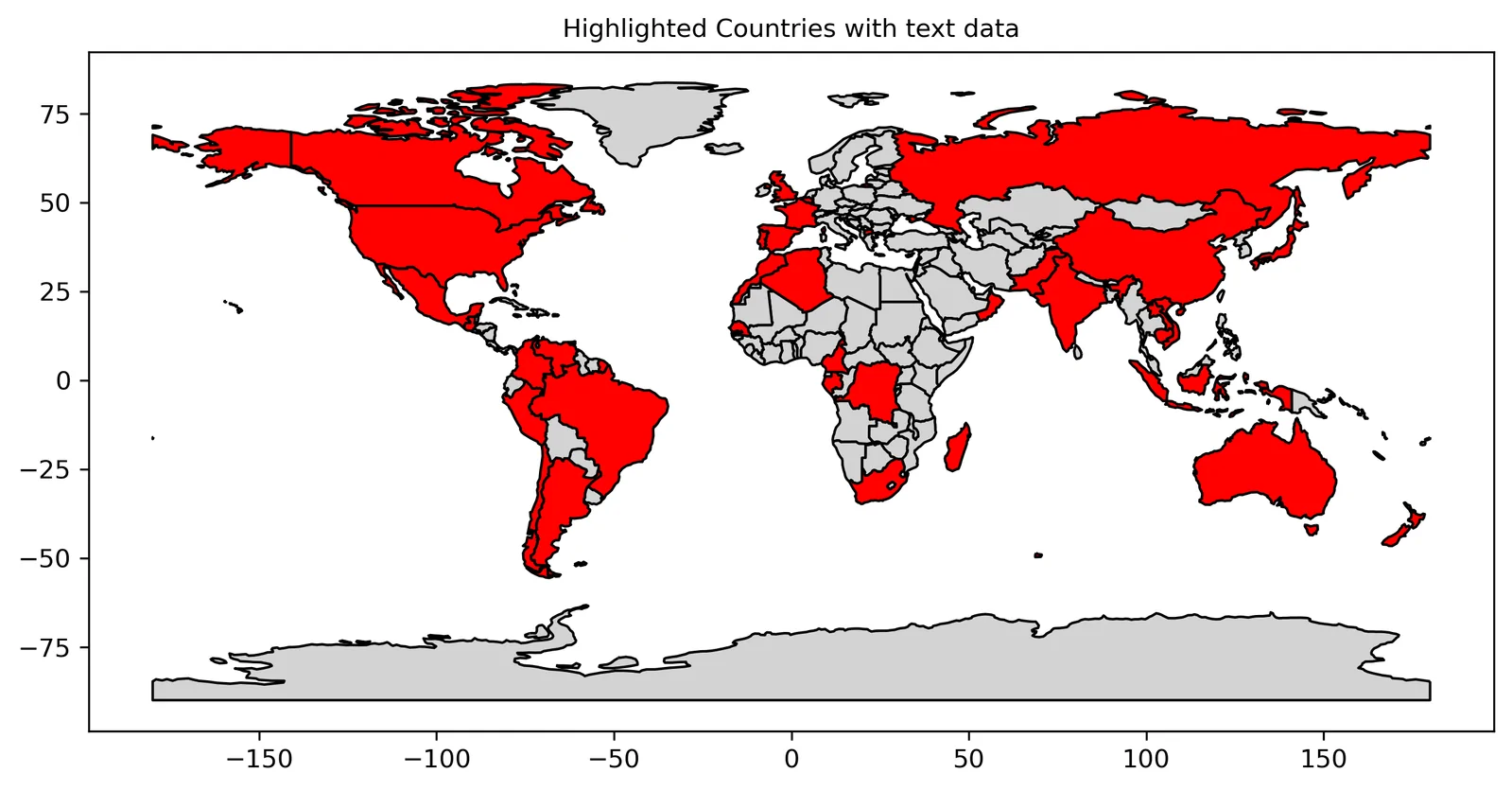
Distribution of the dataset across different geographical regions (excluding Wikipedia).

Files are organised into regional directories, each containing subdirectories for raw data (such as PDFs or scraped files), OCR-processed content (typically from monographs) and species-level files. Species files are named according to their corresponding scientific taxon names. Table 5 provides a summary of the entire dataset, while Table 6 presents the number of files it contains. Fig. 5 illustrates the geographical coverage of the dataset, excluding data from Wikipedia.

## Traits coverage

The dataset comprises taxonomic descriptions extracted from Floras (PDFs and web sources), encompassing morphological traits of flowers, fruits, stems, seeds and leaves. Traits and their associated values, as defined in FLOPO and AusTraits, can be retrieved from this text collection (see Table 14).

## Temporal coverage

### Notes

1847-1-01 - 2025-7-01

## Usage licence

### Usage licence

Creative Commons Public Domain Waiver (CC-Zero)

### IP rights notes

Creative Commons Public Domain Waiver (CC-Zero)

## Data resources

### Data package title

large text collection about world-wide Flora taxonomic and morphological descriptions

### Resource link

https://zenodo.org/records/20027402

### Number of data sets

1

### Data set 1.

#### Data set name

large text collection about world-wide Flora taxonomic and morphological descriptions

#### Data format

text, tsv, json

#### Download URL

https://zenodo.org/records/20027402 (Publicly available after acceptance)

#### Data format version

4.0

#### Description

The dataset contains **345,260 files**, each corresponding to a distinct **plant species** and is compressed into a single archive (**Flore_PdfFileSpecies.zip**). This archive is organised into directories by geographic area. Two complementary archives are also provided, containing OCR-processed text from botanical monographs and data collected from online botanical resources.

In total, three data packages are available:

- **Flore_PdfFileSpecies.zip** – species-specific files generated after OCR segmentation and extraction;
- **Flore_PdfFileOCR.zip** – OCR-processed text extracted from digitised botanical monographs;
- **Flore_WebFileSpecies.zip** – raw data collected from various botanical websites and
- a **RO CRATE json** file;
- a tabular file **ConfidenceLabel.tsv** for quality assessment.

A **temporary access link** to the dataset is available to reviewers. The **final public version** will be deposited on **Zenodo** and made accessible following acceptance of the paper.

## Additional information

All source references are in supplementary material (pdf file) (Suppl. material 1).

The method and code are accessible: https://zenodo.org/records/21455238

## Supplementary Material

30A6D503-45A5-52B4-81B7-EBD5D6E31797 10.3897/BDJ.14.e191990.suppl1 Supplementary material 1 Source Reference Data type pdf Brief description Integrating scanned literature and online sources to build a textual data lake for plant biodiversity. File: oo_1554374.pdf https://binary.pensoft.net/file/1554374 Nicolas Turenne, Eric Chenin, Youcef Sklab, Jean-Daniel Zucker

43757BEB-2496-58A3-A71D-60E81901846A 10.3897/BDJ.14.e191990.suppl2 Supplementary material 2 Quality Label Data type tsv Brief description Quality label for each species file. File: oo_1554375.tsv https://binary.pensoft.net/file/1554375 Nicolas Turenne, Eric Chenin, Youcef Sklab, Jean-Daniel Zucker

## Figures and Tables

**Table 1.** PDF OCR step and Web scraping (Areas, #tokens, 1 volume-equivalent = 229 pages).

	**Area**	**#volume-(equivalent)**	**#pages**	**#tokens**
1	Algeria	6	2,435	694,079
2	Argentina	1	343	41,869
3	Australia	3	940	392,150
4	Belgium	2	1,105	414,673
5	Cameroon	31-(362-files)	7,639	2,007,904
6	Canada	1	344	88,336
7	Caribbean Islands	1	256	54,290
8	Central America	25	8,156	2,843,492
9	Chile	6-(620-files)	2,584	690,828
10	China Yunnan	22	17,626	6,402,330
11	Colombia	1	241	72,482
12	Congo	234	12,761	3,831,299
13	France	19	8,889	3,198,049
14	French Caribbean Islands	6	1,288	391,038
15	Gabon	38	6,666	1,629,103
16	Great Britain	3	1,725	739,968
17	Guyana	1	462	127,939
18	Israel	1	930	356,693
19	Japan	1	494	148,712
20	lndia	7	5,676	2,801,438
21	Macedonia	1	98	21,924
22	Madagascar	10	515	137,799
23	Malesia	41	13,775	7,256,910
24	Mexico	1	315	89,511
25	New Caledonia	25	5,030	1,322,182
26	New Zealand	3	3,422	1,416,581
27	Oman	1	274	75,430
28	Peru	14	3,422	1,049,837
29	Reunion Island	2	735	177,950
30	Russia	1	636	394,933
31	Senegal	2-(556-files)	3,169	519,605
32	South Africa	2	532	114,013
33	Spain	21-(1,009-files)	9,542	3,516,377
34	United States	25	13,101	6,019,049
35	Venezuela	4	1,324	377,911
36	Vietnam	32	4,686	1,258,307
**subtotal**	**36**	**594**	**136,609**	**50,674,991**
1	Flora in Wikipedia	530	122,042	39,370,365
2	Flora of USA (efloras.org)	58	13,382	2,212,465
3	Flora of China (efloras.org)	142	32,575	3,467,040
4	Flora of Pakistan (efloras.org)	43	9,890	539,380
5	Flora of Russia (P!antaríum.ru)	109	25,085	7,426,438
6	Flora of Australia (ala.org.au)	61	14,058	2,691,065
7	Flora of Brazil (reflora.jbrj.gov.br)	148	33,967	6,897,633
8	Flora of Morocco (floramaroccana)	5	1,109	263,689
**subtotal**		**1,100**	**252,452**	**62,969,403**

**Table 2.** Details regarding the sources and access methods are provided in the Supplementary Data.

	**source**	**Copyright**
Worldwide	https://en.wikipedia.org/Wikipedia (English)	HTML pages under CC BY-SA 4.0
USA	http://www.efloras.org	HTML pages under CC BY
Russia	https://www.plantarium.ru	HTML pages. Text materials published on the website may be freely copied and distributed.
Pakistan	http://www.efloras.org	HTML pages under CC BY
Chile	http://www.efloras.org	PDF list under CC BY
Australia	https://profiles.ala.org.au	HTML pages under CC BY
Spain Portugal	http://floraiberica.es/	PDF files under CC BY-NC-SA 2.5 ES
Brazilia	https://reflora.jbrj.gov.br/	HTML pages under CC BY

**Table 3.** Top 20 most popular genera in Wikipedia.

**Genus**	**#species**	**#files**	**#files/#species**
* Bulbophyllum *	*3,092*	*1,915*	*0.619340233*
* Acacia *	*2,664*	*1,341*	*0.503378378*
* Quercus *	*2,405*	*1,246*	*0.518087318*
* Allium *	*1,892*	*810*	*0.428118393*
* Tillandsia *	*1,408*	*587*	*0.416903409*
* Eucalyptus *	*1,273*	*879*	*0.690494894*
* Prunus *	*1,211*	*651*	*0.537572254*
* Utricularia *	*679*	*288*	*0.424153166*
* Acer *	*599*	*392*	*0.442404*
* Grevillea *	*584*	*414*	*0.70890411*
* Melaleuca *	*556*	*308*	*0.553956835*
* Solidago *	*531*	*267*	*0.502824859*
* Aechmea *	*495*	*225*	*0.454545*
* Tabernaemontana *	445	316	0.71011236
* Hibbertia *	427	266	0.62295082
* Lilium *	407	219	0.538083538
* Caladenia *	390	313	0.802564103
* Fritillaria *	388	189	0.487113402
* Drosera *	370	203	0.548648649
* Tulipa *	369	222	0.601626

**Table 4.** Number of genera and species (merged dictionaries).

**Scientific names (including synonyms)**	**Merge (WFO+WCVP)**	**WCVP only**
Genera	46,068	34,798
Taxa (species)	1,127,959	996,093

**Table 5.** Summary of printed and web-based data sources used to construct the plant description dataset (within the processing time frame).

**Geographic areas**	**Equivalent volume**	**Time (OCR+scraping)**	**#pages**	**#tokens**
36+Wikipedia	1,694	1,497 hours (62 days)	389,061	113,644,394

**Table 6.** Dataset composition summary (file-species refers to one file per species name; unique file-accepted species refers to one file grouping all entries associated with a given accepted species name).

**#pages**	**#file-species**	**#unique file-species**	**#unique file-accepted species**
389,061	345,260	278,687	197,442
#Wikipedia pages	#web pages	#monograph pages	total
122,044	130,408	136,609	389,061

**Table 7.** Evaluation dataset using manual annotation. The first row lists all scientific names of taxa (genus + epithet) found in the whole corpus at any position. The second row includes only accepted names (with synonyms mapped to their accepted names). The third and final row shows the comparison between names found in the corpus and those present in the dictionary.

	**Genera**	**Taxa**
All scientific names	1,236	4,818
scientific names (accepted taxa)	533	2,749
scientific names (accepted taxa) not in dictionary	23 (1.86%)	48 (0.99%)
scientific names (accepted taxa) in dictionary	98.14%	99.00%

**Table 8.** Manual identification of species sections (a section is a passage describing a given species) and comparison with dictionaries. The New Caledonia dataset is manually annotated.

	**Genera**	**Taxa**
scientifc names	444 (35.92%)	1,776 (36.9%)
Not in dictionary	0	3 (0.17%)
In dictionary	100%	99.83%

**Table 9.** Same as Table 9, but using accepted names.

	**Genera**	**Taxa**
scientifc names (ground truth)	420	1,718
Not in dictionary	0	3 (0.18%)
In dictionary	100%	99.82%

**Table 10.** Automatic detection using Wordgen and dictionaries, to detect genera and taxa sections (i.e. passages) and indexing. The New Caledonia dataset manually annotated is used (testing part of evaluation) (see Table 10).

	**Genera**	**Taxa**
Groundtruth	420	1,718
Automatic extraction	651	2,787
Converted to accepted names	517	2,248
Recall	418 (99.52%)	1,699 (98.89%)

**Table 11.** Automatic genera and taxa detection (Passage) and indexing, using the manually annotated Cameroon Dataset (validation part of evaluation).

	**Genera**	**Taxa**
Ground truth	132	457
Recall	128 (96.97%)	426 (93.22%)

**Table 12.** Score conditions.

**Function**	**Condition 1**	**Condition 2**	**Condition 3**
f1(R_SL)	1 if R_SL < 0.5	0.5 if 0.5 ≤ R_SL < 1.0	0 otherwise
f2(A)	1 if A ≥ 2	0.5 if A = 1	0 if A = 0
f3(R_C)	1 if R_C < 0.05	0.5 if 0.05 ≤ R_C < 0.1	0 if R_C ≥ 0.1

**Table 13.** Label assignment.

**Label**	**Condition**
High	φ(T) ≥ 2.5
Medium	1.5 ≤ φ(T) < 2.5
Low	φ(T) < 1.5

**Table 14.** Overview of traits used to define extraction rules.

**Traits (1-14)**	**Traits (15-29)**
Stem texture	Leaflet/leaf base – acute
Presence of thorns	Leaflet/leaf base – rounded
Leaf margin	Leaflet/leaf base – auriculate
Leaflet/leaf size	Leaflet/leaf base – I don’t know
General shape of the lamina – elliptic	Leaf attachment on the stem
General shape of the lamina – oval	Leaf hairiness
General shape of the lamina – obovate	Presence of flowers
General shape of the lamina – lanceolate	Presence of fruit
General shape of the lamina – no leaf	Flowers and fruits not visible
Leaf arrangement on the stem	Inflorescence
Leaflet/leaf tip – acute	Infructescence
Leaflet/leaf tip – rounded	Neither inflorescence nor infructescence
Leaflet/leaf tip – auriculate	Simple flower
Leaflet/leaf tip – I don’t know	Simple fruit
	Leaf Compoundness
