# Supplementary material for: Integrating scanned literature and online sources to build a data text collection for plant biodiversity
Source: Biodivers Data J. 2026 Sep 24;14:e191990. doi: 10.3897/BDJ.14.e191990 (PMC13631553; doi:10.3897/BDJ.14.e191990)
Supplement: Supplementary file 1 [file bdj-14-e191990-s001.pdf]

Title

Integrating Scanned Literature and Online Sources to Build a Textual Data Lake for Plant Biodiversity

Authors

Nicolas Turenne[1,2], Eric Chenin[2], Youcef Sklab[2], Jean-Daniel Zucker[2]

Affiliations

1. INRAE, France.
2. IRD, UMMISCO, Sorbonne Université, Paris France.

corresponding author(s): Nicolas Turenne (Email: nicolas.turenne@ird.fr)

Copyright

1 Berhaut J. (1971) Flore illustrée du Sénégal. Dicotylédones, Tome 1 : Acanthacées à Avicenniacées . Publisher: Dakar : Ministère du Développement Rural [695 pages] [pdf] [fr] [Senegal]

2 Berhaut J. (1974) Flore illustrée du Sénégal. Dicotylédones, Tome 2: Balanophoracées à Composées . Publisher: Dakar : Ministère du Développement Rural [695 pages] [pdf] [fr] [Senegal]

3 Berhaut J. (1975) Flore illustrée du Sénégal. Dicotylédones, Tome 3: Connaracées à Euphorbiacées . Publisher: Dakar : Ministère du Développement Rural [634 pages] [pdf] [fr] [Senegal]

4 Berhaut J. (1975) Flore illustrée du Sénégal. Dicotylédones, Tome 4: Ficoidées à Légumineuses . Publisher: Dakar : Ministère du Développement Rural [625 pages] [pdf] [fr] [Senegal]

Open Access

1 Online Flora (world wide) from WIKIPEDIA. (2025, 24 june). Wikipedia. https://en.wikipedia.org/wiki/Main_Page [en]

2 Online Flora of North-America (2025, 24 june) http://www.efloras.org/flora_page.aspx?flora_id=1 . [en] [USA] [Canada]

3 Online Flora of China (2025, 24 june) http://www.efloras.org/flora_page.aspx?flora_id=2. [en] [China]

4 Online Flora of Pakistan (2025, 24 june) http://www.efloras.org/flora_page.aspx?flora_id=5. [en] [Pakistan]

5 Online Flora of Russia (2025, 24 june) https://www.plantarium.ru/ Plantarium. Plants and lichens of Russia and neighboring countries: open online galleries and plant identification guide. 2007—2025. URL: https://www.plantarium.ru/lang/en.html . [ru] [Russia]

6 Online Flora of Chile (2025, 24 june) http://www.efloras.org/flora_page.aspx?flora_id=60. [es] [Chile]

7 Online Flora of Marocco (2025, 24 june) https://www.floramaroccana.fr [fr] [Marocco]

8 Online Flora of Australia (2025, 24 june) https://profiles.ala.org.au/ [en] [Australia]

9 Online Flora of Brazil (2025, 24 june) https://reflora.jbrj.gov.br/consulta/#CondicaoTaxonCP Flora e Funga do Brasil. Jardim Botânico do Rio de Janeiro. Disponível em: < http://floradobrasil.jbrj.gov.br/ > [pt] [en] [Brazil]

10 Online Flora of Iberica (2025, 24 june) http://www.floraiberica.es/ ; Castroviejo, S. (general editor). 1986–2012. Flora iberica 1–8, 10–15, 17–18, 21. Royal Botanical Garden, CSIC, Madrid [es] [Portugal] [Spain]]

11 Benoist R. and Eichhorn A. (1931) Les bois de la Guyane française. Publisher: Archives de Botanique Tome V , mémoires n°1 et 2, Caen [462 pages] [pdf] [en] [FrenchGuyana]

12 Marloth, Rudolf. The Flora of South Africa. Vol. I. Thallophyta. Bryophyta, Pteridophyta. Gymnospermae. Dicotyledones Cambridge: Cambridge University Press, 1913. [444 pages] [pdf] [en] [South-Africa]

13 Klopper RR, Crouch NR, Smith GF, Van Wyk AE (2020) A synoptic review of the aloes (Asphodelaceae, Alooideae) of KwaZulu-Natal, an ecologically diverse province in eastern South Africa. PhytoKeys 142: 1–88. https://doi.org/10.3897/phytokeys.142.48365 [88 pages] [pdf] [en] [South-Africa]

14 Battandier, J.-A., & Trabut, L., Flore de l'Algérie, Dicotylédones, 1er fascicule Thalamiflores, Alger, Typographie Adolphe Jourdan, Paris, Librairie F.Savy 1888 [898 pages][pdf] [fr] [Algeria]

15 Gubb, Alfred S. La Flore Algérienne Naturelle et Acquise. Alger : Adolphe Jourdan, Éditeur, Place du Gouvernement, 1913. [318 pages][pdf] [fr] [Algeria]

16 Battandier, J.-A., & Trabut, L. (1902). Flore analytique et synoptique de l'Algérie et de la Tunisie. Alger: Imprimerie et Librairie Générale. [472 pages][pdf] [fr] [Algeria]

17 Munby, G. (1847). Flore de l'Algérie ou Catalogue des plantes indigènes du Royaume d'Alger, accompagné des descriptions de quelques espèces nouvelles ou peu connues. Paris : J.-B. Baillière. [166 pages][pdf] [fr] [Algeria]

18 Debeaux M.O. (1894) Flore de la Kabylie du Djurdjura , Catalogue méthodique et raisonné de toutes les plantes vasculaires et spontanées observées jusqu'à ce jour dans cette contrée, Paris, Librairie des sciences naturelles Paul Klincksieck. [484 pages][pdf] [fr] [Algeria]

19 Battandier, J.-A., (1910) Flore de l'Algérie. Supplément aux Phanérogrames. Paris Librairie des sciences naturelles Paul Klincksieck, Editeur. [97 pages][pdf] [fr] [Algeria]

20 Allen, Bruce (and Ronald A. Purcell, Robert R. Ireland) (2010) Moss Flora of Central America Part 3 Anomodontaceae - Symphyodontaceae, Missouri Botanical Garden. ISSN 0161-1542 [747 pages][pdf] [en] [Central America]

21 Allen Paul.H. and Williams Louis O. (1980) Flora of Panama, Orchids of Panama. Missouri Botanical Garden. ISSN 06161-1542 [518 pages][pdf] [en] [Central America]

22 Nash , Dorothy L. and Williams Louis O. (1976) Flora of Guatemala, Fieldiana: Botany Volume 24 Part XII published by Field Museum of Natural History, ISSN 0015-0746 [636 pages][pdf] [en] [Central America]

23 Standley Paul C. and Record Samuel J. (1936) The Forests and Flora of British Honduras, published by Field Museum of Natural History, Botanical Series volume XII, [462 pages][pdf] [en] [Central America]

24 Standley Paul C. and Williams Louis O. (1962) Flora of Guatemala, Fieldiana: Botany

Volume 24 Part VII number 2 published by Field Museum of Natural History, [124 pages][pdf] [en] [Central America]

25 Standley Paul C. and Williams Louis O. (1967) Flora of Guatemala, Fieldiana: Botany Volume 24 Part VIII number 3 published by Field Museum of Natural History, [80 pages][pdf] [en] [Central America]

25 Standley Paul C. and Williams Louis O. (1963) Flora of Guatemala, Fieldiana: Botany Volume 24 Part VII number 4 published by Field Museum of Natural History, [192 pages][pdf] [en] [Central America]

26 Standley Paul C. and Williams Louis O. (1961) Flora of Guatemala, Fieldiana: Botany Volume 24 Part VII number 1 published by Field Museum of Natural History, [216 pages][pdf] [en] [Central America]

27 Mc Vaugh Rogers (1961) Flora of Guatemala, Fieldiana: Botany Volume 24 Part VII number 3 published by Field Museum of Natural History, [152 pages][pdf] [en] [Central America]

28 Nash , Dorothy L. and Dieterle Jennie V.A. (1976) Flora of Guatemala, Fieldiana: Botany Volume 24 Part XI number 4. Fieldiana: Botany Volume 24 Part XI, ISSN 0015-0746 [186 pages][pdf] [en] [Central America]

29 Standley Paul C., Williams Louis O. and Gibson Dorothy N.(1969) Flora of Guatemala, Fieldiana: Botany Volume 24 Part VIII number 4. Fieldiana: Botany Volume 24 Part VIII, ISSN 0015-0746 [240 pages][pdf] [en] [Central America]

30 Standley Paul C., Steyermark Julian A.(1952) Flora of Guatemala, Fieldiana: Botany Volume 24 Part III Fieldiana: Botany Volume 24 Part III, Chicago Natural History Museum [454 pages][pdf] [en] [Central America]

31 Standley Paul C., Steyermark Julian A.(1949) Flora of Guatemala, Fieldiana: Botany Volume 24 Part VI Fieldiana: Botany Volume 24 Part VI, Chicago Natural History Museum [462 pages][pdf] [en] [Central America]

32 Standley Paul C., Steyermark Julian A.(1946) Flora of Guatemala, Fieldiana: Botany Volume 24 Part IV Fieldiana: Botany Volume 24 Part IV, Chicago Natural History Museum [516 pages][pdf] [en] [Central America]

33 Swallen Jason R. and McClure F.A. (1955) Flora of Guatemala, Grasses of Guatemala. Fieldiana: Botany Volume 24 Part II Fieldiana: Botany Volume 24 Part II, Chicago Natural History Museum [416 pages][pdf] [en] [Central America]

34 Standley Paul C., and Williams Louis O. (1975) Flora of Guatemala. Fieldiana: Botany Volume 24 Part XI numbers 1 to 3 Fieldiana: Botany Volume 24 Part XI, Chicago Natural History Museum [304 pages][pdf] [en] [Central America]

35 Standley Paul C., Williams Louis O. and Gibson Dorothy N. (1970-1973) Flora of Guatemala, Fieldiana: Botany Volume 24 Part IX numbers 1 to 4 Fieldiana: Botany Volume 24 Part IX, Chicago Natural History Museum [264 pages][pdf] [en] [Central America]

36 Standley Paul C., Williams Louis O. and Gibson Dorothy N. (1974) Flora of Guatemala, Fieldiana: Botany Volume 24 Part X numbers 3 and 4 Fieldiana: Botany Volume 24 Part X, Chicago Natural History Museum [340 pages][pdf] [en] [Central America]

37 Standley Paul C., Williams Louis O. and Gibson Dorothy N. (1973) Flora of Guatemala, Fieldiana: Botany Volume 24 Part IX numbers 3 and 4 Fieldiana: Botany Volume 24 Part IX, Chicago Natural History Museum [218 pages][pdf] [en] [Central America]

38 Gentry Johnnie L. and Standley Paul C. (1974) Flora of Guatemala, Fieldiana: Botany Volume 24 Part X numbers 1 and 2 Fieldiana: Botany Volume 24 Part X, Chicago Natural History Museum [178 pages][pdf] [en] [Central America]

39 Williams Terua P. (1977) Comprehensive Index to the Flora of Guatemala, Fieldiana: Botany Volume 24 Part XIII. Fieldiana: Botany Volume 24 Part XIII, Chicago Natural History Museum [296 pages][pdf] [en] [Central America]

40 Standley Paul C., Steyermark Julian A. (1946) Flora of Guatemala, Fieldiana: Botany Volume 24 Part V. Fieldiana: Botany Volume 24 Part V. Chicago Natural History Museum [522 pages][pdf] [en] [Central America]

41 Standley Paul C., Steyermark Julian A. (1958) Flora of Guatemala, Fieldiana: Botany Volume 24 Part I. Fieldiana: Botany Volume 24 Part I. Chicago Natural History Museum [504 pages][pdf] [en] [Central America]

42 Standley Paul C., Williams Louis O. and Lundell Cyrus L. (1966) Flora of Guatemala, Myrsinaceae. Fieldiana: Botany Volume 24 Part VIII numbers 1 and 2. Fieldiana: Botany Volume 24 Part VIII. Chicago Natural History Museum [240 pages][pdf] [en] [Central America]

43 Nash Dorothy L. and Williams Louis O. (1976) Flora of Guatemala. Fieldiana: Botany Volume 24 Part XII. Fieldiana: Botany Volume 24 Part XII. Chicago Natural History Museum [636 pages][pdf][en][Central America]

44 Heckel E. (1896) Flore phanérogamique des Antilles françaises (Guadeloupe, Martinique). Annales de l'institut colonial de Marseille. 3ième volume. [696 pages][pdf] [fr] [French Antilles]

45 Mazé H (1896) Contribution à la flore de La Guadeloupe. Basse-Terre imprimerie du gouvernement [210 pages][pdf] [fr] [French Antilles]

46 Duss Le R.-P. (1903) Division, Nomenclature & Habitat des Fougères & Lycopodes des Antilles Françaises [130 pages][pdf] [fr] [French Antilles]

47 Duss Le R.-P. (1903) Enumération méthodique des Muscinées des Antilles Françaises - Hépatiques [130 pages][pdf] [fr] [French Antilles]

48 Bescherelle Emile (année ?) Florule Bryologique des Antilles Françaises [108 pages][pdf] [fr] [French Antilles]

49 Bescherelle Emile (1871) Enumération des Glumacées récoltées aux Antilles Françaises [44 pages][pdf] [fr] [French Antilles]

50 Barthélémy Daniel, Brion Cecilia, Puntieri (2015) Guide illustré de la flore de Patagonie IRD Editions, ISBN 978-2-7099-1874-9 [343 pages][pdf] [fr] [Argentina]

51 Stanley T.D. and Ross E.M. (1983) Flora of South-eastern Queensland, volume 1, Queensland Herbarium, Queensland Department of Primary Industries. Queensland Government. ISBN 074217606 [568 pages][pdf] [en] [Australia]

52 Black J.M. and Rogers R.S. (1922) Flora of South Australia Part I. Cyatheaceae-Orchidaceae. Adelaide: printed by R.E.E. Rogers Government Printer, North Terrace. [168 pages][pdf] [en] [Australia]

53 Black J.M. (1909) The Naturalised Flora of South Australia. Adelaide: printed by J.M. Black Hussey & Gillingham, General Printers. [204 pages][pdf] [en] [Australia]

54 De Vos André (1885) Flore complète de la Belgique. Espèces indigènes et plantes cultivées sans abri. Mons, Hector Manceaux, Imprimeur-Editeur [776 pages][pdf] [fr] [Belgique]

55 Crépin François (1860) Flore de Belgique ou Description des familles et des genres accompagnée de Tableaux Analytiques destinés à faire parvenir aisément aux noms des espèces suivis du catalogue raisonné des plantes qui croissent spntanément en Belgique, et de celles qui y sont généralement cultivées. [329 pages][pdf] [fr] [Belgique]

56 Letouzey René (1963) Flore du Cameroun volume 1. Rutaceae, Zygophyllaceae, Balanitaceae. Muséum National d'Histoire Naturelle éditions. [179 pages][pdf] [fr] [Belgique]

57 Letouzey René (1963) Flore du Cameroun volume 1. Rutaceae, Zygophyllaceae, Balanitaceae. Muséum National d'Histoire Naturelle éditions. [179 pages][pdf] [fr] [Cameroun]

58 Aubréville André. (1964) Flore du Cameroun volume 2. Sapotaceae . Muséum National d'Histoire Naturelle éditions. [149 pages][pdf] [fr] [Cameroun]

59 Tardieu-Blot M.-L. (1964) Flore du Cameroun volume 3. Pteridophytae. Muséum National d'Histoire Naturelle éditions. [377 pages][pdf] [fr] [Cameroun]

60 Koechlin J. (1965) Flore du Cameroun volume 4. Scitamineae (Musaceae, Strelitziaceae, Zingiberaceae, Cannaceae, Marantaceae). Muséum National d'Histoire Naturelle éditions. [167 pages][pdf] [fr] [Cameroun]

61 Aymonin G. G. & Raynal A. (1966) Flore du Cameroun volume 5. Thymeleaceae – Onagraceae, Halorrhagaceae. [149 pages][pdf] [fr] [Cameroun]

62 Keraudren-Aymonin M. (1967) Flore du Cameroun volume 6. Cucurbitaceae. Muséum National d'Histoire Naturelle éditions [197 pages][pdf] [fr] [Cameroun]

63 Letouzey René (1968) Flore du Cameroun volume 7. Les botanistes au Cameroun. Muséum National d'Histoire Naturelle éditions [115 pages][pdf] [fr] [Cameroun]

64 Letouzey René (1968) Flore du Cameroun volume 8. Ulmaceae, Urticaceae. Muséum National d'Histoire Naturelle éditions [227 pages][pdf] [fr] [Cameroun]

65 Aubréville André. (1970) Flore du Cameroun volume 9. Leguminosae–Cesalpinioideae . Muséum National d'Histoire Naturelle éditions [345 pages][pdf] [fr] [Cameroun]

66 Jacques-Félix H. (1970) Flore du Cameroun volume 10. Ombellae (Ombelliferae, Araliaceae). Muséum National d'Histoire Naturelle éditions [113 pages][pdf] [fr] [Cameroun]

67 Letouzey René & White Frank (1970) Flore du Cameroun volume 11. Ebenaceae – Ericaceae . Muséum National d'Histoire Naturelle éditions [213 pages][pdf] [fr] [Cameroun]

68 Leeuwenberg A. J. M. (1972) Flore du Cameroun volume 12. Loganiaceae. Muséum National d'Histoire Naturelle éditions [161 pages][pdf] [fr] [Cameroun]

69 Descoings B. (1973) Flore du Cameroun volume 13. Vitaceae, Leeaceae. Muséum National d'Histoire Naturelle éditions [147 pages][pdf] [fr] [Cameroun]

70 Badré F. & Lawalrée A. (1972) Flore du Cameroun volume 14. Malpighiaceae, Linaceae, Lepidobotryaceae, Ctenolophonaceae, Humiriaceae, Erythroxylaceae, Ixonanthaceae, Santalaceae . Muséum National d'Histoire Naturelle éditions [79 pages][pdf] [fr] [Cameroun]

71 Villiers Jean-François (1973) Flore du Cameroun volume 15. Icacinaceae, Olacaceae, Pentadiplandraceae, Opiliaceae, Octoknemaceae. Muséum National d'Histoire Naturelle éditions [205 pages][pdf] [fr] [Cameroun]

72 Fouilloy R. & Hallé N. (1973) Flore du Cameroun volume 16. Sapindaceae . Muséum

National d'Histoire Naturelle éditions [209 pages][pdf] [fr] [Cameroun]
73 Cavaco A. (1974) Flore du Cameroun volume 17. Amaranthaceae . Muséum National d'Histoire Naturelle éditions [71 pages][pdf] [fr] [Cameroun]
74 Aubréville André & Leroy Jean-F. (1974) Flore du Cameroun volume 18. Lauraceae, Myristicaceae, Monimiaceae . Muséum National d'Histoire Naturelle éditions [125 pages][pdf] [fr] [Cameroun]
75 Villiers Jean-François & Aubréville André (1975) Flore du Cameroun volume 19. Celastraceae, Aquifoliaceae, Salvadoraceae . Muséum National d'Histoire Naturelle éditions [111 pages][pdf] [fr] [Cameroun]
76 Letouzey René, White Frank & Aubréville André (1978) Flore du Cameroun volume 20. Chrysobalanaceae, Scytopetalaceae, Rosaceae. Muséum National d'Histoire Naturelle éditions [255 pages][pdf] [fr] [Cameroun]
77 Couvreur Thomas & Sunderland T.C.H. (1975) Flore du Cameroun volume 44. Arecaceae – Palmae. ISSN 0071-5875 Ministère de la Recherche Scientifique et de l'Innovation (MINRESI), Yaoundé, Cameroun [140 pages][pdf] [fr] [Cameroun]
78 Couvreur TLP, Dagallier L-PMJ, Crozier F, Ghogue J-P, Hoekstra PH, Kamdem NG, Johnson DM, Murray NA, Sonké B. (2022) Flore du Cameroun volume 45. Annonaceae. PhytoKeys 207: 1–532. https://doi.org/10.3897/phytokeys.207.61432 [532 pages][pdf] [en] [Cameroun]
79 Van Der Zon, Antonius Petrus Maria (1992) Graminées du Cameroun, Wageningen Agricultural University, https://edepot.wur.nl/165452 Vol. I https://edepot.wur.nl/166175 [87 pages][pdf][Cameroun]
80 Van Der Zon, Antonius Petrus Maria (1992) Graminées du Cameroun, Wageningen Agricultural University, https://edepot.wur.nl/165452 Vol. II https://edepot.wur.nl/166175 [563 pages][pdf][Cameroun]
81 Burtt B.L. and AH Gentry (1984) Flore du Cameroun volume 27. GESNERIACEAE Gesneriacees, Bignoniacees [68 pages][pdf] [fr] [Cameroun]
82 Symoens J-J and J-F Villiers (1984) Flore du Cameroun volume 26. Alismatidées, Flagellariacées . Ministère de l'enseignement supérieur et de la recherche scientifique (Mesres), Yaoundé. [84 pages][pdf] [fr] [Cameroun]
83 Liben L. (1983) Flore du Cameroun volume 25. COMBRETACEAE Combretacees . Publisher, Muséum Nat. d'Hist. Naturelle, [98 pages][pdf] [fr] [Cameroun]
84 Jacques-Felix H. (1983) Flore du Cameroun volume 24. MELASTOMATACEE Melastomatacees, Muséum national d'Histoire naturelle, [168 pages][pdf] [fr] [Cameroun]
85 Balle S. (1982) Flore du Cameroun volume 23. LORANTHACEAE Loranthacees BALLE, (Eds. B. Satabié et J. F. Leroy). D.G.R.S.T., Yaoundé, [82 pages][pdf] [fr] [Cameroun]
86 Grey-Wilson C. & Lewis J. (1981) Flore du Cameroun volume 22. BALSAMINACEAE Balsaminacées, Xyridacées [58 pages][pdf][Cameroun]
87 Jonsell Bengt, Poppendieck Hans-Helmut & Lawalrée André (1980) Flore du Cameroun volume 21.- CRUCIFERAE Crucifères, Cochlospermacées , Dipsacées . Muséum National d'Histoire Naturelle Editions [40 pages][pdf][fr][Cameroun]
88 Spotton H.B. (1897) The commonly occurring Wild Plants of Canada. A flora for the use of beginners. Publisher: W.J. Gage & Company, Limited, Toronto [344 pages][pdf] [en]

[Canada]
89 Howard Richard A. (1974) Flora of the Lesser Antilles, Orchidaceae. publisher: Arnold Arboretum, Harvard University, Jamaica Plain, Massachusetts [256 pages][pdf][en] [Caribbean]
90 云南省格物研究所编着 (1977)《云南植物志》第一卷 Flora Yunnanica , tomus 1, Spermatophyta , 科学出版社出版 [888 pages][pdf] [ch] [China]
91 云南省格物研究所编着 (1979)《云南植物志》第二卷 Flora Yunnanica , tomus 2, Spermatophyta , 科学出版社出版 [902 pages][pdf] [ch] [China]
92 中国科学院昆明植物研究所 编著 (1983)《云南植物志》第三卷 Flora Yunnanica , tomus 3, Spermatophyta , 科学出版社出版 [808 pages][pdf] [ch] [China]
93 中国科学院昆明植物研究所 编著 (1983)《云南植物志》第四卷 Flora Yunnanica , tomus 4, Spermatophyta , 科学出版社出版 [840 pages][pdf] [ch] [China]
94 中国科学院昆明植物研究所 编著 (1991)《云南植物志》第五卷 Flora Yunnanica , tomus 5, Spermatophyta , 科学出版社出版 [828 pages][pdf] [ch] [China]
95 中国科学院昆明植物研究所 编著 (1995)《云南植物志》第六卷 Flora Yunnanica , tomus 6, Spermatophyta , 科学出版社出版 [924 pages][pdf] [ch] [China]
96 中国科学院昆明植物研究所 编著 (1997)《云南植物志》第七卷 Flora Yunnanica , tomus 7, Spermatophyta , 科学出版社出版 [904 pages][pdf] [ch] [China]
97 中国科学院昆明植物研究所 编著 (1997)《云南植物志》第八卷 Flora Yunnanica , tomus 8, Spermatophyta , 科学出版社出版 [800 pages][pdf] [ch] [China]
98 中国科学院昆明植物研究所 编著 (2003)《云南植物志》第九卷 Flora Yunnanica , tomus 9, Spermatophyta , 科学出版社出版 [828 pages][pdf] [ch] [China]
99 中国科学院昆明植物研究所 编著 (2006)《云南植物志》第十卷 Flora Yunnanica , tomus 10, Spermatophyta , 科学出版社出版 [952 pages][pdf] [ch] [China]
100 中国科学院昆明植物研究所 编著 (2000)《云南植物志》第十一卷 Flora Yunnanica , tomus 11, Spermatophyta , 科学出版社出版 [776 pages][pdf] [ch] [China]
101 中国科学院昆明植物研究所 编著 (2006)《云南植物志》第十二卷 Flora Yunnanica , tomus 12, Spermatophyta , 科学出版社出版 [908 pages][pdf] [ch] [China]
102 中国科学院昆明植物研究所 编著 (2004)《云南植物志》第十三卷 Flora Yunnanica , tomus 13, Spermatophyta , 科学出版社出版 [944 pages][pdf] [ch] [China]
103 中国科学院昆明植物研究所 编著 (2003)《云南植物志》第十四卷 Flora Yunnanica , tomus 14, Spermatophyta , 科学出版社出版 [908 pages][pdf] [ch] [China]
104 中国科学院昆明植物研究所 编著 (2003)《云南植物志》第十五卷 Flora Yunnanica , tomus 15, Spermatophyta , 科学出版社出版 [900 pages][pdf] [ch] [China]
105 中国科学院昆明植物研究所 编著 (2006)《云南植物志》第十六卷 Flora Yunnanica , tomus 16, Spermatophyta , 科学出版社出版 [828 pages][pdf] [ch] [China]
106 中国科学院昆明植物研究所 编著 (2000)《云南植物志》第十七卷 Flora Yunnanica , tomus 17, Spermatophyta , 科学出版社出版 [672 pages][pdf][ch][China]
107 中国科学院昆明植物研究所 编著 (2002)《云南植物志》第十八卷 Flora Yunnanica , tomus 18, Spermatophyta , 科学出版社出版 [544 pages][pdf][ch][China]
108 中国科学院昆明植物研究所 编著 (2005)《云南植物志》第十九卷 Flora Yunnanica , tomus 19, Spermatophyta , 科学出版社出版 [704 pages][pdf][ch][China]
109 中国科学院昆明植物研究所 编著 (2006)《云南植物志》第二十卷 Flora Yunnanica , tomus 20, Spermatophyta , 科学出版社出版 [808 pages][pdf][ch][China]

110 中国科学院昆明植物研究所 编著 (2005)《云南植物志》第二十一卷 Flora Yunnanica , tomus 21, Spermatophyta , 科学出版社出版 [500 pages][pdf][ch][China]

111 Debeaux M.O. (1879) Contributions à la flore de la Chine comprenant les algues marines récoltées en Chine et les florules de ShangHai, TcheFou et TsienTsin, Fascicules I-IV, . Publisher: F. Savy, Libraire-Editeur, Paris [396 pages][pdf][fr][China]

112 Fernandez Alonso, José Luis (1995) Flora de Colombia, MONOGRAFíA N.Q 16, SCROPHULARIACEAE-ARAGOEAE, publiher: Instituto de Ciencias Naturales - Universidad Nacional de Colombia, ISSN 0120-4351 [241 pages][pdf][sp][Colombia]

113 Fisher E. (2020) Flore d'Afrique Centrale (République démocratique du Congo - Rwanda - Burundi) nouvelle série, Spermatophyta, Papaveraceae, publisher: Jardin Botanique de Meise, ISSN 0779-116X [26 pages][pdf][fr][Congo]

114 Larridon I., Thery P., Reynders M. (2022). Cyperaceae, Tribu VII. Schoeneae, VIII. Rhynchosporeae et IX. Cariceae. Dans : Sosef M.S.M. (éd.) Flore d'Afrique centrale (République démocratique du Congo - Rwanda - Burundi), nouvelle série, Spermatophyta. Meise, Jardin botanique de Meise. [version PDF, 10.5281/zenodo.14697701] [101 pages][pdf][fr][Congo]

115 Larridon I. (2022). Cyperaceae, Tribu II. Trilepideae, III. Cladieae et IV. Carpheae. Dans : Sosef M.S.M. (éd.) Flore d'Afrique centrale (République démocratique du Congo - Rwanda - Burundi), nouvelle série, Spermatophyta. Meise, Jardin botanique de Meise. [version PDF, 10.5281/zenodo.14697880] [29 pages][pdf][fr][Congo]

116 Meerts P. (2022). Labiatae, Sous-familles IV. Scutellarioideae et V. Lamioideae. Dans : Sosef M.S.M. (éd.) Flore d'Afrique centrale (République démocratique du Congo - Rwanda - Burundi), nouvelle série, Spermatophyta. Meise, Jardin botanique de Meise. [version PDF, 10.5281/zenodo.14698342] [109 pages][pdf][fr][Congo]

117 Ntore S., Lachenaud O. (2022). Rubiaceae, Tribu XIII. Hymenodictyeae, XIV. Naucleeae et XV. Cinchoneae. Dans : Sosef M.S.M. (éd.) Flore d'Afrique centrale (République démocratique du Congo - Rwanda - Burundi), nouvelle série, Spermatophyta. Meise, Jardin botanique de Meise. [version PDF, 10.5281/zenodo.14699033] [109 pages][pdf][fr][Congo]

118 Robbrecht E. (2022). Rubiaceae, Tribu XXVI. Anthospermeae et XXVII. Rubieae. Dans : Sosef M.S.M. (éd.) Flore d'Afrique centrale (République démocratique du Congo - Rwanda - Burundi), nouvelle série, Spermatophyta. Meise, Jardin botanique de Meise. [version PDF, 10.5281/zenodo.14699106] [53 pages][pdf][fr][Congo]

119 Robyns W. (1948) Cycadaceae. In : Boutique R. (éd.) Flore du Congo Belge et du Ruanda-Urundi, Spermatophytes, vol. I : 1–4. Bruxelles, I.N.E.A.C. [version PDF, 10.5281/zenodo.4247267] [12 pages][pdf][fr][Congo]

120 Robyns W. (1948) Podocarpaceae. In : Boutique R. (éd.) Flore du Congo Belge et du Ruanda-Urundi, Spermatophytes, vol. I : 5–8. Bruxelles, I.N.E.A.C. [version PDF, 10.5281/zenodo.4249299] [8 pages][pdf][fr][Congo]

121 Robyns W. (1948) Cupressaceae. In : Boutique R. (éd.) Flore du Congo Belge et du Ruanda-Urundi, Spermatophytes, vol. I : 9–10. Bruxelles, I.N.E.A.C. [version PDF, 10.5281/zenodo.4249372] [6 pages][pdf][fr][Congo]

122 Robyns W. (1948) Gnetaceae. In : Boutique R. (éd.) Flore du Congo Belge et du Ruanda-Urundi, Spermatophytes, vol. I : 11–13. Bruxelles, I.N.E.A.C. [version PDF,

10.5281/zenodo.4249768] [7 pages][pdf][fr][Congo]
123 Wilczek R. (1948) Casuarinaceae. In : Boutique R. (éd.) Flore du Congo Belge et du Ruanda-Urundi, Spermatophytes, vol. I : 14. Bruxelles, I.N.E.A.C. [version PDF, 10.5281/zenodo.4249803] [5 pages][pdf][fr][Congo]
124 Balle S. (1948) Piperaceae. In : Boutique R. (éd.) Flore du Congo Belge et du Ruanda-Urundi, Spermatophytes, vol. I : 15–27. Bruxelles, I.N.E.A.C. [version PDF, 10.5281/zenodo.4250003] [18 pages][pdf][fr][Congo]
125 Hauman L. (1948) Hydrostachyaceae. In : Boutique R. (éd.) Flore du Congo Belge et du Ruanda-Urundi, Spermatophytes, vol. I : 28–32. Bruxelles, I.N.E.A.C. [version PDF, 10.5281/zenodo.4266162] [9 pages][pdf][fr][Congo]
126 Boutique R. (1948) Myricaceae. In : Boutique R. (éd.) Flore du Congo Belge et du Ruanda-Urundi, Spermatophytes, vol. I : 33–38. Bruxelles, I.N.E.A.C. [version PDF, 10.5281/zenodo.4266178] [10 pages][pdf][fr][Congo]
127 Hauman L. (1948) Ulmaceae. In : Boutique R. (éd.) Flore du Congo Belge et du Ruanda-Urundi, Spermatophytes, vol. I : 39–51. Bruxelles, I.N.E.A.C. [version PDF, 10.5281/zenodo.4266184] [17 pages][pdf][fr][Congo]
128 Hauman L., Lebrun J. & Boutique R. (1948) Moraceae. In : Boutique R. (éd.) Flore du Congo Belge et du Ruanda-Urundi, Spermatophytes, vol. I : 52–175. Bruxelles, I.N.E.A.C. [version PDF, 10.5281/zenodo.4266191] [133 pages][pdf][fr][Congo]
129 Hauman L. (1948) Cannabaceae. In : Boutique R. (éd.) Flore du Congo Belge et du Ruanda-Urundi, Spermatophytes, vol. I : 176. Bruxelles, I.N.E.A.C. [version PDF, 10.5281/zenodo.4266258] [5 pages][pdf][fr][Congo]
130 Hauman L. (1948) Urticaceae. In : Boutique R. (éd.) Flore du Congo Belge et du Ruanda-Urundi, Spermatophytes, vol. I : 177–218. Bruxelles, I.N.E.A.C. [version PDF, 10.5281/zenodo.4266265] [46 pages][pdf][fr][Congo]
131 Hauman L. (1948) Podostemaceae. In : Boutique R. (éd.) Flore du Congo Belge et du Ruanda-Urundi, Spermatophytes, vol. I : 219–226. Bruxelles, I.N.E.A.C. [version PDF, 10.5281/zenodo.4266274] [12 pages][pdf][fr][Congo]
132 Hauman L. (1948) Proteaceae. In : Boutique R. (éd.) Flore du Congo Belge et du Ruanda-Urundi, Spermatophytes, vol. I : 227–248. Bruxelles, I.N.E.A.C. [version PDF, 10.5281/zenodo.4266282] [26 pages][pdf][fr][Congo]
133 Louis J. & Léonard J. (1948) Olacaceae. In : Boutique R. (éd.) Flore du Congo Belge et du Ruanda-Urundi, Spermatophytes, vol. I : 249–278. Bruxelles, I.N.E.A.C. [version PDF, 10.5281/zenodo.4266315] [34 pages][pdf][fr][Congo]
134 Louis J. & Léonard J. (1948) Opiliaceae. In : Boutique R. (éd.) Flore du Congo Belge et du Ruanda-Urundi, Spermatophytes, vol. I : 279–287. Bruxelles, I.N.E.A.C. [version PDF, 10.5281/zenodo.4279397] [13 pages][pdf][fr][Congo]
135 Louis J. & Léonard J. (1948) Octoknemaceae. In : Boutique R. (éd.) Flore du Congo Belge et du Ruanda-Urundi, Spermatophytes, vol. I : 288–293. Bruxelles, I.N.E.A.C. [version PDF, 10.5281/zenodo.4279404] [11 pages][pdf][fr][Congo]
136 Robyns W. & Lawalrée A. (1948) Santalaceae. In : Boutique R. (éd.) Flore du Congo Belge et du Ruanda-Urundi, Spermatophytes, vol. I : 294–303. Bruxelles, I.N.E.A.C. [version PDF, 10.5281/zenodo.4279427] [14 pages][pdf][fr][Congo]
137 Balle S. (1948) Loranthaceae. In : Boutique R. (éd.) Flore du Congo Belge et du Ruanda-

Urundi, Spermatophytes, vol. I : 304–380. Bruxelles, I.N.E.A.C. [version PDF, 10.5281/zenodo.4279439] [82 pages][pdf][fr][Congo]
138 Hauman L. (1948) Aristolochiaceae. In : Boutique R. (éd.) Flore du Congo Belge et du Ruanda-Urundi, Spermatophytes, vol. I : 381–389. Bruxelles, I.N.E.A.C. [version PDF, 10.5281/zenodo.4280823] [13 pages][pdf][fr][Congo]
139 Robyns W., Boutique R. (1948) Rafflesiaceae. In : Boutique R. (éd.) Flore du Congo Belge et du Ruanda-Urundi, Spermatophytes, vol. I : 390–391. Bruxelles, I.N.E.A.C. [version PDF, 10.5281/zenodo.4280829] [6 pages][pdf][fr][Congo]
140 Lebrun J. (1948) Hydnoraceae. In : Boutique R. (éd.) Flore du Congo Belge et du Ruanda-Urundi, Spermatophytes, vol. I : 392–393. Bruxelles, I.N.E.A.C. [version PDF, 10.5281/zenodo.4280834] [6 pages][pdf][fr][Congo]
141 Staner P. (1948) Balanophoraceae. In : Boutique R. (éd.) Flore du Congo Belge et du Ruanda-Urundi, Spermatophytes, vol. I : 394–395. Bruxelles, I.N.E.A.C. [version PDF, 10.5281/zenodo.4280837] [6 pages][pdf][fr][Congo]
142 Robyns W. (1948) Polygonaceae. In : Boutique R. (éd.) Flore du Congo Belge et du Ruanda-Urundi, Spermatophytes, vol. I : 396–427. Bruxelles, I.N.E.A.C. [version PDF, 10.5281/zenodo.4280843] [37 pages][pdf][fr][Congo]
143 Hauman L. (1951) Chenopodiaceae. In : Boutique R. (éd.) Flore du Congo Belge et du Ruanda-Urundi, Spermatophytes, vol. II : 1–11. Bruxelles, I.N.E.A.C. [version PDF, 10.5281/zenodo.4285787] [15 pages][pdf][fr][Congo]
144 Hauman L. (1951) Amaranthaceae. In : Boutique R. (éd.) Flore du Congo Belge et du Ruanda-Urundi, Spermatophytes, vol. II : 12–81. Bruxelles, I.N.E.A.C. [version PDF, 10.5281/zenodo.4300177] [74 pages][pdf][fr][Congo]
145 Balle S. (1951) Nyctaginaceae. In : Boutique R. (éd.) Flore du Congo Belge et du Ruanda-Urundi, Spermatophytes, vol. II : 82–91. Bruxelles, I.N.E.A.C. [version PDF, 10.5281/zenodo.4300234] [14 pages][pdf][fr][Congo]
146 Balle S. (1951) Phytolaccaceae. In : Boutique R. (éd.) Flore du Congo Belge et du Ruanda-Urundi, Spermatophytes, vol. II : 92–99. Bruxelles, I.N.E.A.C. [version PDF, 10.5281/zenodo.4300269] [12 pages][pdf][fr][Congo]
147 Hauman L.. (1951) Aizoaceae. In : Boutique R. (éd.) Flore du Congo Belge et du Ruanda-Urundi, Spermatophytes, vol. II : 100–117. Bruxelles, I.N.E.A.C. [version PDF, 10.5281/zenodo.4303721] [22 pages][pdf][fr][Congo]
148 Hauman L.. (1951) Portulacaceae. In : Boutique R. (éd.) Flore du Congo Belge et du Ruanda-Urundi, Spermatophytes, vol. II : 118–127. Bruxelles, I.N.E.A.C. [version PDF, 10.5281/zenodo.4303738] [14 pages][pdf][fr][Congo]
149 Hauman L.. (1951) Basellaceae. In : Boutique R. (éd.) Flore du Congo Belge et du Ruanda-Urundi, Spermatophytes, vol. II : 128–129. Bruxelles, I.N.E.A.C. [version PDF, 10.5281/zenodo.4303748] [6 pages][pdf][fr][Congo]
150 Balle S.. (1951) Caryophyllaceae. In : Boutique R. (éd.) Flore du Congo Belge et du Ruanda-Urundi, Spermatophytes, vol. II : 130–152. Bruxelles, I.N.E.A.C. [version PDF, 10.5281/zenodo.4303759] [27 pages][pdf][fr][Congo]
151 Hauman L.. (1951) Nymphaeaceae. In : Boutique R. (éd.) Flore du Congo Belge et du Ruanda-Urundi, Spermatophytes, vol. II : 153–164. Bruxelles, I.N.E.A.C. [version PDF, 10.5281/zenodo.4303778] [16 pages][pdf][fr][Congo]

152 Boutique R. (1951) Annonaceae. In : Boutique R. (éd.) Flore du Congo Belge et du Ruanda-Urundi, Spermatophytes, vol. II : 256–389. Bruxelles, I.N.E.A.C. [version PDF, 10.5281/zenodo.4303800] [138 pages][pdf][fr][Congo]
153 Staner P., Léonard J.. (1951) Ranunculaceae. In : Boutique R. (éd.) Flore du Congo Belge et du Ruanda-Urundi, Spermatophytes, vol. II : 167–201. Bruxelles, I.N.E.A.C. [version PDF, 10.5281/zenodo.4303825] [40 pages][pdf][fr][Congo]
154 Troupin G. (1951) Menispermaceae. In : Boutique R. (éd.) Flore du Congo Belge et du Ruanda-Urundi, Spermatophytes, vol. II : 202–255. Bruxelles, I.N.E.A.C. [version PDF, 10.5281/zenodo.4303940] [58 pages][pdf][fr][Congo]
155 Gilbert G., Troupin G. (1951) Myristicaceae. In : Boutique R. (éd.) Flore du Congo Belge et du Ruanda-Urundi, Spermatophytes, vol. II : 400–409. Bruxelles, I.N.E.A.C. [version PDF, 10.5281/zenodo.4303978] [14 pages][pdf][fr][Congo]
156 Léonard J. (1951) Monimiaceae. In : Boutique R. (éd.) Flore du Congo Belge et du Ruanda-Urundi, Spermatophytes, vol. II : 410–412. Bruxelles, I.N.E.A.C. [version PDF, 10.5281/zenodo.4303987] [7 pages][pdf][fr][Congo]
157 Robyns W., Wilczek R. (1951) Lauraceae. In : Boutique R. (éd.) Flore du Congo Belge et du Ruanda-Urundi, Spermatophytes, vol. II : 403–445. Bruxelles, I.N.E.A.C. [version PDF, 10.5281/zenodo.4304005] [51 pages][pdf][fr][Congo]
158 Boutique R. (1951) Hernandiaceae. In : Boutique R. (éd.) Flore du Congo Belge et du Ruanda-Urundi, Spermatophytes, vol. II : 446–449. Bruxelles, I.N.E.A.C. [version PDF, 10.5281/zenodo.4304013] [8 pages][pdf][fr][Congo]
159 Léonard J. (1951) Fumariaceae. In : Boutique R. (éd.) Flore du Congo Belge et du Ruanda-Urundi, Spermatophytes, vol. II : 450–453. Bruxelles, I.N.E.A.C. [version PDF, 10.5281/zenodo.4304028] [8 pages][pdf][fr][Congo]
160 Hauman L., Wilczek R. (1951) Capparidaceae. In : Boutique R. (éd.) Flore du Congo Belge et du Ruanda-Urundi, Spermatophytes, vol. II : 454–520. Bruxelles, I.N.E.A.C. [version PDF, 10.5281/zenodo.4304058] [73 pages][pdf][fr][Congo]
161 Robyns W., Boutique R. (1951) Cruciferae. In : Boutique R. (éd.) Flore du Congo Belge et du Ruanda-Urundi, Spermatophytes, vol. II : 522–543. Bruxelles, I.N.E.A.C. [version PDF, 10.5281/zenodo.4304073] [26 pages][pdf][fr][Congo]
162 Robyns W. (1951) Resedaceae. In : Boutique R. (éd.) Flore du Congo Belge et du Ruanda-Urundi, Spermatophytes, vol. II : 544–546. Bruxelles, I.N.E.A.C. [version PDF, 10.5281/zenodo.4304090] [7 pages][pdf][fr][Congo]
163 Robyns W. (1951) Moringaceae. In : Boutique R. (éd.) Flore du Congo Belge et du Ruanda-Urundi, Spermatophytes, vol. II : 547–548. Bruxelles, I.N.E.A.C. [version PDF, 10.5281/zenodo.4304111] [6 pages][pdf][fr][Congo]
164 Taton A. (1951) Droseraceae. In : Boutique R. (éd.) Flore du Congo Belge et du Ruanda-Urundi, Spermatophytes, vol. II : 549–556. Bruxelles, I.N.E.A.C. [version PDF, 10.5281/zenodo.4304126] [12 pages][pdf][fr][Congo]
165 Toussaint L. (1951) Crassulaceae. In : Boutique R. (éd.) Flore du Congo Belge et du Ruanda-Urundi, Spermatophytes, vol. II : 557–573. Bruxelles, I.N.E.A.C. [version PDF, 10.5281/zenodo.4304203] [21 pages][pdf][fr][Congo]
166 Léonard J. (1951) Pittosporaceae. In : Boutique R. (éd.) Flore du Congo Belge et du Ruanda-Urundi, Spermatophytes, vol. II : 574–580. Bruxelles, I.N.E.A.C. [version PDF,

10.5281/zenodo.4304213] [11 pages][pdf][fr][Congo]
167 Germain R. (1951) Hamamelidaceae. In : Boutique R. (éd.) Flore du Congo Belge et du Ruanda-Urundi, Spermatophytes, vol. II : 581–583. Bruxelles, I.N.E.A.C. [version PDF, 10.5281/zenodo.4304222] [7 pages][pdf][fr][Congo]
168 Hauman L. (1952) Rosaceae. In : Boutique R. (éd.) Flore du Congo Belge et du Ruanda-Urundi, Spermatophytes, vol. III : 1–69. Bruxelles, I.N.E.A.C. [version PDF, 10.5281/zenodo.4305899] [75 pages][pdf][fr][Congo]
169 Troupin G. (1952) Connaraceae. In : Boutique R. (éd.) Flore du Congo Belge et du Ruanda-Urundi, Spermatophytes, vol. III : 70–136. Bruxelles, I.N.E.A.C. [version PDF, 10.5281/zenodo.4305921] [71 pages][pdf][fr][Congo]
170 Gilbert G., Boutique R. (1952) Mimosaceae. In : Boutique R. (éd.) Flore du Congo Belge et du Ruanda-Urundi, Spermatophytes, vol. III : 137–233. Bruxelles, I.N.E.A.C. [version PDF, 10.5281/zenodo.4305933] [104 pages][pdf][fr][Congo]
171 Wilczek R. Léonard J., Hauman L., Hoyle A.C. Steyaert R. Gilbert G., Boutique R. (1952) Caesalpiniaceae I. - Dimorphandreae. Spermatophytes, vol. III : 237–233. Caesalpiniaceae II. - Eucaesalpinieae. Spermatophytes, vol. III : 247–264. Caesalpiniaceae III. - Bauhinieae. Spermatophytes, vol. III : 265–278. Caesalpiniaceae IV. -Cynometreae et Amherstieae. Spermatophytes, vol. III : 279–495. Caesalpiniaceae V. - Cassieae. Spermatophytes, vol. III : 495–545. Caesalpiniaceae VI. - Amphimanteae. Spermatophytes, vol. III : 546–549. Caesalpiniaceae VII. - Swartzieae. Spermatophytes, vol. III : 550–554. In : Boutique R. (éd.) Flore du Congo Belge et du Ruanda-Urundi, Bruxelles, I.N.E.A.C. [version PDF, 10.5281/zenodo.4308580] [366 pages][pdf][fr][Congo]
172 Toussaint L. Wilczek R. Gillett J.B. Boutique R. (1953) Papilionaceae (première partie) I. - Sophoreae. Spermatophytes, vol. IV : 4–45. Papilionaceae (première partie) II. - Genisteae. Spermatophytes, vol. IV : 46–288. Papilionaceae (première partie) III. - Trifolieae. Spermatophytes, vol. IV : 289–300. Papilionaceae (première partie) IV. - Loteae. Spermatophytes, vol. IV : 301–304. In : Boutique R. (éd.) Flore du Congo Belge et du Ruanda-Urundi, Bruxelles, I.N.E.A.C. [version PDF, 10.5281/zenodo.4308595] [326 pages][pdf][fr][Congo]
173 Hauman L., Cronquist A. Léonard J., Schubert B., Duvigneaud P., Dewit J. (1954) Papilionaceae (deuxième partie) V. -Galegeae. Spermatophytes, vol. V : 4–175. Papilionaceae (deuxième partie) VI. - Hedysareae. Spermatophytes, vol. V : 176–359. In : Boutique R. (éd.) Flore du Congo Belge et du Ruanda-Urundi, Bruxelles, I.N.E.A.C. [version PDF, 10.5281/zenodo.4309665] [387 pages][pdf][fr][Congo]
174 Hauman L., Cronquist A. Boutique R. Hauman L., Majot-Rocher R., Duvigneaud P., Robyns W., Wilczek R. (1954) Papilionaceae (troisième partie) VII. - Dalbergieae. Spermatophytes, vol. VI : 4–75. Papilionaceae (troisième partie) VIII. - Vicieae Spermatophytes, vol. VI : 76–86. Papilionaceae (troisième partie) IX. - Phaseoleae. Spermatophytes, vol. VI : 87–409. In : Boutique R. (éd.) Flore du Congo Belge et du Ruanda-Urundi, Bruxelles, I.N.E.A.C. [version PDF, 10.5281/zenodo.4309685] [436 pages][pdf][fr][Congo]
175 Robyns W. (1958) Pandaceae. In : Boutique R. (éd.) Flore du Congo Belge et du Ruanda-Urundi, Spermatophytes, vol. VII : 1–4. Bruxelles, I.N.E.A.C. [version PDF, 10.5281/zenodo.4327497] [8 pages][pdf][fr][Congo]

176 Wilczek R. (1958) Oxalidaceae. In : Boutique R. (éd.) Flore du Congo Belge et du Ruanda-Urundi, Spermatophytes, vol. VII : 5–19. Bruxelles, I.N.E.A.C. [version PDF, 10.5281/zenodo.4406570] [19 pages][pdf][fr][Congo]
177 Petit E. (1958) Geraniaceae. In : Boutique R. (éd.) Flore du Congo Belge et du Ruanda-Urundi, Spermatophytes, vol. VII : 20–28. Bruxelles, I.N.E.A.C. [version PDF, 10.5281/zenodo.4406614] [13 pages][pdf][fr][Congo]
178 Wilczek R., Boutique R. (1958) Linaceae. In : Boutique R. (éd.) Flore du Congo Belge et du Ruanda-Urundi, Spermatophytes, vol. VII : 29–53. Bruxelles, I.N.E.A.C. [version PDF, 10.5281/zenodo.4406665] [29 pages][pdf][fr][Congo]
179 Gilbert G. (1958) Erythroxylaceae. In : Boutique R. (éd.) Flore du Congo Belge et du Ruanda-Urundi, Spermatophytes, vol. VII : 54–57. Bruxelles, I.N.E.A.C. [version PDF, 10.5281/zenodo.4408813] [8 pages][pdf][fr][Congo]
180 Léonard J. (1958) Lepidobotryaceae. In : Boutique R. (éd.) Flore du Congo Belge et du Ruanda-Urundi, Spermatophytes, vol. VII : 58–61. Bruxelles, I.N.E.A.C. [version PDF, 10.5281/zenodo.4408815] [8 pages][pdf][fr][Congo]
181 Gilbert G. (1958) Zygophyllaceae. In : Boutique R. (éd.) Flore du Congo Belge et du Ruanda-Urundi, Spermatophytes, vol. VII : 62-64. Bruxelles, I.N.E.A.C. [version PDF, 10.5281/zenodo.4408820] [7 pages][pdf][fr][Congo]
182 Gilbert G. (1958) Balanitaceae. In : Boutique R. (éd.) Flore du Congo Belge et du Ruanda-Urundi, Spermatophytes, vol. VII : 65-68. Bruxelles, I.N.E.A.C. [version PDF, 10.5281/zenodo.4408838] [8 pages][pdf][fr][Congo]
183 Gilbert G. (1958) Rutaceae. In : Boutique R. (éd.) Flore du Congo Belge et du Ruanda-Urundi, Spermatophytes, vol. VII : 69-108. Bruxelles, I.N.E.A.C. [version PDF, 10.5281/zenodo.4408843] [44 pages][pdf][fr][Congo]
184 Gilbert G. (1958) Irvingiaceae. In : Boutique R. (éd.) Flore du Congo Belge et du Ruanda-Urundi, Spermatophytes, vol. VII : 109–118. Bruxelles, I.N.E.A.C. [version PDF, 10.5281/zenodo.4408880] [14 pages][pdf][fr][Congo]
185 Gilbert G. (1958) Simaroubaceae. In : Boutique R. (éd.) Flore du Congo Belge et du Ruanda-Urundi, Spermatophytes, vol. VII : 119–131. Bruxelles, I.N.E.A.C. [version PDF, 10.5281/zenodo.4408956] [17 pages][pdf][fr][Congo]
186 Troupin G. (1958) Burseraceae. In : Boutique R. (éd.) Flore du Congo Belge et du Ruanda-Urundi, Spermatophytes, vol. VII : 132–146. Bruxelles, I.N.E.A.C. [version PDF, 10.5281/zenodo.4408992] [21 pages][pdf][fr][Congo]
187 Staner P., Gilbert G. (1958) Meliaceae. In : Boutique R. (éd.) Flore du Congo Belge et du Ruanda-Urundi, Spermatophytes, vol. VII : 147–213. Bruxelles, I.N.E.A.C. [version PDF, 10.5281/zenodo.4409075] [72 pages][pdf][fr][Congo]
188 Wilczek R. (1958) Malpighiaceae. In : Boutique R. (éd.) Flore du Congo Belge et du Ruanda-Urundi, Spermatophytes, vol. VII : 214–234. Bruxelles, I.N.E.A.C. [version PDF, 10.5281/zenodo.4409155] [25 pages][pdf][fr][Congo]
189 Robyns W. (1958) Vochysiaceae. In : Boutique R. (éd.) Flore du Congo Belge et du Ruanda-Urundi, Spermatophytes, vol. VII : 235–237. Bruxelles, I.N.E.A.C. [version PDF, 10.5281/zenodo.4409159] [7 pages][pdf][fr][Congo]
190 Petit E.. (1958) Polygalaceae. In : Boutique R. (éd.) Flore du Congo Belge et du Ruanda-Urundi, Spermatophytes, vol. VII : 238–286. Bruxelles, I.N.E.A.C. [version PDF,

10.5281/zenodo.4409168] [53 pages][pdf][fr][Congo]
191 Hauman L. (1958) Dichapetalaceae. In : Boutique R. (éd.) Flore du Congo Belge et du Ruanda-Urundi, Spermatophytes, vol. VII : 287–348. Bruxelles, I.N.E.A.C. [version PDF, 10.5281/zenodo.4409177] [66 pages][pdf][fr][Congo]
192 Robyns A. (1958) Callitrichaceae. In : Boutique R. (éd.) Flore du Congo Belge et du Ruanda-Urundi, Spermatophytes, vol. VII : 349–351. Bruxelles, I.N.E.A.C. [version PDF, 10.5281/zenodo.4409222] [7 pages][pdf][fr][Congo]
193 Léonard J. (1962) Euphorbiaceae, 1re partie. In : Boutique R. (éd.) Flore du Congo Belge et du Ruanda-Urundi, Spermatophytes, vol. VIII : 1–214. Bruxelles, I.N.E.A.C. [version PDF, 10.5281/zenodo.4409266] [228 pages][pdf][fr][Congo]
194 Robyns W. (1960) Buxaceae. In : Boutique R. (éd.) Flore du Congo Belge et du Ruanda-Urundi, Spermatophytes, vol. IX : 1–4. Bruxelles, I.N.E.A.C. [version PDF, 10.5281/zenodo.4409276] [8 pages][pdf][fr][Congo]
195 Van der Veken P. (1960) Anacardiaceae. In : Boutique R. (éd.) Flore du Congo Belge et du Ruanda-Urundi, Spermatophytes, vol. IX : 5–108. Bruxelles, I.N.E.A.C. [version PDF, 10.5281/zenodo.4409279] [110 pages][pdf][fr][Congo]
196 Robyns A. (1960) Aquifoliaceae. In : Boutique R. (éd.) Flore du Congo Belge et du Ruanda-Urundi, Spermatophytes, vol. IX : 109–112. Bruxelles, I.N.E.A.C. [version PDF, 10.5281/zenodo.4411476] [8 pages][pdf][fr][Congo]
197 Wilczek R. (1960) Celastraceae. In : Boutique R. (éd.) Flore du Congo Belge et du Ruanda-Urundi, Spermatophytes, vol. IX : 113–132. Bruxelles, I.N.E.A.C. [version PDF, 10.5281/zenodo.4411521] [24 pages][pdf][fr][Congo]
198 Wilczek R. (1960) Hippocrateaceae. In : Boutique R. (éd.) Flore du Congo Belge et du Ruanda-Urundi, Spermatophytes, vol. IX : 133–232. Bruxelles, I.N.E.A.C. [version PDF, 10.5281/zenodo.4411590] [104 pages][pdf][fr][Congo]
199 Robyns W. (1960) Salvadoraceae. In : Boutique R. (éd.) Flore du Congo Belge et du Ruanda-Urundi, Spermatophytes, vol. IX : 233–236. Bruxelles, I.N.E.A.C. [version PDF, 10.5281/zenodo.4411693] [8 pages][pdf][fr][Congo]
200 Boutique R. (1960) Icacinaceae. In : Boutique R. (éd.) Flore du Congo Belge et du Ruanda-Urundi, Spermatophytes, vol. IX : 237–278. Bruxelles, I.N.E.A.C. [version PDF, 10.5281/zenodo.4411737] [46 pages][pdf][fr][Congo]
201 Hauman L. (1960) Sapindaceae. In : Boutique R. (éd.) Flore du Congo Belge et du Ruanda-Urundi, Spermatophytes, vol. IX : 279–384. Bruxelles, I.N.E.A.C. [version PDF, 10.5281/zenodo.4412880] [110 pages][pdf][fr][Congo]
202 Toussaint L. (1960) Melianthaceae. In : Boutique R. (éd.) Flore du Congo Belge et du Ruanda-Urundi, Spermatophytes, vol. IX : 385–395. Bruxelles, I.N.E.A.C. [version PDF, 10.5281/zenodo.4412957] [15 pages][pdf][fr][Congo]
203 Wilczek R., Schulze G.M. (1960) Balsaminaceae. In : Boutique R. (éd.) Flore du Congo Belge et du Ruanda-Urundi, Spermatophytes, vol. IX : 396–428. Bruxelles, I.N.E.A.C. [version PDF, 10.5281/zenodo.4413007] [37 pages][pdf][fr][Congo]
204 Évrard C. (1960) Rhamnaceae. In : Boutique R. (éd.) Flore du Congo Belge et du Ruanda-Urundi, Spermatophytes, vol. IX : 429–452. Bruxelles, I.N.E.A.C. [version PDF, 10.5281/zenodo.4413030] [28 pages][pdf][fr][Congo]
205 Dewit J., Willems L. (1960) Vitaceae. In : Boutique R. (éd.) Flore du Congo Belge et du

Ruanda-Urundi, Spermatophytes, vol. IX : 453–567. Bruxelles, I.N.E.A.C. [version PDF, 10.5281/zenodo.4413073] [119 pages][pdf][fr][Congo]
206 Dewit J. (1960) Leeaceae. In : Boutique R. (éd.) Flore du Congo Belge et du Ruanda-Urundi, Spermatophytes, vol. IX : 568–571. Bruxelles, I.N.E.A.C. [version PDF, 10.5281/zenodo.4413137] [8 pages][pdf][fr][Congo]
207 Wilczek R. (1963) Tiliaceae. In : Boutique R. (éd.) Flore du Congo Belge et du Ruanda-Urundi, Spermatophytes, vol. X : 1–91. Bruxelles, I.N.E.A.C. [version PDF, 10.5281/zenodo.4415797] [95 pages][pdf][fr][Congo]
208 Hauman L. (1963) Malvaceae. In : Boutique R. (éd.) Flore du Congo Belge et du Ruanda-Urundi, Spermatophytes, vol. X : 92–190. Bruxelles, I.N.E.A.C. [version PDF, 10.5281/zenodo.4415812] [103 pages][pdf][fr][Congo]
209 Robyns A. (1963) Bombacaceae. In : Boutique R. (éd.) Flore du Congo Belge et du Ruanda-Urundi, Spermatophytes, vol. X : 191–204. Bruxelles, I.N.E.A.C. [version PDF, 10.5281/zenodo.4416044] [18 pages][pdf][fr][Congo]
210 Germain R. (1963) Sterculiaceae. In : Boutique R. (éd.) Flore du Congo Belge et du Ruanda-Urundi, Spermatophytes, vol. X : 205–316. Bruxelles, I.N.E.A.C. [version PDF, 10.5281/zenodo.4416048] [116 pages][pdf][fr][Congo]
211 Germain R. (1963) Huaceae. In : Boutique R. (éd.) Flore du Congo Belge et du Ruanda-Urundi, Spermatophytes, vol. X : 317–319. Bruxelles, I.N.E.A.C. [version PDF, 10.5281/zenodo.4416052] [7 pages][pdf][fr][Congo]
212 Germain R. (1963) Scytopetalaceae. In : Boutique R. (éd.) Flore du Congo Belge et du Ruanda-Urundi, Spermatophytes, vol. X : 320–331. Bruxelles, I.N.E.A.C. [version PDF, 10.5281/zenodo.4416067] [16 pages][pdf][fr][Congo]
213 Bamps P., Geerinck D. (2005) Alangiaceae. In : Bamps P. (éd.) Flore d'Afrique centrale (Congo-Kinshasa – Rwanda – Burundi), Spermatophytes. Meise, Jardin botanique national de Belgique. [version PDF, 10.5281/zenodo.4420105] [11 pages][pdf][fr][Congo]
214 Symoens J.J., Billiet F. (1975) Alismataceae. In : Bamps P. (éd.) Flore d'Afrique centrale (Zaïre – Rwanda – Burundi), Spermatophytes. Meise, Jardin botanique national de Belgique. [version PDF, 10.5281/zenodo.4420967] [29 pages][pdf][fr][Congo]
215 Geerinck D. (1973) Amaryllidaceae. In : Bamps P. (éd.) Flore d'Afrique centrale (Zaïre – Rwanda – Burundi), Spermatophytes. Meise, Jardin botanique national de Belgique. [version PDF, 10.5281/zenodo.4420981] [31 pages][pdf][fr][Congo]
216 Léonard J. (1982) Ancistrocladaceae. In : Bamps P. (éd.) Flore d'Afrique centrale (Zaïre – Rwanda – Burundi), Spermatophytes. Meise, Jardin botanique national de Belgique. [version PDF, 10.5281/zenodo.4420996] [15 pages][pdf][fr][Congo]
217 Bruggen H.W.E. van (1974) Aponogetonaceae. In : Bamps P. (éd.) Flore d'Afrique centrale (Zaïre – Rwanda – Burundi), Spermatophytes. Meise, Jardin botanique national de Belgique. [version PDF, 10.5281/zenodo.4421301] [19 pages][pdf][fr][Congo]
218 Bamps P. (1974) Araliaceae. In : Bamps P. (éd.) Flore d'Afrique centrale (Zaïre – Rwanda – Burundi), Spermatophytes. Meise, Jardin botanique national de Belgique. [version PDF, 10.5281/zenodo.4421309] [37 pages][pdf][fr][Congo]
219 Wilczek R. (1969) Begoniaceae. In : Flore du Congo, du Rwanda et du Burundi, Spermatophytes. Meise, Jardin botanique national de Belgique. [version PDF, 10.5281/zenodo.4423272] [63 pages][pdf][fr][Congo]

220 Liben L. (1977) Bignoniaceae. In Bamps P. (éd.) Flore d'Afrique centrale (Zaïre - Rwanda – Burundi), Spermatophytes. Meise, Jardin botanique national de Belgique. [version PDF, 10.5281/zenodo.4423292] [47 pages][pdf][fr][Congo]
221 Lisowski L., Malaisse F., Symoens J.J. (1970) Bixaceae. In Boutique R. (éd.) Flore du Congo, du Rwanda et du Burundi, Spermatophytes. Meise, Jardin botanique national de Belgique. [version PDF, 10.5281/zenodo.4423336] [13 pages][pdf][fr][Congo]
222 Taton A. (1971) Boraginaceae. In Bamps P. (éd.) Flore du Congo, du Rwanda et du Burundi, Spermatophytes. Meise, Jardin botanique national de Belgique. [version PDF, 10.5281/zenodo.4423357] [89 pages][pdf][fr][Congo]
223 Geerinck D. (1970) Burmanniaceae. In : Flore du Congo, du Rwanda et du Burundi, Spermatophytes. Bruxelles, Jardin botanique national de Belgique. [version PDF, 10.5281/zenodo.4423375] [17 pages][pdf][fr][Congo]
223 Lisowski S., Malaisse F., Symoens J.J. (1970) Cabombaceae. In : Boutique R. (éd.), Flore du Congo, du Rwanda et du Burundi, Spermatophytes. Bruxelles, Jardin botanique national de Belgique. [version PDF, 10.5281/zenodo.4425585] [13 pages][pdf][fr][Congo]
224 Robyns W. (1967) Cactaceae. In : Flore du Congo, du Rwanda et du Burundi, Spermatophytes. Bruxelles, Jardin botanique national de Belgique. [version PDF, 10.5281/zenodo.4425585] [13 pages][pdf][fr][Congo]
225 Thulin M. (1977) Campanulaceae. In : Bamps P. (éd.), Flore d'Afrique centrale (Zaïre - Rwanda - Burundi), Spermatophytes. Meise, Jardin botanique national de Belgique. [version PDF, 10.5281/zenodo.4425654] [59 pages][pdf][fr][Congo]
226 Boutique R. (1967) Canellaceae. In : Flore du Congo, du Rwanda et du Burundi, Spermatophytes. Bruxelles, Jardin botanique national de Belgique. [version PDF, 10.5281/zenodo.4429113] [13 pages][pdf][fr][Congo]
227 Lawalrée A. (1982) Caprifoliaceae. In : Bamps P. (éd.), Flore d'Afrique centrale (Zaïre - Rwanda – Burundi), Spermatophytes. Meise, Jardin botanique national de Belgique. [version PDF, 10.5281/zenodo.4429121] [13 pages][pdf][fr][Congo]
228 Robyns W. (1967) Cochlospermaceae. In : Flore du Congo, du Rwanda et du Burundi, Spermatophytes. Bruxelles, Jardin botanique national de Belgique. [version PDF, 10.5281/zenodo.4429121] [13 pages][pdf][fr][Congo]
229 Liben L. (1968) Combretaceae. In : Flore du Congo, du Rwanda et du Burundi, Spermatophytes. Bruxelles, Jardin botanique national de Belgique. [version PDF, 10.5281/zenodo.4514585] [117 pages][pdf][fr][Congo]
230 Lawalrée A., Dethier D, Gilissen E. (1986) Compositae – Première partie. In : Bamps P. (éd.) Flore d'Afrique centrale (Zaïre – Rwanda – Burundi), Spermatophytes. Bruxelles, Jardin botanique national de Belgique. [version PDF, 10.5281/zenodo.4514605] [78 pages][pdf][fr][Congo]
231 Lisowski S. (1989) Compositae – Deuxième partie. In : Bamps P. (éd.) Flore d'Afrique centrale (Zaïre – Rwanda – Burundi), Spermatophytes. Bruxelles, Jardin botanique national de Belgique. [version PDF, 10.5281/zenodo.4514617] [244 pages][pdf][fr][Congo]
232 Bamps P. (1971) Cornaceae. In : Bamps P. (éd.) Flore du Congo, du Rwanda et du Burundi, Spermatophytes. Bruxelles, Jardin botanique national de Belgique. [version PDF, 10.5281/zenodo.4514625] [13 pages][pdf][fr][Congo]
233 Keraudren-Aymonin M. (1975) Cucurbitaceae. In : Bamps P. (éd.) Flore d'Afrique centrale

(Zaïre – Rwanda – Burundi), Spermatophytes. Bruxelles, Jardin botanique national de Belgique. [version PDF, 10.5281/zenodo.4515370] [161 pages][pdf][fr][Congo]

234 Lejoly J., Lisowski S. (1993) Cuscutaceae. In : Bamps P. (éd.) Flore d'Afrique centrale (Zaïre – Rwanda – Burundi), Spermatophytes. Bruxelles, Jardin botanique national de Belgique. [version PDF, 10.5281/zenodo.4515394] [17 pages][pdf][fr][Congo]

235 Boutique R. (1967) Dilleniaceae. In : Flore du Congo, du Rwanda et du Burundi, Spermatophytes. Bruxelles, Jardin botanique national de Belgique. [version PDF, 10.5281/zenodo.4516238] [21 pages][pdf][fr][Congo]

236 Lawalrée A. (1979) Dipsacaceae. In : Bamps P. (éd.) Flore d'Afrique centrale (Zaïre – Rwanda – Burundi), Spermatophytes. Bruxelles, Jardin botanique national de Belgique. [version PDF, 10.5281/zenodo.4516243] [16 pages][pdf][fr][Congo]

237 White F. (1987) Ebenaceae. In : Bamps P. (éd.) Flore d'Afrique centrale (Zaïre – Rwanda – Burundi), Spermatophytes. Bruxelles, Jardin botanique national de Belgique. [version PDF, 10.5281/zenodo.4519931] [109 pages][pdf][fr][Congo]

238 Boutique R. (1967) Elatinaceae. In : Flore du Congo, du Rwanda et du Burundi, Spermatophytes. Bruxelles, Jardin botanique national de Belgique. [version PDF, 10.5281/zenodo.4519976] [13 pages][pdf][fr][Congo]

239 Léonard J. (1996) Euphorbiaceae (troisième partie). In : Bamps P. (éd.) Flore d'Afrique centrale (Zaïre – Rwanda – Burundi), Spermatophytes. Meise, Jardin botanique national de Belgique. [version PDF, 10.5281/zenodo.4520003] [79 pages][pdf][fr][Congo]

240 Léonard J. (1995) Euphorbiaceae (deuxième partie). In : Bamps P. (éd.) Flore d'Afrique centrale (Zaïre – Rwanda – Burundi), Spermatophytes. Meise, Jardin botanique national de Belgique. [version PDF, 10.5281/zenodo.4520005] [124 pages][pdf][fr][Congo]

241 Bamps P. (1968) Flacourtiaceae (Première partie). In : Flore du Congo, du Rwanda et du Burundi, Spermatophytes. Bruxelles, Jardin botanique national de Belgique. [version PDF, 10.5281/zenodo.4541355] [71 pages][pdf][fr][Congo]

242 Sleumer H. (1976) Flacourtiaceae (seconde partie). In : Bamps P. (éd.) Flore d'Afrique centrale (Zaïre – Rwanda – Burundi), Spermatophytes. Meise, Jardin botanique national de Belgique. [version PDF, 10.5281/zenodo.4541375] [53 pages][pdf][fr][Congo]

243 Boutique R. (1972) Gentianaceae. In : Bamps P. (éd.) Flore d'Afrique centrale (Zaïre – Rwanda – Burundi), Spermatophytes. Bruxelles, Jardin botanique national de Belgique. [version PDF, 10.5281/zenodo.4541404] [65 pages][pdf][fr][Congo]

244 Bamps P. (1970) Guttiferae. In : Boutique R. (éd.) Flore du Congo, du Rwanda et du Burundi, Spermatophytes. Bruxelles, Jardin botanique national de Belgique. [version PDF, 10.5281/zenodo.4541421] [85 pages][pdf][fr][Congo]

245 Boutique R. (1968) Haloragaceae. In : Bamps P. (éd.) Flore du Congo, du Rwanda et du Burundi, Spermatophytes. Bruxelles, Jardin botanique national de Belgique. [version PDF, 10.5281/zenodo.4545688] [17 pages][pdf][fr][Congo]

246 Robyns A. (1976) Huaceae (nouvelle édition). In : Bamps P. (éd.) Flore d'Afrique centrale (Zaïre – Rwanda – Burundi), Spermatophytes. Meise, Jardin botanique national de Belgique. [version PDF, 10.5281/zenodo.4545698] [17 pages][pdf][fr][Congo]

247 Liben L. (1970) Humiriaceae. In : Flore du Congo, du Rwanda et du Burundi, Spermatophytes. Bruxelles, Jardin botanique national de Belgique. [version PDF, 10.5281/zenodo.4545720] [13 pages][pdf][fr][Congo]

248 Léonard J., Mosango M. (1985) Hymenocardiaceae. In : Bamps P. (éd.) Flore d'Afrique centrale (Zaïre – Rwanda – Burundi), Spermatophytes. Meise, Jardin botanique national de Belgique. [version PDF, 10.5281/zenodo.4545732] [21 pages][pdf][fr][Congo]
249 Geerinck D. (1971) Hypoxidaceae. In : Bamps P. (éd.) Flore du Congo, du Rwanda et du Burundi, Spermatophytes. Bruxelles, Jardin botanique national de Belgique. [version PDF, 10.5281/zenodo.4545746] [17 pages][pdf][fr][Congo]
250 Geerinck D. (2005) Iridaceae. In : Bamps P. (éd.) Flore d'Afrique centrale (Congo-Kinshasa – Rwanda – Burundi), Spermatophytes. Meise, Jardin botanique national de Belgique. [version PDF, 10.5281/zenodo.4564052] [117 pages][pdf][fr][Congo]
251 Lisowski S., Malaisse F, Symoens J.J. (1973) Juncaceae. In : Bamps P. (éd.) Flore d'Afrique centrale (Zaïre – Rwanda – Burundi), Spermatophytes. Meise, Jardin botanique national de Belgique. [version PDF, 10.5281/zenodo.4564077] [21 pages][pdf][fr][Congo]
252 Lisowski S., Malaisse F, Symoens J.J. (1982) Juncaginaceae. In : Bamps P. (éd.) Flore d'Afrique centrale (Zaïre – Rwanda – Burundi), Spermatophytes. Meise, Jardin botanique national de Belgique. [version PDF, 10.5281/zenodo.4564129] [13 pages][pdf][fr][Congo]
253 Liben L. (1971) Lecythidaceae. In : Bamps P. (éd.) Flore du Congo, du Rwanda et du Burundi, Spermatophytes. Bruxelles, Jardin botanique national de Belgique. [version PDF, 10.5281/zenodo.4564144] [17 pages][pdf][fr][Congo]
254 Landolt E., Lawalree A. (1988) Lemnaceae. In : Bamps P. (éd.) Flore d'Afrique centrale (Zaïre – Rwanda – Burundi), Spermatophytes. Meise, Jardin botanique national de Belgique. [version PDF, 10.5281/zenodo.4564159] [17 pages][pdf][fr][Congo]
255 Taylor P. (1972) Lentibulariaceae. In : Bamps P. (éd.) Flore d'Afrique centrale (Zaïre – Rwanda – Burundi), Spermatophytes. Bruxelles, Jardin botanique national de Belgique. [version PDF, 10.5281/zenodo.4564188] [71 pages][pdf][fr][Congo]
256 Thulin M. (1985) Lobeliaceae. In : Bamps P. (éd.) Flore d'Afrique centrale (Zaïre – Rwanda – Burundi), Spermatophytes. Meise, Jardin botanique national de Belgique. [version PDF, 10.5281/zenodo.4569920] [73 pages][pdf][fr][Congo]
257 Leeuwenberg A.J.M., Bamps P. (1979) Loganiaceae. In : Bamps P. (éd.) Flore d'Afrique centrale (Zaïre – Rwanda – Burundi), Spermatophytes. Meise, Jardin botanique national de Belgique. [version PDF, 10.5281/zenodo.4569937] [157 pages][pdf][fr][Congo]
258 Boutique R. (1967) Lythraceae. In : Flore du Congo, du Rwanda et du Burundi, Spermatophytes. Bruxelles, Jardin botanique national de Belgique. [version PDF, 10.5281/zenodo.4569946] [37 pages][pdf][fr][Congo]
259 Boutique R. (1971) Mayacaceae. In : Bamps P. (éd.) Flore du Congo, du Rwanda et du Burundi, Spermatophytes. Bruxelles, Jardin botanique national de Belgique. [version PDF, 10.5281/zenodo.4570190] [13 pages][pdf][fr][Congo]
260 Raynal A. (1975) Menyanthaceae. In : Bamps P. (éd.) Flore d'Afrique centrale (Zaïre – Rwanda – Burundi), Spermatophytes. Meise, Jardin botanique national de Belgique. [version PDF, 10.5281/zenodo.4570197] [21 pages][pdf][fr][Congo]
261 Lisowski S., Malaisse F., Symoens J.J. (1970) Myrothamnaceae. In : Boutique R. (éd.) Flore du Congo, du Rwanda et du Burundi, Spermatophytes. Bruxelles, Jardin botanique national de Belgique. [version PDF, 10.5281/zenodo.4570242] [13 pages][pdf][fr][Congo]
262 Taton A. (1980) Myrsinaceae. In : Bamps P. (éd.) Flore d'Afrique centrale (Zaïre – Rwanda – Burundi), Spermatophytes. Meise, Jardin botanique national de Belgique. [version PDF,

10.5281/zenodo.4572723] [70 pages][pdf][fr][Congo]
263 Boutique R. (1968) Myrtaceae. In : Flore du Congo, du Rwanda et du Burundi, Spermatophytes. Bruxelles, Jardin botanique national de Belgique. [version PDF, 10.5281/zenodo.4572735] [41 pages][pdf][fr][Congo]
264 Triest L., Symoens J.J. (1983) Najadaceae. In : Bamps P. (éd.) Flore d'Afrique centrale (Zaïre – Rwanda – Burundi), Spermatophytes. Meise, Jardin botanique national de Belgique. [version PDF, 10.5281/zenodo.4572745] [17 pages][pdf][fr][Congo]
265 Bamps P., Farron C. (1967) Ochnaceae. In : Flore du Congo, du Rwanda et du Burundi, Spermatophytes. Bruxelles, Jardin botanique national de Belgique. [version PDF, 10.5281/zenodo.4660110] [73 pages][pdf][fr][Congo]
266 Liben L. (1973) Oleaceae. In : Bamps P. (éd.) Flore d'Afrique centrale (Zaïre – Rwanda – Burundi), Spermatophytes. Meise, Jardin botanique national de Belgique. [version PDF, 10.5281/zenodo.4660117] [45 pages][pdf][fr][Congo]
267 Liben L. (1973) Oliniaceae. In : Bamps P. (éd.) Flore d'Afrique centrale (Zaïre – Rwanda – Burundi), Spermatophytes. Meise, Jardin botanique national de Belgique. [version PDF, 10.5281/zenodo.4660123] [13 pages][pdf][fr][Congo]
268 Taton A. (1967) Onagraceae. In : Flore du Congo, du Rwanda et du Burundi, Spermatophytes. Bruxelles, Jardin botanique national de Belgique. [version PDF, 10.5281/zenodo.4660230] [33 pages][pdf][fr][Congo]
269 Geerinck D. (1984) Orchidaceae (première partie). In : Bamps P. (éd.) Flore d'Afrique centrale (Zaïre – Rwanda – Burundi), Spermatophytes. Meise, Jardin botanique national de Belgique. [version PDF, 10.5281/zenodo.4660235] [308 pages][pdf][fr][Congo]
270 Geerinck D. (1992) Orchidaceae (seconde partie). In : Bamps P. (éd.) Flore d'Afrique centrale (Zaïre – Rwanda – Burundi), Spermatophytes. Meise, Jardin botanique national de Belgique. [version PDF, 10.5281/zenodo.4660246] [496 pages][pdf][fr][Congo]
271 Robyns A. (1995) Passifloraceae. In : Bamps P. (éd.) Flore d'Afrique centrale (Zaïre – Rwanda – Burundi), Spermatophytes. Meise, Jardin botanique national de Belgique. [version PDF, 10.5281/zenodo.4665394] [80 pages][pdf][fr][Congo]
272 Lisowski S., Malaisse F., Symoens J.J. (1972) Plantaginaceae. In : Bamps P. (éd.) Flore d'Afrique centrale (Zaïre – Rwanda – Burundi), Spermatophytes. Meise, Jardin botanique national de Belgique. [version PDF, 10.5281/zenodo.4665514] [12 pages][pdf][fr][Congo]
273 Lawalrée A., Bruynseels G. (1981) Pontederiaceae. In : Bamps P. (éd.) Flore d'Afrique centrale (Zaïre – Rwanda – Burundi), Spermatophytes. Meise, Jardin botanique national de Belgique. [version PDF, 10.5281/zenodo.4665585] [18 pages][pdf][fr][Congo]
274 Lisowski S., Malaisse F., Symoens J.J., Van de Velde J. (1978) Potamogetonaceae. In : Bamps P. (éd.) Flore d'Afrique centrale (Zaïre – Rwanda – Burundi), Spermatophytes. Meise, Jardin botanique national de Belgique. [version PDF, 10.5281/zenodo.4665747] [17 pages][pdf][fr][Congo]
275 Boutique R. (1971) Primulaceae. In : Bamps P. (éd.) Flore du Congo, du Rwanda et du Burundi, Spermatophytes. Bruxelles, Jardin botanique national de Belgique. [version PDF, 10.5281/zenodo.4669968] [25 pages][pdf][fr][Congo]
276 Liben L. (1987) Rhizophoraceae. In : Bamps P. (éd.) Flore d'Afrique centrale (Zaïre – Rwanda – Burundi), Spermatophytes. Meise, Jardin botanique national de Belgique. [version PDF, 10.5281/zenodo.4669992] [41 pages][pdf][fr][Congo]

277 Symoens J.J. (1977) Ruppiaceae. In : Bamps P. (éd.) Flore d'Afrique centrale (Zaïre – Rwanda – Burundi), Spermatophytes. Meise, Jardin botanique national de Belgique. [version PDF, 10.5281/zenodo.4670010] [13 pages][pdf][fr][Congo]
278 Léonard J., Geerinck D. (1967) Salicaceae. In : Flore du Congo, du Rwanda et du Burundi, Spermatophytes. Bruxelles, Jardin botanique national de Belgique. [version PDF, 10.5281/zenodo.4670038] [13 pages][pdf][fr][Congo]
279 Liben L. (1969) Saxifragaceae. In : Flore du Congo, du Rwanda et du Burundi, Spermatophytes. Bruxelles, Jardin botanique national de Belgique. [version PDF, 10.5281/zenodo.4670117] [13 pages][pdf][fr][Congo]
280 Fischer E. (1999) Scrophulariaceae (première partie). In : Bamps P. (éd.) Flore d'Afrique centrale (Zaïre – Rwanda – Burundi), Spermatophytes. Meise, Jardin botanique national de Belgique. [version PDF, 10.5281/zenodo.4670158] [225 pages][pdf][fr][Congo]
281 Thulin M. (1973) Sphenocleaceae. In : Bamps P. (éd.) Flore d'Afrique centrale (Zaïre – Rwanda – Burundi), Spermatophytes. Meise, Jardin botanique national de Belgique. [version PDF, 10.5281/zenodo.4672140] [14 pages][pdf][fr][Congo]
282 Lisowski S., Malaisse F., Symoens J.J. (1976) Taccaceae. In : Bamps P. (éd.) Flore d'Afrique centrale (Zaïre – Rwanda – Burundi), Spermatophytes. Meise, Jardin botanique national de Belgique. [version PDF, 10.5281/zenodo.4672148] [17 pages][pdf][fr][Congo]
283 Boutique R. (1967) Theaceae. In : Flore du Congo, du Rwanda et du Burundi, Spermatophytes. Bruxelles, Jardin botanique national de Belgique. [version PDF, 10.5281/zenodo.4672158] [17 pages][pdf][fr][Congo]
284 Robyns A. (1975) Thymelaeaceae. In : Bamps P. (éd.) Flore d'Afrique centrale (Zaïre – Rwanda – Burundi), Spermatophytes. Meise, Jardin botanique national de Belgique. [version PDF, 10.5281/zenodo.4672204] [77 pages][pdf][fr][Congo]
285 Symoens J.J. Symoens-Piqueray B. (2004) Trapaceae. In : Bamps P. (éd.) Flore d'Afrique centrale (Zaïre – Rwanda – Burundi), Spermatophytes. Meise, Jardin botanique national de Belgique. [version PDF, 10.5281/zenodo.4672214] [13 pages][pdf][fr][Congo]
286 Robyns W. (1967) Turneraceae. In : Flore du Congo, du Rwanda et du Burundi, Spermatophytes. Bruxelles, Jardin botanique national de Belgique. [version PDF, 10.5281/zenodo.4672222] [21 pages][pdf][fr][Congo]
287 Lawarlée A. (1972) Valerianaceae. In : Bamps P. (éd.) Flore d'Afrique centrale (Zaïre – Rwanda – Burundi), Spermatophytes. Bruzelles, Jardin botanique national de Belgique. [version PDF, 10.5281/zenodo.4675039] [13 pages][pdf][fr][Congo]
288 Taton A. (1969) Violaceae. In : Flore du Congo, du Rwanda et du Burundi, Spermatophytes. Bruxelles, Jardin botanique national de Belgique. [version PDF, 10.5281/zenodo.4675058] [85 pages][pdf][fr][Congo]
289 Lisowski S., Brylska B., Wiland-Szymańska J. (2001) Xyridaceae. In : Bamps P. (éd.) Flore d'Afrique centrale (Zaïre – Rwanda – Burundi), Spermatophytes. Meise, Jardin botanique national de Belgique. [version PDF, 10.5281/zenodo.4675064] [124 pages][pdf][fr][Congo]
290 Lawalrée A. (1969) Actiniopteridaceae. In : Flore du Congo, du Rwanda et du Burundi, Pteridophytes. Bruxelles, Jardin botanique national de Belgique. [version PDF, 10.5281/zenodo.4683154] [17 pages][pdf][fr][Congo]
291 Lawalrée A. (1976). Azollaceae. In : Bamps P. (éd.) Flore d'Afrique centrale (Zaïre – Rwanda – Burundi), Pteridophyta. Meise, Jardin botanique national de Belgique. [version PDF,

10.5281/zenodo.4683171] [13 pages][pdf][fr][Congo]
292 Lawalrée A. (1971) Blechnaceae. In : Boutique R. (éd.) Flore du Congo, du Rwanda et du Burundi, Pteridophytes. Bruxelles, Jardin botanique national de Belgique. [version PDF, 10.5281/zenodo.4683185] [23 pages][pdf][fr][Congo]
293 Lawalrée A. (1993). Davalliaceae. In : Bamps P. (éd.) Flore d'Afrique centrale (Zaïre – Rwanda – Burundi), Pteridophyta. Meise, Jardin botanique national de Belgique. [version PDF, 10.5281/zenodo.4683194] [13 pages][pdf][fr][Congo]
294 Lawalrée A. (1969) Equisetaceae. In : Flore du Congo, du Rwanda et du Burundi, Pteridophytes. Bruxelles, Jardin botanique national de Belgique. [version PDF, 10.5281/zenodo.4683254] [17 pages][pdf][fr][Congo]
295 Kramer K.U. (1971) Lindsaeaceae. In : Bamps P. (éd.) Flore du Congo, du Rwanda et du Burundi, Pteridophytes. Bruxelles, Jardin botanique national de Belgique. [version PDF, 10.5281/zenodo.4683363] [19 pages][pdf][fr][Congo]
296 Lawalrée A. (1989). Lycopodiaceae. In : Bamps P. (éd.) Flore d'Afrique centrale (Zaïre – Rwanda – Burundi), Pteridophyta. Meise, Jardin botanique national de Belgique. [version PDF, 10.5281/zenodo.4687069] [28 pages][pdf][fr][Congo]
297 Launert E. (1975). Marsileaceae. In : Bamps P. (éd.) Flore d'Afrique centrale (Zaïre – Rwanda – Burundi), Pteridophyta. Meise, Jardin botanique national de Belgique. [version PDF, 10.5281/zenodo.4687084] [17 pages][pdf][fr][Congo]
298 Lawalrée A. (2000). Nephrolepidaceae. In : Bamps P. (éd.) Flore d'Afrique centrale (Zaïre – Rwanda – Burundi), Pteridophyta. Meise, Jardin botanique national de Belgique. [version PDF, 10.5281/zenodo.4687107] [21 pages][pdf][fr][Congo]
299 Lewalle J. (1973). Osmundaceae. In : Bamps P. (éd.) Flore d'Afrique centrale (Zaïre – Rwanda – Burundi), Pteridophyta. Meise, Jardin botanique national de Belgique. [version PDF, 10.5281/zenodo.4687118] [15 pages][pdf][fr][Congo]
300 Lawalrée A. (1969) Parkeriaceae. In : Flore du Congo, du Rwanda et du Burundi, Pteridophytes. Bruxelles, Jardin botanique national de Belgique. [version PDF, 10.5281/zenodo.4694405] [15 pages][pdf][fr][Congo]
301 Lawalrée A. (1969) Psilotaceae. In : Flore du Congo, du Rwanda et du Burundi, Pteridophytes. Bruxelles, Jardin botanique national de Belgique. [version PDF, 10.5281/zenodo.4694428] [13 pages][pdf][fr][Congo]
302 Lawalrée A. (1970) Schizaeaceae. In : Boutique R. (éd.) Flore du Congo, du Rwanda et du Burundi, Pteridophytes. Bruxelles, Jardin botanique national de Belgique. [version PDF, 10.5281/zenodo.4694436] [23 pages][pdf][fr][Congo]
303 Meerts P. (2015). Anthericaceae. In : Sosef M.S.M. (éd.) Flore d'Afrique centrale (République démocratique du Congo - Rwanda - Burundi), nouvelle série, Spermatophyta. Meise, Jardin botanique de Meise. [version PDF, 10.5281/zenodo.4738995] [123 pages][pdf][fr][Congo]
304 Geerinck D.J.L. (2016). Asparagaceae. In : Sosef M.S.M. (éd.) Flore d'Afrique centrale (République démocratique du Congo - Rwanda - Burundi), nouvelle série, Spermatophyta. Meise, Jardin botanique de Meise. [version PDF, 10.5281/zenodo.4739078] [41 pages][pdf][fr][Congo]
305 Geerinck D.J.L. (2015). Asphodelaceae. In : Sosef M.S.M. (éd.) Flore d'Afrique centrale (République démocratique du Congo - Rwanda - Burundi), nouvelle série, Spermatophyta.

Meise, Jardin botanique de Meise. [version PDF, 10.5281/zenodo.4739105] [47 pages][pdf][fr][Congo]
306 Leal M.E. (2015). Bromeliaceae. In : Sosef M.S.M. (éd.) Flore d'Afrique centrale (République démocratique du Congo - Rwanda - Burundi), nouvelle série, Spermatophyta. Meise, Jardin botanique de Meise. [version PDF, 10.5281/zenodo.5647157] [15 pages][pdf][fr][Congo]
307 Maas P.J.M. & Maas-van de Kamer H. (2017). Cannaceae. In : Sosef M.S.M. (éd.) Flore d'Afrique centrale (République démocratique du Congo - Rwanda - Burundi), nouvelle série, Spermatophyta. Meise, Jardin botanique de Meise. [version PDF, 10.5281/zenodo.5647185] [15 pages][pdf][fr][Congo]
308 Ntore S. (2014). Caricaceae. In : Sosef M.S.M. (éd.) Flore d'Afrique centrale (République démocratique du Congo - Rwanda - Burundi), nouvelle série, Spermatophyta. Meise, Jardin botanique de Meise. [version PDF, 10.5281/zenodo.5647210] [17 pages][pdf][fr][Congo]
309 Geerinck (2014). Colchicaceae. In : Sosef M.S.M. (éd.) Flore d'Afrique centrale (République démocratique du Congo - Rwanda - Burundi), nouvelle série, Spermatophyta. Meise, Jardin botanique de Meise. [version PDF, 10.5281/zenodo.5647232] [25 pages][pdf][fr][Congo]

310 Meerts P. (2017). Dipterocarpaceae. In : Sosef M.S.M. (éd.) Flore d'Afrique centrale (République démocratique du Congo - Rwanda - Burundi), nouvelle série, Spermatophyta. Meise, Jardin botanique de Meise. [version PDF, 10.5281/zenodo.5647798] [25 pages][pdf][fr][Congo]
311 Geerinck D. (2014). Ericaceae. In : Sosef M.S.M. (éd.) Flore d'Afrique centrale (République démocratique du Congo - Rwanda - Burundi), nouvelle série, Spermatophyta. Meise, Jardin botanique de Meise. [version PDF, 10.5281/zenodo.5647840] [37 pages][pdf][fr][Congo]
312 Geerinck D. (2014). Flagellariaceae. In : Sosef M.S.M. (éd.) Flore d'Afrique centrale (République démocratique du Congo - Rwanda - Burundi), nouvelle série, Spermatophyta. Meise, Jardin botanique de Meise. [version PDF, 10.5281/zenodo.5647855] [17 pages][pdf][fr][Congo]
313 Sosef M.S.M. (2017). Goodeniaceae. In : Sosef M.S.M. (éd.) Flore d'Afrique centrale (République démocratique du Congo - Rwanda - Burundi), nouvelle série, Spermatophyta. Meise, Jardin botanique de Meise. [version PDF, 10.5281/zenodo.5647877] [15 pages][pdf][fr][Congo]
314 Sosef M.S.M. (2017). Gramineae, Introduction, Tribu I. Phareae à X. Bambuseae. In : Sosef M.S.M. (éd.) Flore d'Afrique centrale (République démocratique du Congo - Rwanda - Burundi), nouvelle série, Spermatophyta. Meise, Jardin botanique de Meise. [version PDF, 10.5281/zenodo.5647893] [89 pages][pdf][fr][Congo]
315 Maas P.J.M. & Maas-van de Kamer H. (2017). Heliconiaceae. In : Sosef M.S.M. (éd.) Flore d'Afrique centrale (République démocratique du Congo - Rwanda - Burundi), nouvelle série, Spermatophyta. Meise, Jardin botanique de Meise. [version PDF, 10.5281/zenodo.5647962] [15 pages][pdf][fr][Congo]
316 Symoens J.-J. & Sosef M.S.M. (2015). Hydrocharitaceae. In : Sosef M.S.M. (éd.) Flore

d'Afrique centrale (République démocratique du Congo - Rwanda - Burundi), nouvelle série, Spermatophyta. Meise, Jardin botanique de Meise. [version PDF, 10.5281/zenodo.5647972] [51 pages][pdf][fr][Congo]

317 Fischer E. (2015). Hydroleaceae. In : Sosef M.S.M. (éd.) Flore d'Afrique centrale (République démocratique du Congo - Rwanda - Burundi), nouvelle série, Spermatophyta. Meise, Jardin botanique de Meise. [version PDF, 10.5281/zenodo.5647991] [15 pages][pdf][fr][Congo]

318 Byng J.W. (2015). Ixonanthaceae. In : Sosef M.S.M. (éd.) Flore d'Afrique centrale (République démocratique du Congo - Rwanda - Burundi), nouvelle série, Spermatophyta. Meise, Jardin botanique de Meise. [version PDF, 10.5281/zenodo.5648012] [19 pages][pdf][fr][Congo]

319 Dhetchuvi J.B.M.M. & Ley A.C. (2017). Marantaceae. In : Sosef M.S.M. (éd.) Flore d'Afrique centrale (République démocratique du Congo - Rwanda - Burundi), nouvelle série, Spermatophyta. Meise, Jardin botanique de Meise. [version PDF, 10.5281/zenodo.5648035] [83 pages][pdf][fr][Congo]

320 Janssens S.B. (2016). Musaceae. In : Sosef M.S.M. (éd.) Flore d'Afrique centrale (République démocratique du Congo - Rwanda - Burundi), nouvelle série, Spermatophyta. Meise, Jardin botanique de Meise. [version PDF, 10.5281/zenodo.5652832] [21 pages][pdf][fr][Congo]

321 Byng J.W. (2015). Picrodendraceae. In : Sosef M.S.M. (éd.) Flore d'Afrique centrale (République démocratique du Congo - Rwanda - Burundi), nouvelle série, Spermatophyta. Meise, Jardin botanique de Meise. [version PDF, 10.5281/zenodo.5652862] [15 pages][pdf][fr][Congo]

322 Beentje H.J. (2016). Pandanaceae. In : Sosef M.S.M. (éd.) Flore d'Afrique centrale (République démocratique du Congo - Rwanda - Burundi), nouvelle série, Spermatophyta. Meise, Jardin botanique de Meise. [version PDF, 10.5281/zenodo.5652862] [21 pages][pdf][fr][Congo]

323 Ntore S. (2015). Plumbaginaceae. In : Sosef M.S.M. (éd.) Flore d'Afrique centrale (République démocratique du Congo - Rwanda - Burundi), nouvelle série, Spermatophyta. Meise, Jardin botanique de Meise. [version PDF, 10.5281/zenodo.5652952] [19 pages][pdf][fr][Congo]

324 Beentje H.J. (2014). Restionaceae. In : Sosef M.S.M. (éd.) Flore d'Afrique centrale (République démocratique du Congo - Rwanda - Burundi), nouvelle série, Spermatophyta. Meise, Jardin botanique de Meise. [version PDF, 10.5281/zenodo.5652974] [17 pages][pdf][fr][Congo]

325 Geerinck D.J.L. (2015). Smilacaceae. In : Sosef M.S.M. (éd.) Flore d'Afrique centrale (République démocratique du Congo - Rwanda - Burundi), nouvelle série, Spermatophyta. Meise, Jardin botanique de Meise. [version PDF, 10.5281/zenodo.5653167] [15 pages][pdf][fr][Congo]

326 Geerinck D.J.L. & Malaisse F.P. (2017). Tecophilaeaceae. In : Sosef M.S.M. (éd.) Flore d'Afrique centrale (République démocratique du Congo - Rwanda - Burundi), nouvelle série, Spermatophyta. Meise, Jardin botanique de Meise. [version PDF, 10.5281/zenodo.5653227] [19 pages][pdf][fr][Congo]

327 Leal M.E. (2017). Tropaeolaceae. In : Sosef M.S.M. (éd.) Flore d'Afrique centrale

(République démocratique du Congo - Rwanda - Burundi), nouvelle série, Spermatophyta. Meise, Jardin botanique de Meise. [version PDF, 10.5281/zenodo.5653276] [15 pages][pdf][fr][Congo]

328 Sosef M.S.M. (2017). Typhaceae. In : Sosef M.S.M. (éd.) Flore d'Afrique centrale (République démocratique du Congo - Rwanda - Burundi), nouvelle série, Spermatophyta. Meise, Jardin botanique de Meise. [version PDF, 10.5281/zenodo.5653345] [15 pages][pdf][fr][Congo]

329 Meerts P. (2020). Amaryllidaceae, sous-fam. Allioideae. In : Sosef M.S.M. (éd.) Flore d'Afrique centrale (République démocratique du Congo - Rwanda - Burundi), nouvelle série, Spermatophyta. Meise, Jardin botanique de Meise. [version PDF, 10.5281/zenodo.8163134] [19 pages][pdf][fr][Congo]

330 Beentje H.J. (2021) Flore d'Afrique Centrale (République démocratique du Congo – Rwanda – Burundi) nouvelle série SPERMATOPHYTA APOCYNACEAE Meise, Jardin botanique de Meise. ISSN 0779-116X [326 pages][pdf][fr][Congo]

331 Larridon I., M. Reynders et P. Thery (2020) Cyperaceae, Introduction, Tribu I. Hypolytreae. Flore d'Afrique centrale, nouvelle série. M.S.M. Sosef, rédaction – Meise, Jardin botanique de Meise, ISBN 978382361779-2 [62 pages][pdf][fr][Congo]

332 Hills R. & P. Wilkin (2021) Dioscoreaceae. – Flore d'Afrique centrale, nouvelle série, Spermatophyta. M.S.M. Sosef, rédaction – Meise, Jardin botanique de Meise, ISBN 978382361789-1 [62 pages][pdf][fr][Congo]

333 Fischer E. (2021) Gesneriaceae. – Flore d'Afrique centrale, nouvelle série, Spermatophyta. M.S.M. Sosef, rédaction – Meise, Jardin botanique de Meise, ISBN 9783823617945 [46 pages][pdf][fr][Congo]

334 Paton A., P. Meerts (2020) Labiatae, Introduction, Sous-familles I. Viticoideae et II. Premnoideae. – Flore d'Afrique centrale, nouvelle série. M.S.M. Sosef, rédaction – Meise, Jardin botanique de Meise, ISBN 978382361781-5 [70 pages][pdf][fr][Congo]

335 Luzolawo P.M., C.L. Ayingweu, S.M. Kamga, F.W. Stauffer (2020) Palmae, – Flore d'Afrique centrale, nouvelle série. M.S.M. Sosef, rédaction – Meise, Jardin botanique de Meise, ISBN 978382361778-5 [86 pages][pdf][fr][Congo]

336 Ilunga wa Ilunga E. & P. Meerts (2020) Phyllanthaceae. – Flore d'Afrique centrale, nouvelle série, Spermatophyta. M.S.M. Sosef, rédaction – Meise, Jardin botanique de Meise, ISBN 978382361787-7 [166 pages][pdf][fr][Congo]

337 Robbrecht E., P. De Block, S. Dessein & O. Lachenaud (2020) Rubiaceae,. – Flore d'Afrique centrale, nouvelle série, Spermatophyta. M.S.M.Sosef, rédaction – Meise, Jardin botanique de Meise, ISBN 9783823617761 [122 pages][pdf][fr][Congo]

338 De Block P. (2020) Rubiaceae Tribu V. Ixoreae. – Flore d'Afrique centrale, nouvelle série. M.S.M. Sosef, rédaction – Meise, Jardin botanique de Meise, ISBN 9783823617754 [42 pages][pdf][fr][Congo]

339 Ntore S. & O. Lachenaud (2020) Rubiaceae, Tribu XVI. Colletoecemateae à XVIII. Lasiantheae. – Flore d'Afrique centrale, nouvelle série. M.S.M. Sosef, rédaction – Meise, Jardin botanique de Meise, ISBN 9783823617778 [90 pages][pdf][fr][Congo]

340 Bikandu B., F. Lukoki, J.P. Habari, S. Ntore, M.S.M. Sosef (2020) Solanaceae, – Flore d'Afrique centrale, nouvelle série. M.S.M. Sosef, rédaction – Meise, Jardin botanique de Meise, ISBN 978382361780-8 [166 pages][pdf][fr][Congo]

341 Bizzarri M.P. (1985). Selaginellaceae. In : Bamps P. (éd.) Flore d'Afrique centrale (Zaïre – Rwanda – Burundi), Pteridophyta. Meise, Jardin botanique national de Belgique. [version PDF, 10.5281/zenodo.4694459] [61 pages][pdf][fr][Congo]

342 Mwanga Mwanga I.J.-C., Sosef M.S.M., Simões A.R.G. (2022). Convolvulaceae. Dans : Sosef M.S.M. (éd.) Flore d'Afrique centrale (République démocratique du Congo - Rwanda - Burundi), nouvelle série, Spermatophyta. Meise, Jardin botanique de Meise. [version PDF, 10.5281/zenodo.14697492] [259 pages][pdf][fr][Congo]

343 De Wildeman Em. (1903-1905) Etudes de systématique et de géographie botanique sur la Flore du Bas- et du Moyen-Congo, Etat Indépendant du congo , Annales du Musée du Congo publié par ordre du secrétaire d'Etat, Botanique - Série V. Vol. I - Fasc. I. Bruxelles [516 pages][pdf][fr][Congo]

344 Gray Asa, (1868) Manual of the Botany of The Northern United States, including the district East of the Mississippi and North of North Carolina and Tennessee, arranged according to the Natural System, Publisher: New York: Ivison , Phinnney, Blakeman & Co. Chicago: S.C. Criggs & Co. [754 pages][pdf][en][USA]

345 Rattan, Volney (1879) A Popular California Flora or Manual of Botany for Beginners containing Descriptions of Exogenous Plants growing in Central California, Westward to the Ocean. publisher San Francisco: A.L. Bancroft and Company. [118 pages][pdf][en][USA]

346 Coulter John M. (1885) Manual of the Botany. (Phaenogamia and pteridophyta) of the Rocky Mountain Region, from New Mexico to the British Boundary. NewYork Cincinnati Chicago American Book Company. [524 pages][pdf][en][USA]

347 Rattan, Volney (1896) A Popular California Flora or Manual of Botany for Beginners to which is added an Analytical Key to West Coast Botany. Containing brief Descriptions of over 1600 species of Pacific Coast Plants. publisher San Francisco: The Whitaker & Ray Co. [290 pages][pdf][en][USA]

348 Rattan, Volney (1880) A Popular California Flora or Manual of Botany for Beginners . Containing Descriptions of Flowering Plants growing in Central California, and Westward to the Ocean, with Illustrated Introductory Lessons. publisher San Francisco: A.L. Bancroft and Company. [162 pages][pdf][en][USA]

349 Robinson B.L. and Fernald M.L. (1908) Gray's New Manual of Botany. A Handbook of the Flowering Plants and Ferns of the Central and Northeastern United States and Adjacent Canada. Publisher: New York Cincinnati Chicago American Book Company. [938 pages][pdf][en][USA]

350 Peirce George J., Keck David D., Constance Lincoln, Epling Carl, Henderson L.F. (1935) Madrono A West American Journal of Botany. Volume III, Number 1. publisher: California Botanical Society [434 pages][pdf][en][USA]

351 Coulter John M. and Nelson Aven (1909) New Manual of Botany of the Central Rocky Mountains (vascular plants). New Cincinnati Chicago American Book Company. [668 pages][pdf][en][USA]

352 Gray Asa, (1863) Manual of the Botany of The Northern United States, including Virginia, Kentucky and All East of the Mississippi. Publisher: New York: Ivison , Phinnney, Blakeman & Co. Chicago: S.C. Criggs & Co. [908 pages][pdf][en][USA]

353 Sullivant William S. (1856) The Musci and Hepaticae of the United States, East of the Mississippi River, contributed to the second edition of the Gray's Manual of Botany.

Publisher: New York: George P. Putnam & Co. [129 pages][pdf][en][USA]
354 Penhallow D.P. (1899) A Manual of the More Common Herbaceous Plants of Quebec and Ontario. For use in connection with Groom's Botany (adapted from Gray' Manual). Publisher: Toronto: The Copp, Clark, Company, Limited. [225 pages][pdf][en][USA]
355 Gray Asa, (1848) Manual of the Botany of The Northern United States,From New England to Wisconsin and South to Ohio and and Liverworts by Wm. S. Sullivant). Publisher: Boston & Cambridge: JAmes Munroe and Company, London: John Chapman [784 pages][pdf][en][USA]
356 Small John K. (1913) Flora of the Southeastern United States being Descriptions of the seed-plants, Ferns and Ferns-Allies growing Naturally in North Carolina, South Carolina, Georgia, Florida, Tennessee, Alabama, Mississippi, Arkansas, Louisiana, and in Oklahoma and Texas East of the One Hundredth Meridian. Publiser: New York published by the author [1418 pages][pdf][en][USA]
357 Havard V. (1885) report of the Flora of Western and Northern Texas, Proceedings of the United States National Museum Volume VIII No 29 Washington D.C.. [98 pages][pdf][en][USA]
358 Chapman A.W. (1883) Flora of the Southern United States : containing an abridged description of the Flowering Plants and Ferns of Tennessee, North and South Carolina, Georgia, Alabama, Mississippi, and Florida: arranged according to the Natural System. Publisher: New York: Ivison, Blakeman, Taylor & Co. [748 pages][pdf][en][USA]
359 Rydberg P.A. (1900) Catalogue of the Flora of Montana and the Yellowstone National Park. New York Botanical Garden Volume 1. [518 pages][pdf][en][USA]
360 Heil, Kenneth D. , Steve L. O'Kane, Jr. Linda Mary Reeves Arnold Clifford (2013) Flora of the Four Corners Region Vascular Plants of the San Juan River Drainage Arizona, Colorado, New Mexico, and Utah. Monographs in Systematic Botany from the Missouri Botanical Garden, Volume 124. ISBN 978-1-930723-84-9 [1120 pages][pdf][en][USA]
361 John J. Furlow and Richard S. Mitchell (1990) Betulaceae through Cactaceae of New York State. publisher: Bulletin No. 476 New York State Museum [104 pages][pdf][en][USA]
362 Mitchell Richard S. (1993) Portulacaceae through Caryophyllaceae of New York State. publisher: BULLETIN NO. 486 New York State Museum [136 pages][pdf][en][USA]
363 Rouy G. and Foucaud J. (1893) Flore de France ou description des plantes qui croissent spontanément en France, en Corse, et en Alsace-Lorraine, tome I. Publisher: Société des Sciences Naturelles de la Charente Inférieure. [332 pages][pdf][fr][France]
364 Rouy G. and Foucaud J. (1895) Flore de France ou description des plantes qui croissent spontanément en France, en Corse, et en Alsace-Lorraine, tome II. Publisher: Société des Sciences Naturelles de la Charente Inférieure. [361 pages][pdf][fr][France]
365 Rouy G. and Foucaud J. (1895) Flore de France ou description des plantes qui croissent spontanément en France, en Corse, et en Alsace-Lorraine, tome III. Publisher: Société des Sciences Naturelles de la Charente Inférieure. [386 pages][pdf][fr][France]
366 Rouy G. and Foucaud J. (1897) Flore de France ou description des plantes qui croissent spontanément en France, en Corse, et en Alsace-Lorraine, tome IV. Publisher: Société des Sciences Naturelles de la Charente Inférieure. [319 pages][pdf][fr][France]
367 Rouy G. (1899) Flore de France ou description des plantes qui croissent spontanément en France, en Corse, et en Alsace-Lorraine, tome V. Publisher: Société des Sciences Naturelles

de la Charente Inférieure. [319 pages][pdf][fr][France]
368 Rouy G. and Camus E.-G. (1900) Flore de France ou description des plantes qui croissent spontanément en France, en Corse, et en Alsace-Lorraine, tome VI. Publisher: Société des Sciences Naturelles de la Charente Inférieure. [497 pages][pdf][fr][France]
369 Rouy G. and Camus E.-G. (1901) Flore de France ou description des plantes qui croissent spontanément en France, en Corse, et en Alsace-Lorraine, tome VII. Publisher: Société des Sciences Naturelles de la Charente Inférieure. [446 pages][pdf][fr][France]
370 Rouy G. (1903) Flore de France ou description des plantes qui croissent spontanément en France, en Corse, et en Alsace-Lorraine, tome VIII. Publisher: Société des Sciences Naturelles de la Charente Inférieure. [413 pages][pdf][fr][France]
371 Rouy G. (1905) Flore de France ou description des plantes qui croissent spontanément en France, en Corse, et en Alsace-Lorraine, tome IX. Publisher: Société des Sciences Naturelles de la Charente Inférieure. [496 pages][pdf][fr][France]
372 Rouy G. (1908) Flore de France ou description des plantes qui croissent spontanément en France, en Corse, et en Alsace-Lorraine, tome X. Publisher: Société des Sciences Naturelles de la Charente Inférieure. [408 pages][pdf][fr][France]
373 Rouy G. (1909) Flore de France ou description des plantes qui croissent spontanément en France, en Corse, et en Alsace-Lorraine, tome XI. Publisher: Société des Sciences Naturelles de la Charente Inférieure. [435 pages][pdf][fr][France]
374 Rouy G. (1910) Flore de France ou description des plantes qui croissent spontanément en France, en Corse, et en Alsace-Lorraine, tome XII. Publisher: Société des Sciences Naturelles de la Charente Inférieure. [511 pages][pdf][fr][France]
375 Rouy G. (1912) Flore de France ou description des plantes qui croissent spontanément en France, en Corse, et en Alsace-Lorraine, tome XIII. Publisher: Société des Sciences Naturelles de la Charente Inférieure. [556 pages][pdf][fr][France]
376 Rouy G. (1913) Flore de France ou description des plantes qui croissent spontanément en France, en Corse, et en Alsace-Lorraine, tome XIV. Publisher: Société des Sciences Naturelles de la Charente Inférieure. [572 pages][pdf][fr][France]
377 Rouy G. (1927) Flore de France ou description des plantes qui croissent spontanément en France, en Corse, et en Alsace-Lorraine, conspectus de la flore de France. Publisher: Paul Lechevalier, Paris. [337 pages][pdf][fr][France]
378 Lloyd, James (1897) Flore de l'Ouest de la France ou description des plantes qui croissent spontanément dans les départements de: Charentes-Inférieure, Deux-Sèvres, Vendée, Loire-Inférieure, Morbihan, Finistère, Côtes-du-Nord, Ile-et-Vilaine. Publisher: M. Emile Gadeceau, Nantes [606 pages][pdf][fr][France]
379 Aubréville André (1961) Flore du Gabon. volume 1, Famille des Sapotacées. Publisher: Muséum National d'Histoire Naturelle, Paris. [168 pages][pdf][fr][Gabon]
380 Aubréville André, Hallé N. (1961) Flore du Gabon. volume 2, Famille des Sterculiacées. Publisher: Muséum National d'Histoire Naturelle, Paris. [154 pages][pdf][fr][Gabon]
381 Aubréville André (1962) Flore du Gabon. volume 3, Famille des Irvingiacées, Simaroubacées, Burséracées. Publisher: Muséum National d'Histoire Naturelle, Paris. [106 pages][pdf][fr][Gabon]
382 Aubréville André, Hallé N. (1962) Flore du Gabon. volume 4, Famille des Mélianthacées, Balsaminacées, Rhamnacées. Publisher: Muséum National d'Histoire Naturelle, Paris. [78

pages][pdf][fr][Gabon]
383 Aubréville André, Koechlin J. (1962) Flore du Gabon. volume 5, Famille des Graminées. Publisher: Muséum National d'Histoire Naturelle, Paris. [296 pages][pdf][fr][Gabon]
384 Letouzey René (1963) Flore du Gabon. volume 6, Famille des Rutacées, Zygophyllacées, Balanitacées. Publisher: Muséum National d'Histoire Naturelle, Paris. [126 pages][pdf][fr][Gabon]
385 Cavaco A. (1963) Flore du Gabon. volume 7, Famille des Polygonacées, Chénopodiacées, Amaranthacées, Nyctaginacées, Phytolaccacées, Aizoacées, Portulacacées, Caryophyllacées. Publisher: Muséum National d'Histoire Naturelle, Paris. [80 pages][pdf][fr][Gabon]
386 Tardieu-Blot M.L. (1964) Flore du Gabon. volume 8, Famille des Ptéridophytes. Publisher: Muséum National d'Histoire Naturelle, Paris. [231 pages][pdf][fr][Gabon]
387 Koechlin J. (1964) Flore du Gabon. volume 9, Scitaminales: Musacées, Strélitziacées, Zingibéracées, Cannacées, Marantacées. Publisher: Muséum National d'Histoire Naturelle, Paris. [178 pages][pdf][fr][Gabon]
388 Fouilloy R. (1964) Flore du Gabon. volume 10, Lauracées, Myristicacées, Monimiacées. Publisher: Muséum National d'Histoire Naturelle, Paris. [120 pages][pdf][fr][Gabon]
389 Amshoff G.J.H., Aymonin G.-G. (1966) Flore du Gabon. volume 11, Myrtacées, Thyméléacées. Publisher: Muséum National d'Histoire Naturelle, Paris. [120 pages][pdf][fr][Gabon]
390 Hallé N. (1966) Flore du Gabon. volume 12, Famille des Rubiacées (1ère partie). Publisher: Muséum National d'Histoire Naturelle, Paris. [284 pages][pdf][fr][Gabon]
391 Heine H. (1966) Flore du Gabon. volume 13, Acanthacées. Publisher: Muséum National d'Histoire Naturelle, Paris. [254 pages][pdf][fr][Gabon]
392 Descoings B., Hallé N., Raynal J. (1968) Flore du Gabon. volume 14, Vitacées et Leeacées. Publisher: Muséum National d'Histoire Naturelle, Paris. [128 pages][pdf][fr][Gabon]
393 Aubréville André (1968) Flore du Gabon. volume 15, LÉGUMINEUSES - CAESALPINIOIDÉES. Publisher: Muséum National d'Histoire Naturelle, Paris. [367 pages][pdf][fr][Gabon]
394 Le Thomas Annick (1969) Flore du Gabon. volume 16, Annonacées. Publisher: Muséum National d'Histoire Naturelle, Paris. [379 pages][pdf][fr][Gabon]
395 Hallé N. (1966) Flore du Gabon. volume 17, Famille des Rubiacées (2e partie). Publisher: Muséum National d'Histoire Naturelle, Paris. [339 pages][pdf][fr][Gabon]
396 Letouzey René and White Franck (1970) Flore du Gabon. volume 18, Famille des Ebénacées. Publisher: Muséum National d'Histoire Naturelle, Paris. [194 pages][pdf][fr][Gabon]
397 Leeuwenberg A.J.M. (1972) Flore du Gabon. volume 19, Famille des Loganiacées. Publisher: Muséum National d'Histoire Naturelle, Paris. [158 pages][pdf][fr][Gabon]
398 Villiers Jean-F. (1973) Flore du Gabon. volume 20, Icacinacées, Olacacées, Pentadiplandracées, Opiliacées, Octoknémacées (avec compléments concernant des espèces camerounaises). Publisher: Muséum National d'Histoire Naturelle, Paris. [204 pages][pdf][fr][Gabon]
399 Badré F. and Lawalrée A. (1973) Flore du Gabon. volume 21, Malpighiacées, Nectaropétalacées, Linacées, Lépidobotryacées, Cténolophonacées, Humiriacées,

Erythroxylacées, Ixonanthacées. Publisher: Muséum National d'Histoire Naturelle, Paris. [76 pages][pdf][fr][Gabon]
400 Villiers Jean-F. (1973) Flore du Gabon. volume 22, Célastracées, Pandacées, Bombacacées, Cannabacées, Bixacées, Avicenniacées. Publisher: Muséum National d'Histoire Naturelle, Paris. [76 pages][pdf][fr][Gabon]
401 Fouilloy Roger and Hallé Nicolas (1973) Flore du Gabon. volume 23, Sapindacées (avec compléments concernant les espèces camerounaises). Publisher: Muséum National d'Histoire Naturelle, Paris. [208 pages][pdf][fr][Gabon]
402 Letouzey René and White Franck (1978) Flore du Gabon. volume 24, Scytopétalacées. Publisher: Muséum National d'Histoire Naturelle, Paris. ISBN 2-85654-154-2 [206 pages][pdf][fr][Gabon]
403 Jacques-Félix H. (1983) Flore du Gabon. volume 25, Mélastomatacées. Publisher: Muséum National d'Histoire Naturelle, Paris. ISBN 2-85654-166-4 [172 pages][pdf][fr][Gabon]
404 Berg C.C., M.E.E. Hijman & J.C.A. Weerdenburg (1984) Flore du Gabon. volume 26, MORACÉES (incl. CECROPIACÉES). Publisher: Muséum National d'Histoire Naturelle, Paris. ISBN 2-85654-171-2 [280 pages][pdf][fr][Gabon]
405 Burtt B.L. and A.H. Gentry (1985) Flore du Gabon. volume 27, GESNÉRIACÉES, BIGNONIACÉES. Publisher: Muséum National d'Histoire Naturelle, Paris. ISBN 2-85654-174-7 [66 pages][pdf][fr][Gabon]
406 Huynh K.-L., I. Nordal, J.I. Iversen, and J.-F. VILLIERS (1986) Flore du Gabon. volume 28, PANDANACÉES, AMARYLLIDACÉES, HYPOXIDACÉES, FLAGELLARIACÉES. Publisher: Muséum National d'Histoire Naturelle, Paris. ISBN 2-85654-177-1 [64 pages][pdf][fr][Gabon]
407 Hallé N. (1986) Flore du Gabon. volume 29, CELASTRACEAE HIPPOCRATEOIDEAE. Publisher: Muséum National d'Histoire Naturelle, Paris. ISBN 2-85654-178-X [292 pages][pdf][fr][Gabon]
408 Kers L.E. and B. Jonsell B. (1987) Flore du Gabon. volume 30, CAPPARIDACEÆ, BRASSICACEÆ . Publisher: Muséum National d'Histoire Naturelle, Paris. ISBN 2-85654-181-X [88 pages][pdf][fr][Gabon]
409 Villiers J.-F. (1989) Flore du Gabon. volume 31, LEGUMINOSAE-MIMOSOIDEAE . Publisher: Muséum National d'Histoire Naturelle, Paris. ISBN 2-85654-185-2 [190 pages][pdf][fr][Gabon]
410 Breteler F.J. (1991) Flore du Gabon. volume 32, DICHAPETALACEAE . Publisher: Muséum National d'Histoire Naturelle, Paris. ISBN 2-85654-191-7 [225 pages][pdf][fr][Gabon]
411 Breteler F.J., C.C.H. Jongkind & R.H.M.J. Lemmens (1992) Flore du Gabon. volume 33, CONNARACEAE. Publisher: Muséum National d'Histoire Naturelle, Paris. ISBN 2-85654-193-3 [146 pages][pdf][fr][Gabon]
412 Hul S. (1995) Flore du Gabon. volume 34, FLACOURTIACEAE. Publisher: Muséum National d'Histoire Naturelle, Paris. ISBN 2-86654-201-8 [86 pages][pdf][fr][Gabon]
413 Jongkind C.C.H. (1999) Flore du Gabon. volume 35, COMBRETACEAE. Publisher: Muséum National d'Histoire Naturelle, Paris. ISBN 2-85654-204-2 [120 pages][pdf][fr][Gabon]
414 Szlachetko Dariusz L., Magdalena Sawicka and Marta Kras-Lapinska (2004) Flore du Gabon. volume 36, ORCHIDACEAE I. Publisher: Muséum National d'Histoire Naturelle, Paris. ISBN 2-85654-216-6 [236 pages][pdf][fr][Gabon]

415 Szlachetko Dariusz L., Magdalena Sawicka and Marta Kras-Lapinska (2004) Flore du Gabon. volume 37, ORCHIDACEAE II. Publisher: Muséum National d'Histoire Naturelle, Paris. ISBN 2-85654-217-4 [236 pages][pdf][fr][Gabon]

416 Potier de la Varde R. (1936) Mousses du Gabon. Extrait des Mémoires de la Société Nationale des Sciences Naturelles et Mathématiques de Cherbourg. Imprimerie R. Jacqueline, Saint-Lo. [272 pages][pdf][fr][Gabon]

417 Bentham George (1918) Handbook of The British Flora. A description of the flowering Plants and Ferns indigenous to, or naturalised in, The British Isles, for the use of beginners and amateurs. Publisher: London: L. Reeve & Co., LTD. [674 pages][pdf][en][UK]

418 Stewart, Samuel A. and Corry , Th.H. (1888) A Flora of the North-East of Ireland including the phanerogamia, the cryptogamia vascularia and the muscineae. Publisher: The Belfast Naturalists' Field Club, Cambridge: Macmillan & Bowes. [376 pages][pdf][en][UK]

419 Hooker J.D. (1875) The Flora of British India. Volume I. Ranunculaceae to Spindaceae. Publisher: L. Reeve & Co. [778 pages][pdf][en][India]

420 Hooker J.D. (1879) The Flora of British India. Volume II. SABIACEES TO CORNACEAE. Publisher: L. Reeve & Co. [812 pages][pdf][en][India]

421 Hooker J.D. (1882) The Flora of British India. Volume III. CAPRIFOLIACEES TO APOCYNACEES. Publisher: L. Reeve & Co. [716 pages][pdf][en][India]

422 Hooker J.D. (1885) The Flora of British India. Volume IV. ASCLEPIADEAE to AMARANTACEAE. Publisher: L. Reeve & Co. [716 pages][pdf][en][India]

423 Hooker J.D. (1890) The Flora of British India. Volume V. CHENOPODIACEAE TO ORCHIDEAE. Publisher: L. Reeve & Co. [914 pages][pdf][en][India]

424 Hooker J.D. (1894) The Flora of British India. Volume VI. ORCHIDEAE to CYPERACEAE. Publisher: L. Reeve & Co. [804 pages][pdf][en][India]

425 Hooker J.D. (1897) The Flora of British India. Volume VII. CYPERACEAE, GRAMINEAE AND GENERAL INDEX. Publisher: L. Reeve & Co. [860 pages][pdf][en][India]

426 Post, George R. (1896) Flora of Syria, Palestine and Sinai, from the Taurus to Ras Muhammad and from the Mediterranean Sea to the Syrian Desert. [930 pages][pdf][en][Israel]

427 Makino T. (1901) Observations on the Flora of Japan. Fasciculus 1. publisher: Botanical Magazine, Tokyo, Vol.XV. [494 pages][pdf][en][Israel]

428 Matevski Vlado, Andraž Čarni, Mitko Kostadinovski, Petra Košir, Urban Šilc, Igor Zelnik (2008) Flora and Vegetation of the Macedonian Steppe. Publisher: ZRC SAZU, Biološki inštitut Jovana Hadžija. ISBN 978-961-254-549-9 [98 pages][pdf][en][Macedonia]

429 Humbert H. and Tardieu-Blot M.-L. (1951) Flore de Madagascar et des Comores (plantes vasculaires). Familles: MARATTIACÉES, OPHIOGLOSSACÉES, HYMÉNOPHYLLACÉES, CYATHÉACÉES. Publisher: Museum National d'Histoire Naturelle, Paris. [9 pages][pdf][fr][Madagascar]

430 Renauld, Ferdinand (1897) Prodrome de la Flore Bryologique de Madagascar, des Mascareignes et des Comores. Publisher: Institut de France, imprimerie de Monaco [316 pages][pdf][fr][Madagascar]

431 Thériot, I. (1927) Sixième contribution à la Flore Bryologique de Madagascar. Publisher: Société Havraise d'etudes diverses (extrait du recueil de ses publications) [17 pages][pdf][fr][Madagascar]

432 Thériot, I. (1922) Deuxième contribution à la Flore Bryologique de Madagascar. Publisher: Société Havraise d'etudes diverses (extrait du recueil de ses publications) [25 pages][pdf][fr][Madagascar]

433 Thériot, I. (1923) Troisième contribution à la Flore Bryologique de Madagascar. Publisher: Société Havraise d'etudes diverses (extrait du recueil de ses publications) [27 pages][pdf][fr][Madagascar]

434 Thériot, I. (1924) Quatrième contribution à la Flore Bryologique de Madagascar. Publisher: Société Havraise d'etudes diverses (extrait du recueil de ses publications) [19 pages][pdf][fr][Madagascar]

435 Thériot, I. (1926) Cinquième contribution à la Flore Bryologique de Madagascar. Publisher: Société Havraise d'etudes diverses (extrait du recueil de ses publications) [33 pages][pdf][fr][Madagascar]

436 Thériot, I. (1930) Septième contribution à la Flore Bryologique de Madagascar. Publisher: Société Havraise d'etudes diverses (extrait du recueil de ses publications) [26 pages][pdf][fr][Madagascar]

437 Thériot, I. (1932) Huitième contribution à la Flore Bryologique de Madagascar. Publisher: Société Havraise d'etudes diverses (extrait du recueil de ses publications) [26 pages][pdf][fr][Madagascar]

438 Thériot, I. (1920) Huitième contribution à la Flore Bryologique de Madagascar. Publisher: Société Havraise d'etudes diverses (extrait du recueil de ses publications) [21 pages][pdf][fr][Madagascar]

439 Van Steenis C.G.G.J. and Holttum R.E. (1959-1982) Flora Malesiana. Series II Pteridophyta, Volume 1 part 1-5. Publisher: Martinus Nijhoff / DR W. Junk Publishers, The Hague / Boston / London. ISBN 90 247 2653 0 [656 pages][pdf][en][Indonesia]

440 Holttum R.E. (1991) Flora Malesiana. Series II Pteridophyta, Volume 2 part 1. Tectaria group. Publisher: under the auspices of Foundation Flora Malesiana. ISBN 90-71236-11-0 [140 pages][pdf][en][Indonesia]

441 Van Steenis C.G.G.J. (1948-1954) Flora Malesiana. Series I Spermatophyta, Volume 4. Aceraceae , Nyssaceae , Actinidiaceae, Philydraceae, Ancistrocladaceae ,Sarcospermaceae ,. ,Aponogetonaceae, Sphenocleaceae ,Burmanniaceae ,Stackhousiaceae. Publisher: NOORDHOFF-KOLFF N.V., BATAVIA. [888 pages][pdf][en][Indonesia]

442 Van Steenis C.G.G.J. , P. W. Leenhouts, Ding Hou, J. P. D. W. Payens, H. Sleumer, W. Vink, C. den Hartog, M. Jacobs, K. Bakker (1955-58) Flora Malesiana. Series I Spermatophyta, Volume 5., Betulaceae , Burseraceae ,Butomaceae ,Centrolepidaceae ,Connaraceae ,Dichapetalaceae,Erythroxylaceae ,Flacourtiaceae ,Goodeniaceae ,Haemodoraceae ,Hamamelidaceae,Hydrocharitaceae,Malpighiaceae ,Papaveraceae ,Pentaphylacaceae ,Pittosporaceae , Proteaceae , Restionaceae , Rhizophoraceae, Salicaceae , Scyphostegiaceae. Publisher: NOORDHOFF-KOLFF N.V., BATAVIA. [966 pages][pdf][en][Indonesia]

443 Van Steenis C.G.G.J. and Ding Hou (1960) Flora Malesiana. Series I Spermatophyta, Volume 6 part 1. THYMELAEACEAE (Publisher: NOORDHOFF-KOLFF N.V., BATAVIA. [160 pages][pdf][en][Indonesia]

444 Van Steenis C.G.G.J. and W.J.J.O. de Wilde (1962) Flora Malesiana. Series I Spermatophyta,

Volume 6 part 2. NAJADACEAE. Publisher: NOORDHOFF-KOLFF N.V., BATAVIA. [236 pages][pdf][en][Indonesia]
445 Van Steenis C.G.G.J. and Ding Hou (1964) Flora Malesiana. Series I Spermatophyta, Volume 6 part 3. CELASTRACEAE. Publisher: NOORDHOFF-KOLFF N.V., BATAVIA. [84 pages][pdf][en][Indonesia]
446 Van Steenis C.G.G.J. and H. Sleumer (1966) Flora Malesiana. Series I Spermatophyta, Volume 6 part 4. ERICACEAE. Publisher: NOORDHOFF-KOLFF N.V., BATAVIA. [204 pages][pdf][en][Indonesia]
447 Van Steenis C.G.G.J. and H. Sleumer (1967) Flora Malesiana. Series I Spermatophyta, Volume 6 part 5. ERICACEAE. Publisher: NOORDHOFF-KOLFF N.V., BATAVIA. [252 pages][pdf][en][Indonesia]
448 Van Steenis C.G.G.J. and H. Sleumer (1972) Flora Malesiana. Series I Spermatophyta, Volume 6 part 6. Taxonomical revisions. Publisher: WOLTERS-NOORDHOFF PUBLISHING, GRONINGEN. ISBN 9001 31822 3 [136 pages][pdf][en][Indonesia]
449 Van Steenis C.G.G.J. and H. Sleumer (1971) Flora Malesiana. Series I Spermatophyta, Volume 7 part 1. ICACINACEAE. Publisher: WOLTERS-NOORDHOFF PUBLISHING, GRONINGEN. ISBN 90 01 31813 4 [268 pages][pdf][en][Indonesia]
450 Van Steenis C.G.G.J. and E. Soepadmo (1972) Flora Malesiana. Series I Spermatophyta, Volume 7 part 2. FAGACEAE. Publisher: OLTERS-NOORDHOFF PUBLISHING, GRONINGEN. ISBN 90 01 31814 2 [180 pages][pdf][en][Indonesia]
451 Van Steenis C.G.G.J. and J. H. Kern (1974) Flora Malesiana. Series I Spermatophyta, Volume 7 part 3. CYPERACEAE. Publisher: NOORDHOFF INTERNATIONAL PUBLISHING, LEYDEN,. ISBN 90 286 0443 x [324 pages][pdf][en][Indonesia]
452 Van Steenis C.G.G.J. and J. H. Kern (1972-1976) Flora Malesiana. Series I Spermatophyta, Volume 7 part 4. Taxonomical revisions. Publisher: NOORDHOFF INTERNATIONAL PUBLISHING, LEYDEN. ISBN 90 286 0615 7 [148 pages][pdf][en][Indonesia]
453 VAN STEENIS-KRUSEMAN M. J. (1974) Flora Malesiana. Series I Spermatophyta, Volume 8 part 1. Cyclopaedia of Collectors - Supplement 2 Hypericaceae. Publisher: NOORDHOFF INTERNATIONAL PUBLISHING, LEYDEN. ISBN 90 286 0164 3 [156 pages][pdf][en][Indonesia]
454 Van Steenis C.G.G.J. and E. Soepadmo (1977) Flora Malesiana. Series I Spermatophyta, Volume 8 part 2. ULMACEAE. Publisher: SIJTHOFF & NOORDHOFF INTERNATIONAL PUBLISHERS. ISBN 90 286 0573 I [276 pages][pdf][en][Indonesia]
455 Van Steenis C.G.G.J. (1978) Flora Malesiana. Series I Spermatophyta, Volume 8 part 3. Taxonomical revisions. Publisher: SISTHOFF & NOORDHOFF INTERNATIONAL PUBLISHERS. ISBN 90 286 0178 3 [302 pages][pdf][en][Indonesia]
456 Van Steenis C.G.G.J. and J. H. Kern (1983) Flora Malesiana. Series I Spermatophyta, Volume 9. Taxonomical revisions. Publisher: Martinus Nijhoff Publishers, The Hague. ISBN 90-247-2780-4 [656 pages][pdf][en][Indonesia]
457 Van Steenis C.G.G.J. and W. R. Philipson (1979) Flora Malesiana. Series I Spermatophyta, Volume 9 part 1. ARALIACEAE. Publisher: SIJSTHOFF & NOORDHOFF INTERNATIONAL PUBLISHERS. ISBN 90 286 0629 7 [240 pages][pdf][en][Indonesia]
458 Van Steenis C.G.G.J. and J. H. Kern (1989) Flora Malesiana. Series I Spermatophyta, Volume 10. Taxonomical revisions. Publisher: Published by Kluwer Academic Publishers, Dordrecht.

ISBN 0-7923-0421-7 [808 pages][pdf][en][Indonesia]
459 Van Steenis C.G.G.J. and H. Sleumer (1984) Flora Malesiana. Series I Spermatophyta, Volume 10 part 1. OLACACEAE. Publisher: MARTINUS NIJHOFF PUBLISHERS THE HAGUE/BOSTON/LONDON. ISBN 90 247 2887 8 [128 pages][pdf][en][Indonesia]
460 Van Steenis C.G.G.J. and B. Verdcourt (1986) Flora Malesiana. Series I Spermatophyta, Volume 10 part 1. CHLORANTHACEAE. Publisher: MARTINUS NIJHOFF PUBLISHERS DORDRECHT. ISBN-90-247-3280-8 [218 pages][pdf][en][Indonesia]
461 Van Steenis C.G.G.J. and D.J. de Laubenfels (1988) Flora Malesiana. Series I Spermatophyta, Volume 10 part 3. CONIFERALES. Publisher: KLUWER ACADEMIC PUBLISHERS DORDRECHT. ISBN-90-247-3736-2 [304 pages][pdf][en][Indonesia]
461 Dr. W. J. J. O. DE WILDE (1988) Flora Malesiana. Series I Spermatophyta, Volume 10 part 4. CONIFERALES. Publisher: KLUWER ACADEMIC PUBLISHERS DORDRECHT. ISBN 0-7923-0422-5 [164 pages][pdf][en][Indonesia]
462 Nielsen LC. (1992) Flora Malesiana. Series I Spermatophyta, Volume 11 part 1. Mimosaceae (Leguminosae—Mimosoideae). Publisher: KLUWER ACADEMIC PUBLISHERS DORDRECHT. ISBN 90-71236-16-1 [238 pages][pdf][en][Indonesia]
463 Nielsen LC., D.J.L. Geerinck, J.R.M. Buijsen, B.E.E. Duyfjes, B.E.E. Duyfjes,B.E.E. Duyfjes (1993) Flora Malesiana. Series I Spermatophyta, Volume 11 part 2. Rosaceae, Amaryllidaceae, Alliaceae, Coriariaceae, Pentastemonaceae, Stemonaceae. Publisher: Foundation Flora Malesiana. ISBN 90-71236-19-6 [200 pages][pdf][en][Indonesia]
464 Adema, F. , P.W. Leenhouts P.C. van Welzen (1994) Flora Malesiana. Series I Spermatophyta, Volume 11 part 3. Sapindaceae. Publisher: Foundation Flora Malesiana. ISBN 90-71236-21-8 [352 pages][pdf][en][Indonesia]
465 Mabberley, D.J. , C.M. Pannell A.M. Sing (1995) Flora Malesiana. Series I Spermatophyta, Volume 12 part 1. Meliaceae. Publisher: Foundation Flora Malesiana. ISBN 90-71236-26-9 [409 pages][pdf][en][Indonesia]
466 Kalkman C. , D.J.L. Geerinck , J.R.M. Buijsen, B.E.E. Duyfjes, Ding Hou, K. Larsen & S.S. Larsen, J.E. Laferriére (1996) Flora Malesiana. Series I Spermatophyta, Volume 12 part 2. Amaryllidaceae , Alliaceae , Coriariaceae , Pentastemonaceae, Stemonaceae, Rosaceae, Caesalpiniaceae, Geitonoplesiaceae, Hernandiaceae, Lowiaceae. Publisher: Foundation Flora Malesiana. ISBN 90-71236-29-3 [384 pages][pdf][en][Indonesia]
467 Meijer W., H. Riedl, T.-C. Huang, R.M.K. Saunders , B.A. Barlow (1997) Flora Malesiana. Series I Seed Plants, Volume 13. Rafflesiaceae, Boraginaceae, Daphniphyllaceae, Illiciaceae & Schisandraceae, Loranthaceae & Viscaceae. Publisher: Foundation Flora Malesiana. ISBN 90 -71236 -33-1 [464 pages][pdf][en][Indonesia]
468 de Wilde, W.J.J.O. (1997) Flora Malesiana. Series I Seed Plants, Volume 14. Myristicaceae. Publisher: Nationaal Herbarium Nederland, Universiteit Leiden branch, The Netherlands, under the auspices of Foundation Flora Malesiana. ISBN 90-71236-47-1 [650 pages][pdf][en][Indonesia]
469 Cheek, Martin and Matthew Jebb (1997) Flora Malesiana. Series I Seed Plants, Volume 15. Nepenthaceae. Publisher: Nationaal Herbarium Nederland, Universiteit Leiden branch, The Netherlands, under the auspices of Foundation Flora Malesiana. ISBN 90-71236-49-8 [163 pages][pdf][en][Indonesia]
470 Berg C.C., E.J.H. Corner and F.M. Jarrett (2006) Flora Malesiana. Series I Seed Plants,

Volume 17 part 1. Moraceae. Publisher: Nationaal Herbarium Nederland, Universiteit Leiden branch, The Netherlands, under the auspices of Foundation Flora Malesiana. [147 pages][pdf][en][Indonesia]

471 Berg C.C. and E.J.H. Corner (2005) Flora Malesiana. Series I Seed Plants, Volume 17 part 2. MORACEAE: FICEAE. Publisher: Nationaal Herbarium Nederland, Universiteit Leiden branch, The Netherlands, under the auspices of Foundation Flora Malesiana. [702 pages][pdf][en][Indonesia]

472 Middleton David J. (2007) Flora Malesiana. Series I Seed Plants, Volume 18. APOCYNACEAE. Publisher: Nationaal Herbarium Nederland, Universiteit Leiden branch, The Netherlands, under the auspices of Foundation Flora Malesiana. [452 pages][pdf][en][Indonesia]

473 de Wilde, W.J.J.O. and Brigitta EE Duyfjes (2010) Flora Malesiana. Series I Seed Plants, Volume: 19. CUCURBITACEAE. Publisher: Nationaal Herbarium Nederland, Universiteit Leiden branch, The Netherlands, under the auspices of Foundation Flora Malesiana. [333 pages][pdf][en][Indonesia]

474 van Welzen P.C. (2011) Flora Malesiana. Series I Seed Plants, Volume 20. PANDACEAE. Publisher: Nationaal Herbarium Nederland, Universiteit Leiden branch, The Netherlands, under the auspices of Foundation Flora Malesiana. [30 pages][pdf][en][Indonesia]

475 Prance G.T. and E.K. Kartawinata (2013) Flora Malesiana. Series I Seed Plants, Volume 21. LECYTHIDACEAE. Publisher: Nationaal Herbarium Nederland, Universiteit Leiden branch, The Netherlands, under the auspices of Foundation Flora Malesiana. [118 pages][pdf][en][Indonesia]

476 de Wilde, W.J.J.O. and B.E.E. Duyfjes (2016) Flora Malesiana. Series I Seed Plants, Volume 22. Lythraceae. Publisher: Nationaal Herbarium Nederland, Universiteit Leiden branch, The Netherlands, under the auspices of Foundation Flora Malesiana. ISBN 978-90-6519-016-1 [70 pages][pdf][en][Indonesia]

477 Espejo-Serna, Adolfo , Ana Rosa López-Ferrari & Ivón Ramírez-Morillo (2005) Flora de Veracruz, Fasciculo 136. publisher: Xalapa : Instituto de Ecología, A. C. ; Riverside : University of California. ISSN 0187-425X [315 pages][pdf][en][Mexico]

478 Simmons, M. P. - Mabberley, D. J. - Kok, R. P. J. de (2004) Flore de la Nouvelle-Calédonie et dépendances. Hippocrateaceae, Labitae, Vitaceae., volume 25 . Publisher: Muséum National d'Histoire Naturelle, Paris. ISBN 2-85654-219-0 [168 pages][pdf][fr][NewCaledonia]

479 Tirel, Christiane - Veillon, Jean-Marie (2002) Flore de la Nouvelle-Calédonie et dépendances. Pittosporaceae, volume 24. Publisher: Muséum National d'Histoire Naturelle, Paris. ISBN 2-85654-213-1 [180 pages][pdf][fr][NewCaledonia]

480 Dawson, John Wyndham (1999) Flore de la Nouvelle-Calédonie et dépendances. Myrtaceae : Myrtoideae I : Syzygium, volume 23 . Publisher: Muséum National d'Histoire Naturelle, Paris. ISBN 2-85654-215-8 [144 pages][pdf][fr][NewCaledonia]

481 Forman, Leonard - Green, Peter S. (1998) Flore de la Nouvelle-Calédonie et dépendances. Menispermaceae, Oleaceae, Passifloraceae, volume 22. Publisher: Muséum National d'Histoire Naturelle, Paris. ISBN 2-85654-209-3 [92 pages][pdf][fr][NewCaledonia]

482 Jérémie, Joël - Hoff, M. - Jonsell, B. (1997) Flore de la Nouvelle-Calédonie et dépendances. Sphenostemonaceae, Anacardiaceae, Cruciferae, volume 21. Publisher:

Muséum National d'Histoire Naturelle, Paris. ISBN 2-85654-205-0 [124 pages][pdf][fr][NewCaledonia]

483 Müller, I.H. - Barlow, B.A. - Tirel, C. - Jérémie, J. (1996) Flore de la Nouvelle-Calédonie et dépendances. Celastraceae, Loranthaceae, Viscaceae, Alseuosmiaceae, Paracryphiaceae, Tiliaceae, volume 20. Publisher: Muséum National d'Histoire Naturelle, Paris. ISBN 2-85654-203-4 [148 pages][pdf][fr][NewCaledonia]

484 White, Frank - Vink, Wim (1993) Flore de la Nouvelle-Calédonie et dépendances. Ebenaceae, Winteraceae, volume 19. Publisher: Muséum National d'Histoire Naturelle, Paris. ISBN 2-85654-196-8 [176 pages][pdf][fr][NewCaledonia]

485 Dawson, John Wyndham and Tirel, Christiane (1992) Flore de la Nouvelle-Calédonie et dépendances. Myrtaceae - Leptospermoideae, volume 18. Publisher: Muséum National d'Histoire Naturelle, Paris. ISBN 2-85654-195-X [252 pages][pdf][fr][NewCaledonia]

486 McPherson, Gordon - Schmid, Maurice (1991) Flore de la Nouvelle-Calédonie et dépendances. Euphorbiaceae - Phyllanthoideae : Antidesma, Bischofia, Breynia, Cleistanthus, Drypetes, Glochidion, Phyllanthus, volume 17. Publisher: Muséum National d'Histoire Naturelle, Paris. ISBN 2-85654-190-9 [332 pages][pdf][fr][NewCaledonia]

487 Veillon, Jean-Marie - Goldblatt, P. - Müller, I. H. (1990) Flore de la Nouvelle-Calédonie et dépendances. Dilleniaceae, Goodeniaceae, Iridaceae, Campynemataceae, volume 16. Publisher: Muséum National d'Histoire Naturelle, Paris. ISBN 2-85654-188-7 [140 pages][pdf][fr][NewCaledonia]

488 Jérémie, Joël - Mabberley, D. J. - Morat, Ph. - Veillon, J. M. - Hallé, N (1988) Flore de la Nouvelle-Calédonie et dépendances. Hernandiaceae, Meliaceae, Oncothecaceae, Santalaceae, volume 15. Publisher: Muséum National d'Histoire Naturelle, Paris. ISBN 2-85654-184-4 [156 pages][pdf][fr][NewCaledonia]

489 McPherson, Gordon - Tirel, Christiane (1987) Flore de la Nouvelle-Calédonie et dépendances. Euphorbiaceae I : Euphorbioideae, Crotonoideae, Acalyphoideae, Oldfieldioideae, volume 14. Publisher: Muséum National d'Histoire Naturelle, Paris. ISBN 2-85654-180-1 [228 pages][pdf][fr][NewCaledonia]

490 Heine, Heino (1984) Flore de la Nouvelle-Calédonie et dépendances. Convolvulacées, volume 13. Publisher: Muséum National d'Histoire Naturelle, Paris. ISBN 2-85654-168-2 [96 pages][pdf][fr][NewCaledonia]

491 Nielsen, I. - Prance, Ghillean T. - Edmondson, John (1983) Flore de la Nouvelle-Calédonie et dépendances. Légumineuses-Mimosées, Chrysobalanacées, Plombaginacées, volume 12. Publisher: Muséum National d'Histoire Naturelle, Paris. ISBN 2-85654-166-6 [140 pages][pdf][fr][NewCaledonia]

492 Tirel, Christiane - Jérémie, Joël (1982) Flore de la Nouvelle-Calédonie et dépendances. Éléocarpacées, Monimiacées, Amborellacées, Athérospermatacées, Triméniacées, Chloranthacées, volume 11. Publisher: Muséum National d'Histoire Naturelle, Paris. ISBN 2-85654-162-3 184 pages][pdf][fr][NewCaledonia]

493 Boiteau, Pierre (1981) Flore de la Nouvelle-Calédonie et dépendances. Apocynacées, volume 10. Publisher: Muséum National d'Histoire Naturelle, Paris. ISBN 2-85654-160-7 [306 pages][pdf][fr][NewCaledonia]

494 Lescot, M. - Nooteboom, H.P. - Villiers, J.-F. (1980) Flore de la Nouvelle-Calédonie et dépendances. Flacourtiacées, Symplocacées, Icacinacées, Corynocarpacées, Olacacées,

volume 9. Publisher: Muséum National d'Histoire Naturelle, Paris. ISBN 2-85654-157-7 [192 pages][pdf][fr][NewCaledonia]
495 Hallé, Nicolas (1977) Flore de la Nouvelle-Calédonie et dépendances. Orchidacées, volume 8. Publisher: Muséum National d'Histoire Naturelle, Paris. ISBN 2-85654-152-6 [568 pages][pdf][fr][NewCaledonia]
496 Heine, Heino (1976) Flore de la Nouvelle-Calédonie et dépendances. Acanthacées, Bignoniacées, Boraginacées, Solanacées, volume 7. Publisher: Muséum National d'Histoire Naturelle, Paris. [216 pages][pdf][fr][NewCaledonia]
497 Virot, Robert (1975) Flore de la Nouvelle-Calédonie et dépendances. Épacridacées, volume 6. Publisher: Muséum National d'Histoire Naturelle, Paris. [164 pages][pdf][fr][NewCaledonia]
498 Kostermans, André J. G. H. (1974) Flore de la Nouvelle-Calédonie et dépendances. Lauracées, volume 5. Publisher: Muséum National d'Histoire Naturelle, Paris. [124 pages][pdf][fr][NewCaledonia]
499 Laubenfels, David J. de (1972) Flore de la Nouvelle-Calédonie et dépendances. Gymnospermes, volume 4. Publisher: Muséum National d'Histoire Naturelle, Paris. [168 pages][pdf][fr][NewCaledonia]
500 Brownlie, G. (1969) Flore de la Nouvelle-Calédonie et dépendances. Ptéridophytes, volume 3. Publisher: Muséum National d'Histoire Naturelle, Paris. [308 pages][pdf][fr][NewCaledonia]
501 Virot, Robert (1968) Flore de la Nouvelle-Calédonie et dépendances. Protéacées, volume 2. Publisher: Muséum National d'Histoire Naturelle, Paris. [256 pages][pdf][fr][NewCaledonia]
502 Aubréville, André (1967) Flore de la Nouvelle-Calédonie et dépendances, volume 1 : Sapotacées. Publisher: Muséum National d'Histoire Naturelle, Paris. [168 pages][pdf][fr][NewCaledonia]
503 Cheeseman T.F. (1925) Manual of the New Zealand Flora. Publisher: W.R.B. Oliver, Wellingtoin N.Z. [1472 pages][pdf][en][NewZealand]
504 Kirk, T., (1889) The Forest Flora of New Zealand. Wellignton by authority : George Didsbury, Government printer [706 pages][pdf][en][NewZealand]
505 Ghazanfar Shahina A. (2003) Flora of the Sultanate of Oman, volume 1, Piperaceae - Primulaceae. Scripta Botanica Belgica 25; Meise National Botanic Garden (Belgium). ISBN 90-72619-55-2 [274 pages][pdf][en][Oman]
506 Baehni Charles and Luciano Bernardi (1970) Flora of Peru. SAPOTACEAE. Publisher: Botanical Series, Field Museum of Natural History, Volume XIII, Part V-A, Number 3, publication 1087. [64 pages][pdf][en][Peru]
507 Mathias, Mildred E. and Lincoln Constance (1962) Flora of Peru. UMBELLIFERAE. Publisher: Botanical Series, Field Museum of Natural History, VOLUME XIII, PART V-A, NUMBER 1, publication 953. [110 pages][pdf][en][Peru]
508 Gibson Dorothy N. (1967) Flora of Peru. Hydrophyllaceae. Publisher: Botanical Series, Field Museum of Natural History, VOLUME XIII, PART V-A, NUMBER 2, publication 1032. [52 pages][pdf][en][Peru]
509 McVaugh Rogers (1958) Flora of Peru. MYRTACEAE. Publisher: Botanical Series, Field Museum of Natural History, VOLUME XIII, PART IV, NUMBER 2, publication 861. [274

pages][pdf][en][Peru]
510 Correll Donovan (1967) Flora of Peru. SOLANACEAE. Publisher: Botanical Series, Field Museum of Natural History, VOLUME XIII, PART V-B, NUMBER 2, publication 1015. [304 pages][pdf][en][Peru]
511 MacBride J. Francis (1951) Flora of Peru. EUPHORBIAGEAE. Publisher: Botanical Series, Field Museum of Natural History, VOLUME XIII, PART III-A, NUMBER 1, publication 680. [200 pages][pdf][en][Peru]
512 MacBride J. Francis (1961) Flora of Peru. BIGNONIACEAE. Publisher: Botanical Series, Field Museum of Natural History, VOLUME XIII, PART V-C, NUMBER 1, publication 930. [124 pages][pdf][en][Peru]
513 MacBride J. Francis (1962) Flora of Peru. SOLANACEAE. Publisher: Botanical Series, Field Museum of Natural History, VOLUME XIII, PART V-B, NUMBER 1, publication 951. [280 pages][pdf][en][Peru]
514 MacBride J. Francis (1959) Flora of Peru. HALORAGACEAE. Publisher: Botanical Series, Field Museum of Natural History, VOLUME XIII, PART V, NUMBER 1, publication 880. [552 pages][pdf][en][Peru]
515 Edwin, Gabriel (1971) Flora of Peru. SCROPHULARIACEAE. Publisher: Botanical Series, Field Museum of Natural History, VOLUME XIII, PART V-B, NUMBER 3, publication 1125. [276 pages][pdf][en][Peru]
516 Edwin, Gabriel (1971) Flora of Peru. SCROPHULARIACEAE. Publisher: Botanical Series, Field Museum of Natural History, VOLUME XIII, PART V-B, NUMBER 3, publication 1125. [276 pages][pdf][en][Peru]
517 Correll Donovan (1967) Flora of Peru. SOLANACEAE. Publisher: Botanical Series, Field Museum of Natural History, VOLUME XIII, PART V-B, NUMBER 2, publication 1125. [200 pages][pdf][en][Peru]
518 MacBride J. Francis (1960) Flora of Peru. PALMAE. Publisher: Botanical Series, Field Museum of Natural History, VOLUME XIII, PART I, NUMBER 2, publication 895. [108 pages][pdf][en][Peru]
519 MacBride J. Francis (1956) Flora of Peru. SAPINDACEAE. Publisher: Botanical Series, Field Museum of Natural History, VOLUME XIII, PART IIIA, NUMBER 2, publication 808. [468 pages][pdf][en][Peru]
520 Steele, Arthur Robert (1964) Flowers for the King. The Expedition of Ruiz and Pavon and the Flora of Peru. Publisher: Durham, North Carolina, Duke University Press. [410 pages][pdf][en][Peru]
521 Jacob de Cordemoy E. (1891) Flore de l'Ile de la Réunion, Fascicule I, Cryptogames vasculaires (Fougères, Lycopodes, Sélaginelles). Saint Denis Réunion. [121 pages][pdf][fr][Reunion]
522 Jacob de Cordemoy E. (1895) Flore de l'Ile de la Réunion, Phanérogames, Cryptogames vasculaires, Muscinées avec l'indication des propriétés économiques & industrielles des plantes. Saint Denis Réunion. [614 pages][pdf][fr][Reunion]
523 Maevsky P.F. (2014) Flora of European part of Russia . Publisher: Tovarishestvo nauchnych izdaniy KMK , Moskva (Маевский П.Ф. . Флора Европейской части России. Издательство: Товарищество научных изданий КМК, Москва.) [636 pages][pdf][fr][Reunion]

524 Steyermark, Julian (1951-1952) Botanical Exploration in Venezuela I,II. Fieldiana: Botany, a Continuation of the Botanical Series of Field Museum of Natural History. Volume 28 Numbers 1 and 2. Publisher: Chicago Natural History Museum, Chicago, U.S.A. [268 pages][pdf][en][Venezuela]

525 Steyermark, Julian (1953-1957) Botanical Exploration in Venezuela III,IV. From Ericaceae through Compositae. Fieldiana: Botany, a Continuation of the Botanical Series of Field Museum of Natural History. Volume 28 Numbers 3 and 4. Publisher: Chicago Natural History Museum, Chicago, U.S.A. [572 pages][pdf][en][Venezuela]

526 Hu, Chi Ming - Vidal, Jules E. (2004) Flore du Cambodge, du Laos, et du Vietnam. Fascicules 32 : Myrsinaceæ, vol. 32. Muséum National d'Histoire Naturelle, Paris. ISBN 2-85654-218-2 [236 pages][pdf][fr][Vietnam]

527 Hul, Sovanmoly (2003) Flore du Cambodge, du Laos, et du Vietnam. Fascicule 31 : Gentianaceae, vol. 31, 2003 . Muséum National d'Histoire Naturelle, Paris. ISBN 2-85654-214-X [104 pages][pdf][fr][Vietnam]

528 Phan Kê Lôc - Vidal, Jules E. (2001) Flore du Cambodge, du Laos, et du Vietnam. Fascicules 30 : Leguminosæ-Papilionoideæ Millettieæ, vol. 30. Muséum National d'Histoire Naturelle, Paris. ISBN 2-85654-211-5 [196 pages][pdf][fr][Vietnam]

529 Niyomdham, C. - Pham Hoang Hô - Dy Phon, P. - Vidal, J. E. (1997) Flore du Cambodge, du Laos, et du Vietnam. Fascicule 29 : Leguminoseae-Papilionoïdeae Dalbergieae, vol. 29. Muséum National d'Histoire Naturelle, Paris. ISBN 2-85654-206-9 [72 pages][pdf][fr][Vietnam]

530 Hiêp, Nguyên Tiên - Vidal, Jules E. (1996) Flore du Cambodge, du Laos, et du Vietnam. Fascicules 28 : Gymnospermae Cycadaceae, Pinaceae, Taxodiaceae, Cupressaceae, Podocarpaceae, Cephalotaxaceae, Taxaceae, Gnetaceae., vol. 28 . Muséum National d'Histoire Naturelle, Paris. ISBN 2-85654-202-6 [172 pages][pdf][fr][Vietnam]

531 Dy Phon, Pauline - Ohashi, Hiroyoshi - Vidal, Jules E. (1994) Flore du Cambodge, du Laos, et du Vietnam. Fascicules 27 : Leguminosae (Fabaceae) Papilionoideae Desmodieae, vol. 27, 1994 . Muséum National d'Histoire Naturelle, Paris. ISBN 2-85654-199-2 [160 pages][pdf][fr][Vietnam]

532 Pham Hoang Hô - Chi-Ming Hu - Svengsuksa, Boua Khay Khone - Vidal, J. E. (1992) Flore du Cambodge, du Laos, et du Vietnam. Fascicule 26 : Rhoipteleaceae, Juglandaceae, Thymelaeaceae, Proteaceae. Primulaceae. Styracaceae, vol. 26. Muséum National d'Histoire Naturelle, Paris. ISBN 2-85654-194-1 [212 pages][pdf][fr][Vietnam]

533 Smitinand, T. - Vidal, Jules E. - Pham Hoang Hô (1990) Flore du Cambodge, du Laos, et du Vietnam. Fascicules 25 : Diptérocarpacées, vol. 25, 1990 . Muséum National d'Histoire Naturelle, Paris. ISBN 2-85654-189-5 [128 pages][pdf][fr][Vietnam]

534 Larsen, Kai (1989) Flore du Cambodge, du Laos, et du Vietnam. Fascicules 24 : Caryophyllales, vol. 24. Muséum National d'Histoire Naturelle, Paris. ISBN: 2-85654-179-X [264 pages][pdf][fr][Vietnam]

535 Nguyên Van Thuân, Phon, P. dy and Niyomdham, C. (1987) Flore du Cambodge, du Laos, et du Vietnam. Fascicule 23 : Légumineuses-Papilionoïdées vol. 23. Muséum National d'Histoire Naturelle, Paris [xxx pages][pdf][fr][Vietnam]

536 Santisuk, T. - Vidal, Jules E. (1985) Flore du Cambodge, du Laos, et du Vietnam. Fascicules 22 : Bignoniacées, vol. 22, 1985. Muséum National d'Histoire Naturelle, Paris. ISBN 2-

85654-175-5 [76 pages][pdf][fr][Vietnam]
537 Yamazaki, T. (1985) Flore du Cambodge, du Laos, et du Vietnam. Fascicule 21 : Scrophulariacées, vol. 21, 1985. Muséum National d'Histoire Naturelle, Paris. ISBN 2-85654-173-9 [224 pages][pdf][fr][Vietnam]
538 Stone, B. C. - Vu Van Cuong - Vidal, Jules E. - Van Bruggen, H. W. E. - Koyama, T. - Larsen, Kai - Hansen, B. (1983) Flore du Cambodge, du Laos, et du Vietnam. Fascicules 20 : Pandanacées, Sparganiacées, Ruppiacées, Potamogétonacées, Aponogétonacées, Smilacacées, Philydracées, Hanguanacées, Flagellariacées, Restionacées, Centrolépidacées, Lowiacées, Xyridacées., vol. 20, 1983 . Muséum National d'Histoire Naturelle, Paris. ISBN 2-85654-164-X [180 pages][pdf][fr][Vietnam]
539 Nielsen, I. (1981) Flore du Cambodge, du Laos, et du Vietnam. Fascicule 19 : Légumineuses - Mimosoïdées, vol. 19, 1981 . Muséum National d'Histoire Naturelle, Paris. ISBN 2-85654-161-5 [164 pages][pdf][fr][Vietnam]
540 Larsen, K. - Larsen, S. S. - Vidal, Jules E. (1980) Flore du Cambodge, du Laos, et du Vietnam. Fascicule 18 : Légumineuses - Césalpinioïdées, vol. 18. Muséum National d'Histoire Naturelle, Paris. ISBN 2-85654-158-5 [232 pages][pdf][fr][Vietnam]
541 Nguyên Van Thuân (1979) Flore du Cambodge, du Laos, et du Vietnam. Fascicule 17 : Légumineuses-Papilionoïdées Phaséolées, vol. 17. Muséum National d'Histoire Naturelle, Paris. ISBN 2-85654-156-9 [222 pages][pdf][fr][Vietnam]
542 Nooteboom, H. P. - Vidal, J. E. (1977) Flore du Cambodge, du Laos, et du Vietnam. Fascicule 16 : Symplocacées, vol. 16. Muséum National d'Histoire Naturelle, Paris. ISBN 2-85654-151-8 [80 pages][pdf][fr][Vietnam]
543 Keraudren-Aymonin, Monique (1975) Flore du Cambodge, du Laos, et du Vietnam. Fascicule 15 : Cucurbitacées, vol. 15. Muséum National d'Histoire Naturelle, Paris [128 pages][pdf][fr][Vietnam]
544 Kanis, A. - Vu Van Cuong - Vidal, J. E. - Hansen, B. - Cusset, C. (1973) Flore du Cambodge, du Laos, et du Vietnam. Fascicule 14 : Ochnacées, Onagracées, Trapacées, Balanophoracées, Rafflésicées, Podostémacées, Tristichacées, vol. 14. Muséum National d'Histoire Naturelle, Paris [100 pages][pdf][fr][Vietnam]
545 Tirel-Roudet, C. - Leeuwenberg, A. J. M. - Vidal, J. E. (1972) Flore du Cambodge, du Laos, et du Vietnam. Fascicule 13 : Loganiaceæ. Buddlejaceæ, vol. 13. Muséum National d'Histoire Naturelle, Paris [116 pages][pdf][fr][Vietnam]
546 Kubitzki, Klaus (1970) Flore du Cambodge, du Laos, et du Vietnam. Fascicule 12 : Hernandiaceae, vol. 12. Muséum National d'Histoire Naturelle, Paris [28 pages][pdf][fr][Vietnam]
547 Lescot, Michèle (1970) Flore du Cambodge, du Laos, et du Vietnam. Fascicule 11 : Flacourtiaceae, Bixaceae, Cochlospermaceae, vol. 11. Muséum National d'Histoire Naturelle, Paris [120 pages][pdf][fr][Vietnam]
548 Lecompte, O. (1969) Flore du Cambodge, du Laos, et du Vietnam. Fascicule 10 : Combretaceae, vol. 10. Muséum National d'Histoire Naturelle, Paris [128 pages][pdf][fr][Vietnam]
549 Nguyen Van Thuan (1969) Flore du Cambodge, du Laos, et du Vietnam. Fascicule 9 : Campanulaceae, vol. 9. Muséum National d'Histoire Naturelle, Paris [60 pages][pdf][fr][Vietnam]

550 Tardieu-Blot, M.-L. (1968) Flore du Cambodge, du Laos, et du Vietnam. Fascicule 8 : Nyssaceae, Cornaceae, Alangiaceae, vol. 8. Muséum National d'Histoire Naturelle, Paris [60 pages][pdf][fr][Vietnam]

551 Nguyen Van Thuan (1968) Flore du Cambodge, du Laos, et du Vietnam. Fascicule 7 : Rosaceae II (Rubus), vol. 7. Muséum National d'Histoire Naturelle, Paris [88 pages][pdf][fr][Vietnam]

552 Vidal, J. E. (1968) Flore du Cambodge, du Laos, et du Vietnam. Fascicule 6 : Rosaceae I (excl. Rubus), vol. 6. Muséum National d'Histoire Naturelle, Paris [216 pages][pdf][fr][Vietnam]

553 Tardieu-Blot, Marie-Laure - Cusset, G. (1967) Flore du Cambodge, du Laos, et du Vietnam. Fascicule 5 : Umbelliferae, Aizoaceae, Molluginaceae Passifloraceae, vol. 5. Muséum National d'Histoire Naturelle, Paris [164 pages][pdf][fr][Vietnam]

554 Lecompte, O. - Tardieu-Blot, Marie-Laure - Vu Van Cuong (1965) Flore du Cambodge, du Laos, et du Vietnam. Fascicule 4 : Saxifragaceae, Crypteroniaceae, Droseraceae, Hamamelidaceae, Haloragaceae, Rhizophoraceae, Sonneratiaceae, Punicaceae, vol. 4. Muséum National d'Histoire Naturelle, Paris [224 pages][pdf][fr][Vietnam]

555 Aubréville, A. and Tardieu-Blot(1963) Flore du Cambodge, du Laos, et du Vietnam. Fascicule 3 : Sapotacées, vol. 3 . Muséum National d'Histoire Naturelle, Paris [112 pages][pdf][fr][Vietnam]

556 Vidal, J. and Tardieu-Blot (1962) Flore du Cambodge du Laos et du Vietnam Fascicule 2 : Moringacées, Connaracées. Anacardiacées. Muséum National d'Histoire Naturelle, Paris 1960 [208 pages][pdf][fr][Vietnam]

557 Gagnepain, F. and Vidal, J. (1960) Flore du Cambodge, du Laos, et du Vietnam. Fascicule I : Sabiacées, vol. 1. Muséum National d'Histoire Naturelle, Paris [64 pages][pdf][fr][Vietnam]
